# Heterocycles and a Sorbicillinoid from the Coral-Derived Fungus *Penicillium chrysogenum*

**DOI:** 10.3390/md22110517

**Published:** 2024-11-15

**Authors:** Junjie Yang, Yuan Zong, Cili Wang, Kai Li, Yue Zhang, Pinglin Li

**Affiliations:** 1 Key Laboratory of Marine Drugs, Chinese Ministry of Education, School of Medicine and Pharmacy, Ocean University of China, Qingdao 266003, China; yangjunjie_ouc@163.com (J.Y.); wangcili881@163.com (C.W.); 17854232177@163.com (K.L.); zhangyue_00803@163.com (Y.Z.); 2 Laboratory of Marine Drugs and Biological Products, National Laboratory for Marine Science and Technology, Qingdao 266235, China; 3 Shandong Academy of Chinese Medicine, Jinan 250014, China; 17854201356@163.com; 4 Key Laboratory of Marine Food Processing & Safety Control, College of Food Science and Engineering, Ocean University of China, Qingdao 266003, China

**Keywords:** coral-derived fungus, *Penicillium chrysogenum*, heterocycles, sorbicillinoid, cytotoxicity

## Abstract

A detailed chemical study of the culture of a coral-derived fungus *Penicillium chrysogenum* resulted in the isolation and identification of four new aromatic heterocycles chrysoquinazolinones A–B (**1**–**2**) and chrysobenzothiazoles A–B (**3**–**4**), along with a new sorbicillinoid 4-carboxylsorbicillin (**5**). Chrysoquinazolinones A–B (**1**–**2**) combine a quinazolinone fragment with a bicyclo[2.2.2]octane or a pyrrolidone moiety, respectively, demonstrating the unexpected structures of marine natural products. Chrysobenzothiazoles A–B (**3**–**4**) possess a benzothiazole system and are the second isolation of this class of skeleton compounds from marine organisms. The existence of the pair of enantiomers (±**3**) was deduced by chiral HPLC analysis. Their structures and absolute configurations were elucidated by detailed spectroscopic analysis, comparison with the literature data, single-crystal X-ray crystallographic analysis and TDDFT-ECD calculations. Compound **5** exhibited moderate cytotoxicity against K562 and NCI-H446 cell lines, with IC_50_ values of 15.00 μM and 16.87 μM, respectively.

## 1. Introduction

Marine-derived filamentous fungi, which can produce bioactive secondary metabolites with unique structures, are recognized as valuable sources for the discovery of marine natural products [1]. As one of the most widespread fungal species, *Penicillium chrysogenum* has drawn considerable attention with medicinally valuable and structurally novel metabolites. As reported in the literature, the bioactive natural products from marine-derived *P. chrysogenum* are comprised of mono- and dimeric sorbicillinoids [2,3,4], alkaloids [5,6,7], polyketides [8,9,10], cerebrosides [11], xanthones [12,13], flavones [14], and terpenoids [15,16], which have wide range of biological activities, such as antitumor [2,6,12], antiviral [3,9], anti-inflammatory [13] and antibacterial [11,15].

**Figure 1:**
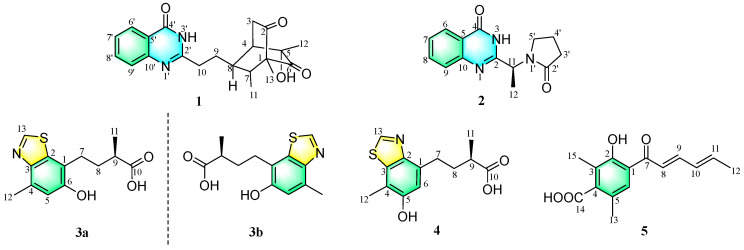
Structures of **1**–**5**.

As described in the literature above, the types of the bioactive natural products are various and unpredictable, demonstrating the high research value of fungus *P. chrysogenum*. In our ongoing search to discover novel natural products, the orthogonal experiment was conducted to determine the optimal culture condition of the coral-derived fungus *P. chrysogenum*, collected from the Xisha Islands in the South China Sea. Then, a chemical investigation of the extracts of fermentation broth and mycelia was conducted, which resulted in the isolation and identification of five new compounds, including two quinazolinone alkaloids chrysoquinazolinones A–B (**1**–**2**), two benzothiazole heterocycles chrysobenzothiazoles A–B (**3**–**4**), and a sorbicillinoid derivative 4-carboxylsorbicillin (**5**). Natural quinazolinones are extensively derived from terrestrial as well as marine plants, animals, and microorganisms, which have been widely reported with various biological activities [17]. Dimeric sorbicillinoids containing a bicyclo[2.2.2]octane moiety usually have been isolated from the fungi of the family *Penicillium* [3,18,19,20,21] and *Trichoderma* [22,23]. However, the unexpected combination of a quinazolinone fragment and a bicyclo[2.2.2]octane moiety in chrysoquinazolinone A (**1**) has never been found before, nor the conjunction of a quinazolinone fragment and a pyrrolidone moiety in chrysoquinazolinone B (**2**). Numerous benzothiazole derivatives with a wide spectrum of biological activity have been synthesized through various synthetic pathways, as these compounds have unique flexibility and dynamic cores [24,25,26]. Compared to the large number of synthetic benzothiazole derivatives, fewer benzothiazole heterocyclic compounds have been found from natural sources. Chrysobenzothiazoles A–B (**3**–**4**) are the second isolation of this class of compounds from marine organisms [27]. Herein, we report the details for isolation, structure elucidation, and bioactivities of these isolated compounds (Figure 1).

## 2. Results

**Figure 2:**
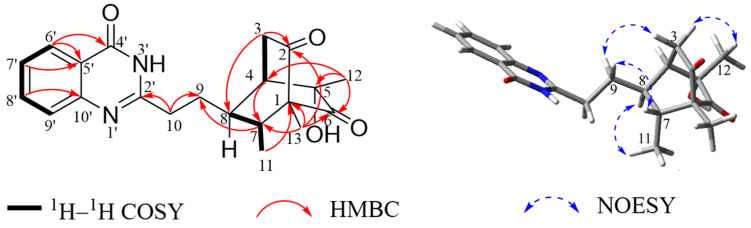
Key ^1^H–^1^H COSY, HMBC and NOESY correlations of **1**.

Chrysoquinazolinone A (**1**) was obtained as colorless crystals. Its molecular formula was determined as C_21_H_24_O_4_N_2_ based on the HRESIMS data (*m*/*z* 369.1813 [M + H]^+^, calcd. 369.1809), indicating eleven degrees of unsaturation. The ^13^C NMR data (Table 1) and HSQC spectrum showed the existence of 21 carbons, including 3 methyl, 3 methylene, 7 methine (4 olefinic), and 8 quaternary carbons. The ^1^H NMR data (Table 1) and HSQC spectrum showed 22 non-exchangeable protons, including 4 olefinic protons at *δ*_H_ 8.26 (1H, d, *J* = 7.8 Hz, H-6′), *δ*_H_ 7.50 (1H, m, H-7′), *δ*_H_ 7.79 (1H, m, H-8′), and *δ*_H_ 7.71 (d, *J* = 8.1 Hz, H-9′), 3 methyl groups at *δ*_H_ 0.94 (3H, d, *J* = 6.8 Hz, H_3_-11), *δ*_H_ 1.13 (3H, s, H_3_-12), and *δ*_H_ 1.07 (3H, s, H_3_-13), 6 methylene protons at *δ*_H_ 2.57 (1H, d, *J* = 19.5 Hz, H-3a), *δ*_H_ 2.91 (1H, overlapped, H-3b) *δ*_H_ 2.19 (1H, m, H-9a), *δ*_H_ 1.96 (1H, m, H-9b), *δ*_H_ 2.96 (1H, m, H-10a), and *δ*_H_ 2.89 (1H, overlapped, H-10b), and 3 sp^3^ hybridized methine protons at *δ*_H_ 2.40 (1H, s, H-4), *δ*_H_ 1.63 (1H, m, H-7), and *δ*_H_ 2.12 (1H, m, H-8). In addition, the existence of an exchangeable proton and a hydroxyl group was verified by the molecular formula. The planar structure of **1** was deduced by detailed analysis of ^1^H–^1^H COSY and HMBC spectra (Figure 2). The ^1^H–^1^H COSY correlations revealed the presence of three spin systems H_2_-3/H-4, H_3_-11/H-7/H-8, and H-6′/H-7′/H-8′/H-9′. The HMBC correlations from H_3_-13 to C-1, C-2, C-6, and C-7, H_3_-12 to C-4, C-5, and C-6, H_3_-11 to C-1, H-3a to C-2 and C-8, and H-4 to C-7 established a bicyclo[2.2.2]octane moiety. Moreover, the characteristic carbon signals [28] (156.0, 164.6, 120.6, 126.3, 126.9, 135.2, 127.4, 149.5), as well as the HMBC correlations from H-6′ to C-4′ and C-5′, H-7′, to C-5′, and H-8′ to C-10′, suggested a quinazolinone moiety. HMBC correlations from H-7 to C-9, and Ha-10 to C-9 and C-2′ indicated that the bicyclo[2.2.2]octane moiety was linked to the quinazolinone moiety via two linked methylene groups, completing the planar structure construction of **1**.

**Figure 3:**
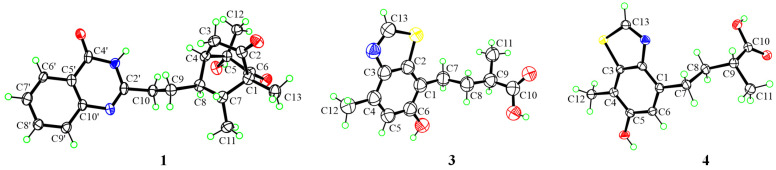
ORTEP drawing of **1**, **3,** and **4** (displacement ellipsoids are drawn at the 50% probability level).

The relative configuration of **1** was revealed by NOSEY correlations (Figure 2). The NOSEY correlation between H-7 and H-9a and the correlation between H-8 and H_3_-11 verified the different orientations of H-7 and H-8. In addition, the NOSEY correlation between H-3a and H_3_-12 and the correlation between H-3b and H-9b disclosed the relative configuration of **1** is 1R*, 4R*, 5S*, 7R*, and 8R*. Fortunately, suitable single crystals were acquired and subjected to a Bruker APEX-II CCD diffractometer with Cu K*α* radiation, validating the absolute configuration of **1** was 1*R*, 4*R*, 5*S*, 7*R*, and 8*R*, with a Flack parameter of −0.23 (16) (Figure 3).

**Figure 4:**
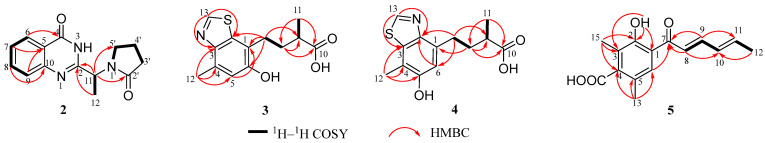
Key ^1^H–^1^H COSY and HMBC correlations of **2**–**5**.

**Figure 5:**
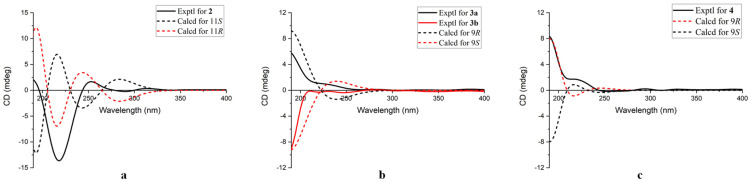
Experimental and calculated ECD spectra of **2**–**4** (**a**–**c**).

Chrysoquinazolinone B (**2**) was obtained as a colorless oil. The molecular formula was deduced to be C_14_H_15_O_2_N_3_ according to the HRESIMS data at *m*/*z* 258.1242 [M + H]^+^ (calcd. 258.1237). The ^13^C NMR data (Table 2) and HSQC spectrum showed 14 carbon resonances, including 1 methyl, 3 methylene, 5 methine (4 olefinic), and 5 quaternary carbons. The ^1^H NMR data (Table 2) showed 14 non-exchangeable protons, including 4 olefinic protons at *δ*_H_ 8.20 (1H, d, *J* = 8.0 Hz, H-6), *δ*_H_ 7.53 (1H, m, H-7), *δ*_H_ 7.82 (1H, m, H-8), and *δ*_H_ 7.71 (1H, d, *J* = 8.1 Hz, H-9), 1 methyl group at *δ*_H_ 1.63 (3H, d, *J* = 7.2 Hz, H_3_-12), 6 sp^3^ hybridized methylene protons at *δ*_H_ 2.47 (2H, t, *J* = 8.1 Hz, H_2_-3′), *δ*_H_ 2.14 (2H, m, H_2_-4′), and *δ*_H_ 3.65 (2H, m, H_2_-5′), as well as 1 sp^3^ hybridized methine proton at *δ*_H_ 5.13 (1H, q, *J* = 7.2 Hz, H-11). According to the molecular formula, one additional exchangeable proton is needed. The ^1^H–^1^H COSY and HMBC spectra (Figure 4) revealed the planar structure of **2**. Three spin systems H-6/H-7/H-8/H-9, H_3_-12/H-11, and H-3′/H-4′/H-5′ were deduced by the ^1^H–^1^H COSY spectrum. The characteristic carbon signals (156.6, 164.3, 122.4, 127.0, 128.1, 135.8, 128.6, 150.1) showed a quinazolinone moiety, which further determined by HMBC correlations from H-6 to C-4, H-7 to C-5, H-8 to C-10, and H-9 to C-5. Additionally, the HMBC correlations from H-11 to C-2, C-2′, C-5′, and H_3_-12 to C-2 suggested a pyrrolidone fragment, which was linked to the quinazolinone moiety by C-11, completing the planar structure construction of **2**. The absolute configuration of **2** was determined as 11*R* by further comparison of the experimental and the calculated ECD spectra (Figure 5).

Chrysobenzothiazole A (**3**) was isolated as colorless crystals. Its molecular formula was elucidated as C_13_H_15_O_3_NS on the basis of the HRESIMS [M + H]^+^ data at *m*/*z* 266.0846 (calcd. 266.0845), indicating six degrees of unsaturation. The ^13^C NMR data (Table 3) and HSQC spectrum showed the presence of 13 carbons, including 2 methyl, 2 methylene, 3 methine, and 6 quaternary carbons. The ^1^H NMR data (Table 3) and HSQC spectrum showed 13 protons, including 2 olefinic protons at *δ*_H_ 6.88 (1H, s, H-5) and *δ*_H_ 8.95 (1H, s, H-13), 2 methyl groups at *δ*_H_ 1.24 (3H, d, *J* = 7.0 Hz, H_3_-11) and *δ*_H_ 2.64 (3H, s, H_3_-12), 4 sp^3^ hybridized methylene protons at *δ*_H_ 2.87 (2H, m, H_2_-7), *δ*_H_ 2.02 (1H, m, H-8a), and *δ*_H_ 1.75 (1H, m, H-8b), together with 1 sp^3^ hybridized methine proton at *δ*_H_ 2.48 (1H, m, H-9). Moreover, two additional hydroxyl groups were needed to satisfy the molecular formula. The 1D NMR data [*δ*_C_ 118.9, 136.8, 146.9, 132.2, 117.5, 154.2 and 152.1; *δ*_H_ 6.88 (1H, s) and 8.95 (1H, s)] suggested a trisubstituted benzothiazole moiety, which was further verified by the HMBC correlations. The HMBC correlations (Figure 4) from H-13 to C-2 and C-3, H_3_-12 to C-3, C-4 and C-5, H-5 to C-1 indicated a methyl linking to C-4 and the connections of C-13 to C-2 via S, and C-13 to C-3 via N, which were further deduced by downfield chemical shift of C-2 (*δ*_C_ 136.8) and C-3 (*δ*_C_ 146.9). A hydroxyl group was connected to C-6 based on its downfield chemical shift (*δ*_C_ 154.2). Moreover, the ^1^H–^1^H COSY correlations (Figure 4) H_2_-7/H_2_-8 and H_3_-11/H-9, as well as the HMBC correlations from H_2_-7 to C-1, C-2, C-6, and C-9, H_2_-8 to C-9 and C-10, and H_3_-11 to C-8 and C-10 constructed the planar structure of **3**, in which another hydroxyl group was assigned to C-10.

To verify the absolute configuration of **3,** an X-ray crystallographic measurement was performed (Figure 3). The existence of enantiomers was confirmed by detailed analysis of the X-ray diffraction data without a Flack parameter (Table S4). Then, the racemate was subjected to chiral HPLC analysis. The result showed two peaks with the same UV absorption and good resolution in the HPLC chromatogram (Figure S5). The corresponding enantiomers **3a** and **3b** were obtained by using chiral HPLC, with [*α*]^25^_D_ = −1.57 (*c* 0.03, MeOH) and [*α*]^25^_D_ = +1.30 (*c* 0.03, MeOH), respectively. The absolute configuration of **3a** was determined as (−)-9*R*-**3** while the absolute configuration of **3b** was determined as (+)-9*S*-**3** through the comparison of experimental and calculated ECD spectra (Figure 5).

Chrysobenzothiazole A (**4**) was isolated as colorless crystals. Its molecular formula was determined as C_13_H_15_O_3_NS based on the HRESIMS [M + H]^+^ data at *m*/*z* 266.0847 (calcd. 266.0845), which was identical to **3**. The characteristic 1D NMR signals (Table 3) [*δ*_C_ 135.8, 146.3, 137.6, 115.1, 154.2, 116.5, and 151.9; *δ*_H_ 6.90 (1H, s) and 8.93 (1H, s)] revealed a trisubstituted benzothiazole moiety in **4**, the same as **3**. The ^13^C NMR data showed resonances of C-2 at *δ*_C_ 146.3 and C-3 at *δ*_C_ 137.6, while the corresponding signals in **3** at *δ*_C_ 136.8 and *δ*_C_ 146.9, indicating the opposite positions of N and S. The HMBC correlations (Figure 4) from H-13 to C-2 and C-3, H_3_-12 to C-3, C-4 and C-5, H-6 to C-2, and H-7 to C-2, and C-6 verified a methyl linking to C-4 and a hydroxyl group linking to C-5, which was further confirmed by the downfield chemical shift of C-5 (*δ*_C_ 154.2). Analysis of the rest ^1^H–^1^H COSY (Figure 4) and HMBC correlations established the planar structure of **4**. The differences in the planar structures between **3** and **4** are the positions of N, S, and the hydroxyl group in the benzothiazole moiety.

The ECD calculations were performed to verify the absolute configuration of **4**, which was determined as 9*R* (Figure 5). Suitable crystals for X-ray crystallographic analysis were obtained. A detailed analysis of X-ray data suggested that the absolute configuration of **4** was 9*R* (Figure 3), which showed the same conclusion as the ECD calculations.

**Figure 6:**
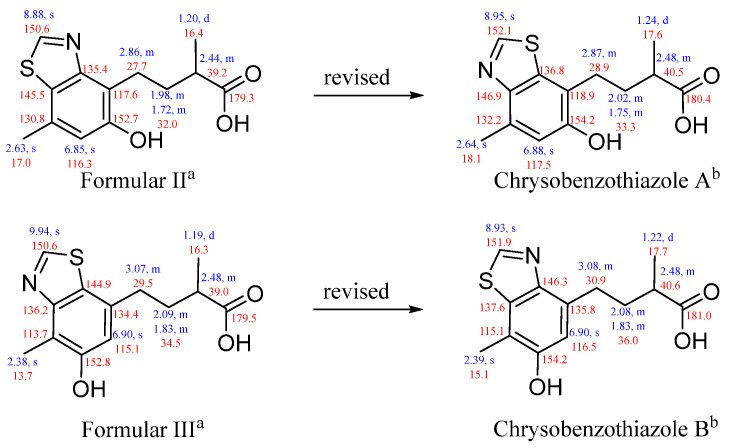
^1^H and ^13^C NMR data for formulars II–III and chrysobenzothiazoles A–B (^a^ Recorded in CD_3_OD. ^b^ Recorded in CD_3_OD, ^1^H NMR at 500 MHz and ^13^C NMR at 125 Hz).

As the second isolation of this class of skeleton compounds, compounds **3**–**4** were compared with formulars II–III, which were isolated from a deep-sea fungus *Penicillium allii-sativi*. There was a puzzle about the positions of N and S in the benzothiazole moiety. The ^1^H and ^13^C NMR data for **3**–**4** agreed perfectly with those for II–III. As mentioned above, the positions of N and S in **3**–**4** were undoubtedly verified by the downfield chemical shift of C-2 and C-3, as well as the X-ray data. Therefore, the planar structures of formulars II–III should be revised (Figure 6).

4-Carboxylsorbicillin (**5**) was isolated as yellow powder. Its molecular formula was elucidated as C_15_H_16_O_4_ based on the HRESIMS [M + H]^+^ data at *m*/*z* 261.1127 (calcd. 261.1121). The ^13^C NMR data (Table 4) and HSQC spectrum suggested 15 carbon resonances, including 3 methyl, 5 methine, and 7 quaternary (2 carbonyl) carbons. The ^1^H NMR data (Table 4) showed 14 protons, including 5 olefinic protons at *δ*_H_ 7.51 (1H, s, H-6), *δ*_H_ 7.00 (1H, d, *J* = 14.9 Hz, H-8), *δ*_H_ 7.53 (1H, overlapped, H-9), *δ*_H_ 6.38 (1H, overlapped, H-10), and *δ*_H_ 6.37 (1H, overlapped, H-11), 3 methyl groups at *δ*_H_ 1.94 (3H, d, *J* = 5.3 Hz, H_3_-12), *δ*_H_ 2.37 (3H, s, H_3_-13), and *δ*_H_ 2.31 (3H, s, H_3_-15), along with a phenolic hydroxyl group at *δ*_H_ 13.09 (1H, s). Moreover, one additional hydroxyl group was needed to satisfy the molecular formula. The ^1^H–^1^H COSY correlations (Figure 4) revealed the presence of two spin systems H-8/H-9/H-10 and H-11/H_3_-12. Evidently, the 1D NMR data [*δ*_C_ 119.7, 160.0, 124.8, 139.6, 123.6, 128.2; *δ*_H_ 7.51 (1H, s)] belonged to a penta-substituted benzene ring. The positions of substituents on the benzene ring were determined by relevant HMBC correlations (Figure 4) from H_3_-13 to C-4, C-5 and C-6, H_3_-15 to C-2, C-3, and C-4, and –OH to C-1, C-2, and C-3. In addition, a carboxyl group (*δ*_C_ 171.8) was assigned to C-4 on the basis of the molecular formula. The characteristic proton signals at *δ*_H_ 7.53, 7.00, 6.38, 6.37, and 1.94 suggested a sorbyl group, which was further verified by the HMBC correlations from H-8 to C-7 and C-10, H-9 to C-7 and C-11, and H_3_-12 to C-10. The sorbyl fragment was linked to C-1 according to the HMBC correlation from H-6 to C-7, completing the planar structure construction of compound **5**.

The configuration of the Δ^8,9^ was substantiated as *E* according to the large coupling constants of ^3^*J*_H-8,H-9_ (14.9 Hz). However, the coupling constants of ^3^*J*_H-10,H-11_ were indeterminate because of the overlapped signals of H-10 and H-11 in the ^1^H NMR spectrum. As reported in the literature [29], *E* configurations of double bonds are structural features in sorbicillinoids, therefore, the configuration of the Δ^10,11^ was deduced to be *E*. Compare with the reported compound sorbicillin [30], the structure of compound **5** shows that the hydroxyl group attached to C-4 is substituted by a carboxyl group.

As discussed above, the quinazolinone, benzothiazole, and sorbicillinoid derivatives exhibited a wide range of biological activities, including cytotoxicity. Thus, cytotoxic assays of compounds **1**–**5** were conducted. The results showed that compound **5** exhibited moderate cytotoxic activities against K562 and NCI-H446 cell lines, with IC_50_ values of 15.00 μM and 16.87 μM, respectively. Unfortunately, the other compounds did not show any cytotoxic activity (Table 5).

## 3. Materials and Methods

### 3.1. General Experimental Procedures

The optical rotation data were obtained on a Jasco P-1020 polarimeter (Jasco, Tokyo, Japan). The UV spectra and circular dichroism data were measured on a Jasco J-815 CD spectropolarimeter (Jasco, Tokyo, Japan), and IR spectra were recorded with a Nicolet NEXUS 470 spectrophotometer (Thermo Scientific, Beijing, China) in KBr disks. The NMR spectra were measured in CDCl_3_ or CD_3_OD on an Agilent DD2-500 (^1^H, 500 MHz; ^13^C, 125 MHz; Agilent, Beijing, China). The 7.26 and 77.2 ppm resonances of CDCl_3_, as well as the 3.31 and 49.0 ppm resonances of CD_3_OD, were used as internal references for ^1^H and ^13^C NMR spectra, respectively. HRESIMS data were obtained on Micromass Q-Tof Ultima GLOBAL GAA076LC mass spectrometer (Autospec-Ultima-TOF, Waters, Shanghai, China). The crystallographic data were acquired on a Bruker APEX-II CCD diffractometer (Bruker, Beijing, China) equipped with graphite-monochromatized Cu Kα radiation [31]. Analytic HPLC was performed by a Shimadzu LC-20AT liquid chromatograph (Shimadzu, Shanghai, China) equipped with an LC-20A array detector, using an analytic reversed-phased column (Shimadzu, ODS, 5 μm, 150 × 4.6 mm, 1.0 mL/min) or an analytic chiral-phase column (Daicel, Shanghai, China, IC, 5 μm, 250 × 4.6 mm, 1.0 mL/min). The semi-preparative HPLC was implemented by an Agilent 1100 series liquid chromatograph (Agilent, Tokyo, Japan) equipped with a DAD G1315A detector, using a semi-preparative reversed-phased column (SilGreen ODS, Beijing, China, 5 μm, 250 × 10 mm, 2.0 mL/min). Silica gel (300–400 mesh, Qingdao Marine Chemical Factory, Qingdao, China) was used for column chromatography (CC). Sephadex LH-20 (Amersham Pharmacia Biotech AB, Uppsala, Sweden) was used for molecular exclusion chromatography. Analytical TLC plates were precoated silica gel plates (GF254).

### 3.2. Fungal Material

The fungal strain 19-7-ZM-4 was isolated from an unidentified soft coral (190721-07) collected from the Xisha Islands in the South China Sea and identified as *Penicillium chrysogenum* according to the sequencing of the ITS region (GenBank accession number PP347996.1) with 100% similarity to *P*. *chrysogenum*. The strain was deposited at the State Key Laboratory of Marine Drugs, Ocean University of China.

### 3.3. Fermentation, Extraction and Isolation

The fungal strain 19-7-ZM-4 was initially cultured on the panels of PDA medium at 28 °C for 3 days. The fresh mycelia were inoculated into 500 mL Erlenmeyer flasks each containing 150 mL of the seed medium, comprised of dextrose (20 g/L), potato (200 g/L), and seawater (Huiquan Bay, Yellow Sea). The flasks were grown on a rotary shaker (180 rpm) at 28 °C for 24 h. Then, 1.0 mL seed culture was transferred into each 500 mL Erlenmeyer flask containing 150 mL of liquid culture medium, composed of dextrose (20 g/L), maltose (20 g/L), yeast extract powder (5 g/L), peptone (10 g/L), corn steep liquor (10 g/L), KH_2_PO_4_ (0.5 g/L), MgSO_4_ (0.5 g/L), and seawater (Huiquan Bay, Yellow Sea). These flasks were cultured on rotary shakers (180 rpm) at 28 °C for 13 days.

The fermented whole broth (40 L) was filtered under reduced pressure to separate the supernatant from mycelia. The supernatant was extracted three times with EtOAc, while the mycelia were macerated and extracted three times with MeOH. All the extracts were evaporated under reduced pressure and desalinated to yield 234.6 g of residue.

The extract was subjected to silica gel vacuum liquid chromatography, using a step gradient of petroleum ether/acetone (from 50/1 to 1/1, *v*/*v*) and CH_2_Cl_2_/MeOH (from 20/1 to 0/1, *v*/*v*). Based on the TLC analysis, 8 fractions (Fr.1-Fr.8) were acquired. Fraction Fr.5 (2.41 g) was subjected to silica gel CC eluting with petroleum ether/acetone (from 50/1 to 0/1, *v*/*v*) to yield six subfractions (Fr.5-1–5-6). Subfraction Fr.5-3 (120 mg) was purified by semi-preparative HPLC (ODS, 5 μm, 250 × 10 mm; CH_3_OH/H_2_O/HCOOH, 65:35:0.035, *v*/*v*/*v*; 2 mL/min) to obtain compound **5** (*t*_R_ = 60 min, 1.0 mg). Fraction Fr.6 (4.76 g) was subjected to silica gel CC eluting with petroleum ether/acetone (from 25/1 to 0/1, *v*/*v*) to acquire six subfractions (Fr.6-1–6-6). Subfraction Fr.6-5 (450 mg) was purified by semi-preparative HPLC (ODS, 5 μm, 250 × 10 mm; CH_3_OH/H_2_O/HCOOH, 65:35:0.035, *v*/*v*/*v*; 2 mL/min) to afford compounds **3** (*t*_R_ = 32 min, 1.8 mg) and **4** (*t*_R_ = 36 min, 1.5 mg). Then, **3** was analyzed and purified by chiral HPLC (IC, 5 μm, 250 ×10 mm; n-hexane/isopropanol, 80:20, *v*/*v*), which obtained **3a** (1.2 mg) and **3b** (0.3 mg). Fraction Fr.7 (5.31 g) was carried out by silica gel CC eluting with petroleum ether/acetone (from 5/1 to 0/1, *v*/*v*) to yield four subfractions (Fr.7-1–7-4). Compound **2** (*t*_R_ = 36 min, 7.0 mg) was purified from subfraction Fr.7-2 (2.06g) by Sephadex LH-20 column chromatography eluting with MeOH-CH_2_Cl_2_ (1:1, *v*/*v*) and semipreparative HPLC (ODS, 5 μm, 250 × 10 mm; CH_3_OH/H_2_O/HCOOH, 45:55:0.055, *v*/*v*/*v*; 2 mL/min). Subfraction Fr.7-3 (940 mg) was performed by Sephadex LH-20 column chromatography eluting with MeOH-CH_2_Cl_2_ (1:1, *v*/*v*) and semipreparative HPLC (ODS, 5 μm, 250 × 10 mm; CH_3_OH/H_2_O/HCOOH, 50:50:0.05, *v*/*v*/*v*; 2 mL/min) to afford compound **1** (*t*_R_ = 48 min, 4.8 mg).

Chrysoquinazolinone A (**1**): colorless crystals; [*α*]^25^_D_ −39.07 (*c* 0.10, MeOH); UV (MeOH) *λ*_max_ (log *ε*) 200.0 (1.86) nm, 224.5 (1.78) nm, 305.5 (0.28) nm; IR (KBr) *ν*_max_ 3410, 2832, 1594, 1363, 776 cm^−1^; HRESIMS *m*/*z* 369.1813 [M + H]^+^ (calcd. for C_21_H_25_O_4_N_2_, 369.1809). For ^1^H NMR and ^13^C NMR data, see Table 1.

Chrysoquinazolinone B (**2**): colorless oil; [*α*]^25^_D_ +2.53 (*c* 0.10, MeOH); UV (MeOH) *λ*_max_ (log *ε*) 199.0 (2.23), 224.5 (2.24), 266.0 (0.67), 302.0 (0.36) nm; ECD (0.25 mg/mL, CH_3_OH) *λ*_max_ (Δ*ε*) 218 (−13.63), 253 (1.66), 219 (−0.23) nm; IR (KBr) *ν*_max_ 2832, 2359, 1595, 1363, 775 cm^−1^; HRESIMS at *m*/*z* 258.1242 [M + H]^+^ (calcd. for C_14_H_16_O_2_N_3_ 258.1237). For ^1^H NMR and ^13^C NMR data, see Table 2.

Chrysobenzothiazole A (±**3**): colorless crystals; [*α*]^25^_D_ −1.57 (*c* 0.03, MeOH) for (−)-9*R*-**3**, [*α*]^25^_D_ +2.30 for (+)-9*S*-**3**; UV (MeOH) *λ*_max_ (log *ε*) 207.0 (1.75), 227.0 (1.16), 243.0 (1.19), 281.5 (0.56) nm for (−)-9*R*-**3**, UV (MeOH) *λ*_max_ (log *ε*) 207.0 (1.28), 227.0 (0.85), 243.0 (0.82), 281.5 (0.39) nm for (+)-9*S*-**3**; ECD (0.25 mg/mL, CH_3_OH) *λ*_max_ (Δ*ε*) 190 (5.79) nm for (−)-9*R*-**3**, ECD (0.50 mg/mL, CH_3_OH) *λ*_max_ (Δ*ε*) 190 (−9.04) nm for (+)-9*S*-**3**; IR (KBr) *ν*_max_ 3406, 2834, 2360, 2341, 1598, 1364, 777, 669 cm^−1^; HRESIMS at *m*/*z* 266.0846 [M + H]^+^ (calcd. for C_13_H_16_O_3_NS, 266.0845). For ^1^H NMR and ^13^C NMR data, see Table 3 and Table S1.

Chrysobenzothiazole A (**4**): colorless crystals; [*α*]^25^_D_ −2.70 (*c* 0.03, MeOH); UV (MeOH) *λ*_max_ (log *ε*) 207.0 (2.28), 225.0 (1.58), 244.5 (1.43), 279.5 (0.76) nm; ECD (0.50 mg/mL, CH_3_OH) *λ*_max_ (Δ*ε*) 190 (8.31), 208 (1.93) nm; IR (KBr) *ν*_max_ 3405, 2833, 2360, 2342, 1594, 1363, 777, 669 cm^−1^; HRESIMS at *m*/*z* 266.0847 [M + H]^+^ (calcd. for C_13_H_16_O_3_NS, 266.0845). For ^1^H NMR and ^13^C NMR data, see Table 3 and Table S2.

4-Carboxylsorbicillin (**5**): yellow powder; UV (MeOH) *λ*_max_ (log *ε*) 200.0 (2.24), 309.5 (2.43) nm; IR (KBr) *ν*_max_ 3406, 2360, 2341, 1595, 1364, 669 cm^−1^; HRESIMS at *m*/*z* 261.1127 [M + H]^+^ (calcd. for C_15_H_17_O_4_, 261.1121). For ^1^H NMR and ^13^C NMR data, see Table 4.

### 3.4. X-Ray Crystallographic Analysis

The crystals for chrysoquinazolinone A (**1**), chrysobenzothiazole A (±**3**) and B (**4**) were obtained from a solution of EtOH-H_2_O using the vapor diffusion method at 4 °C. X-ray crystallographic data were collected on a Bruker APEX-II CCD diffractometer with Cu-K*α* radiation. The crystals were kept at 293 K, 150 K, or 100 K during data collection. Crystallographic data have been deposited in the Cambridge Crystallographic Data Centre. The data can be obtained via https://www.ccdc.cam.ac.uk/ (accessed on 24 March 2022).

Crystal data for chrysoquinazolinone A (**1**): C_21_H_24_N_2_O_4_, M = 368.42, *a* = 13.1209(5) Å, *b* = 21.3061(7) Å, *c* = 13.3607(5) Å, *α* = 90°, *β* = 90.100(10)°, *γ* = 90°, *V* = 3735.0(2) Å^3^, T = 293(2) K, space group P2_1_, Z = 8, *μ* = 0.742 mm^−1^, *F*(000) = 1568. A total of 10,888 independent reflections [*R*_int_ = 0.0559, *R*_sigma_ = 0.0456] were used for the analysis. The final *R* indexes [all data] gave *R*_1_ =0.0694, w*R*_2_ = 0.1755. The goodness of fit on *F*^2^ was 1.028 and the Flack parameter was −0.23(16). CCDC 2342721.

Crystal data for chrysobenzothiazole A (±**3**): C_13_H_15_NO_3_S, M =265.32, *a* = 8.5937(3) Å, *b* = 9.1721(4) Å, *c* = 16.9210(7) Å, *α* = 90°, *β* = 93.851(2)°, *γ* = 90°, *V* = 1330.74(9) Å^3^, T = 150 K, space group P2_1_/n, Z = 4, *μ* = 2.174 mm^−1^, *F*(000) = 560. A total of 2724 independent reflections [*R*_int_ = 0.0506, R_sigma_ = 0.0512] were used for the analysis. The final R indexes [all data] gave R_1_ =0.0818, wR_2_ = 0.2000. The goodness of fit on F^2^ was 1.109. CCDC 2342719.

Crystal data for chrysobenzothiazole B (**4**): C_13_H_15_NO_3_S, M =265.32, *a* = 4.69830(10) Å, *b* = 15.3583(2) Å, *c* = 17.0647(3) Å, *α* = 90°, *β* = 90°, *γ* = 90°, *V* = 1231.35(4) Å^3^, T = 100 K, space group P2_1_2_1_2_1_, Z = 4, *μ* = 2.35 mm^−1^, *F*(000) = 560. A total of 2484 independent reflections [*R*_int_ = 0.0295, *R*_sigma_ = 0.0372] were used for the analysis. The final *R* indexes [all data] gave *R*_1_ =0.0335, w*R*_2_ = 0.0855. The goodness of fit on *F*^2^ was 1.071, and the Flack parameter was 0.020(11). CCDC 2342720.

### 3.5. Quantum Chemical Calculations

The conformational search of possible configurations whose energy was within 10 kJ/mol was carried out in an OPLS3e force field by MacroModel integrated into Maestro (version 11.9) (Schrödinger version 2019.1). The root mean squared distance (RMSD) cutoff of 0.5 Å and the maximum iteration of 2500 were executed to eliminate redundant conformers. Geometry optimizations and frequency calculations of stable conformers were performed at the B3LYP/6-31 G(d,p) level using density functional theory (DFT). Subsequently, a set of the lowest-energy conformers, whose Boltzmann distributions of Gibbs free energies were more than 1.0% were used for the next calculations. The ECD calculations were performed at the CAM-B3LYP/6-311G(d, p) level using the TD-DFT method with the IEFPCM solvent model for methanol in agreement with the experimental condition [32]. The Gaussian 09 package (version D. 01) was used for all the calculations.

### 3.6. Cytotoxicity Assay

The cytotoxic activity against the K562 (human leukemia) cell line was evaluated by MTT method [33], and the cytotoxic activities against L-02 (normal human hepatocytes), ASPC-1 (human pancreatic cancer), MDA-MB-231 (human breast cancer), NCI-H446, and NCI-H446/EP (human small cell lung cancer) cell lines were determined by SRB method [34]. The difference between the two human small-cell lung cancer cell lines is that NCI-H446 is a sensitive cell line while the NCI-H446/EP cell line is resistant to cisplatin, etoposide, SN38, and doxorubicin. As a positive control, doxorubicin was used.

## 4. Conclusions

In summary, two quinazolinone alkaloids chrysoquinazolinones A–B (**1**–**2**), two benzothiazole heterocycles chrysobenzothiazoles A–B (**3**–**4**), together with a sorbicillinoid derivative 4-carboxylsorbicillin (**5**), were isolated from the culture of a coral-derived fungus *Penicillium chrysogenum* collected from the Xisha Islands in the South China Sea. The structures of chrysoquinazolinones A–B (**1**–**2**) are unusual as they combined a quinazolinone system with a bicyclo[2.2.2]octane or a pyrrolidone moiety, respectively. Chrysobenzothiazoles A–B (**3**–**4**) containing a benzothiazole system were isolated as natural products from marine organisms for the second time. Chiral HPLC was carried out to obtain optically pure compounds (−)-9*R*-**3** (**3a**) and (+)-9*S*-**3** (**3b**) and their absolute configurations were elucidated by ECD calculations and single-crystal X-ray diffraction. Moreover, the structures of formulars II–III reported in the patent were revised. Finally, biological evaluation results revealed that compound **5** exhibited moderate cytotoxicity against K562 and NCI-H446 cell lines with IC_50_ values of 15.00 μM and 16.87 μM, respectively. The discovery of heterocyclic compounds enriched the structural diversity of this family. However, the biosynthetic pathway of these heterocyclic compounds is still uncertain, and the heterocycles need to be further tested for their bioactivities. The discovery of these novel compounds displayed high research value of fungus *P. chrysogenum* and enriched chemical libraries of marine fungi.

## Figures and Tables

**Table 1:** One-dimensional and two-dimensional NMR data for chrysoquinazolinone A (**1**) in CDCl_3._

Pos.	1				
	*δ*_C_ ^a^ Type	*δ*_H_ ^b^ (*J* in Hz)	^1^H–^1^H COSY	HMBC (H→C)	NOESY
1	66.4, C				
2	207.2, C				
3a	36.2, CH_2_	2.57, d (19.5)	H-4	C-2, 4, 5	H-12
3b		2.91 ^[α]^	H-4	C-2, 4, 8	H-9b
4	40.2, CH	2.40, s	H-3a, 3b	C-2, 5, 6, 7	
5	75.9, C				
6	211.8, C				
7	43.6, CH	1.63, m	H-8, 11	C-1, 2, 6, 8, 9, 11	H-9a
8	38.9, CH	2.12, m	H-7		H-11
9a	30.1, CH_2_	2.19, m			H-7
9b		1.96, m			H-3b
10a	33.8, CH_2_	2.96, m		C-9, 2′	
10b		2.89 ^[α]^		C-2′	
11	16.2, CH_3_	0.94, d (6.8)	H-7	C-1, 7, 8	H-8
12	33.4, CH_3_	1.13, s		C-4, 5, 6	H-3a
13	9.4, CH_3_	1.07, s		C-1, 2, 6, 7	
2′	156.0, C				
4′	164.6, C				
5′	120.6, C				
6′	126.3, CH	8.26, d (7.8)	H-7′	C-4′, 8′, 10′	
7′	126.9, CH	7.50, m	H-6′, 8′	C-5′, 9′	
8′	135.2, CH	7.79, m	H-7′, 9′	C-6′, 10′	
9′	127.4, CH	7.71, d (8.1)	H-8′	C-5′, 7′	
10′	149.5, C				

^a^ Recorded at 125 MHz. ^b^ Recorded at 500 MHz. ^[α]^ Overlapped signals.

**Table 2:** One-dimensional and two-dimensional NMR data for chrysoquinazolinone B (**2**) in CD_3_OD.

Pos.	2			
	*δ*_C_ ^a^ Type	*δ*_H_ ^b^ (*J* in Hz)	^1^H–^1^H COSY	HMBC (H→C)
2	156.6, C			
4	164.3, C			
5	122.4, C			
6	127.0, CH	8.20, d (8.0)	H-7	C-4, 8, 10
7	128.1, CH	7.53, m	H-6, 8	C-5, 9
8	135.8, CH	7.82, m	H-7, 9	C-6, 10
9	128.6, CH	7.71, d (8.1)	H-8	C-5, 7
10	150.1, C			
11	51.2, CH	5.13, q (7.2)	H-12	C-2, 12, 2′, 5′
12	15.9, CH_3_	1.63, d (7.2)	H-11	C-2, 11
2′	178.4, C			
3′	32.1, CH_2_	2.47, t (8.1)	H-4′	C-2′, 4′, 5′
4′	19.1, CH_2_	2.14, m	H-3′, 5′	C-2′, 3′, 5′
5′	45.5, CH_2_	3.65, m	H-4′	C-2′, 3′, 4′

^a^ Recorded at 125 MHz. ^b^ Recorded at 500 MHz.

**Table 3:** ^1^H and ^13^C NMR data for chrysobenzothiazole A (**3**) and B (**4**) in CD_3_OD.

Pos.	3		4	
	*δ*_C_ ^a^ Type	*δ*_H_ ^b^ (*J* in Hz)	*δ*_C_ ^a^ Type	*δ*_H_ ^b^ (*J* in Hz)
1	118.9, C		135.8, C	
2	136.8, C		146.3, C	
3	146.9, C		137.6, C	
4	132.2, C		115.1, C	
5	117.5, CH	6.88, s	154.2, C	
6	154.2, C		116.5, CH	6.90, s
7	28.9, CH_2_	2.87, m	30.9, CH_2_	3.08, m
8a	33.3, CH_2_	2.02, m	36.0, CH_2_	2.08, m
8b		1.75, m		1.83, m
9	40.5, CH	2.48, m	40.6, CH	2.48, m
10	180.4, C		181.0, C	
11	17.6, CH_3_	1.24, d (7.0)	17.7, CH_3_	1.22, d (7.0)
12	18.1, CH_3_	2.64, s	15.1, CH_3_	2.39, s
13	152.1, CH	8.95, s	151.9, CH	8.93, s

^a^ Recorded at 125 MHz. ^b^ Recorded at 500 MHz.

**Table 4:** ^1^H and ^13^C NMR data for **5** in CDCl_3._

Pos.	5			
	*δ*_C_ ^a^ Type	*δ*_H_ ^b^ (*J* in Hz)	^1^H–^1^H COSY	HMBC (H→C)
1	119.7, C			
2	160.0, C			
3	124.8, C			
4	139.6, C			
5	123.6, C			
6	128.2, CH	7.51, s		C-2, 4, 7, 13
7	194.2, C			
8	121.5, CH	7.00, d (14.9)	H-9	C-7, 10
9	146.5, CH	7.53 ^[α]^	H-8, 10	C-7, 10, 11
10	130.6, CH	6.38 ^[α]^	H-9	C-9, 12
11	143.0, CH	6.37 ^[α]^	H-12	C-9, 12
12	19.2, CH_3_	1.94, d (5.3)	H-11	C-10, 11
13	19.3, CH_3_	2.37, s		C-4, 5, 6
14	171.8, C			
15	12.8, CH_3_	2.31, s		C-2, 3, 4
	-OH	13.09, s		C-1, 2, 3

^a^ Recorded at 125 MHz. ^b^ Recorded at 500 MHz. ^[α]^ Overlapped signals

**Table 5:** Cytotoxic activities of **1**–**5**.

Compound	IC_50_ (μM)					
	K562	L-02	ASPC-1	MDA-MB-231	NCI-H446	NCI-H446/EP
**1**	>30	>30	>30	>30	>30	>30
**2**	>30	>30	>30	>30	>30	>30
**3a**	>30	>30	>30	>30	>30	>30
**3b**	>30	>30	>30	>30	>30	>30
**4**	>30	>30	>30	>30	>30	>30
**5**	15.00	>30	>30	>30	16.87	>30
Doxorubicin ^a^	<1	<1	>1	<1	<1	>1

^a^ Doxorubicin was used as positive control.

## Data Availability

Data are contained within the article or Supplementary Materials, further inquiries can be directed to the corresponding author.

## Supplementary Materials

The following supporting information can be downloaded at: https://www.mdpi.com/article/10.3390/md22110517/s1, Tables S1 and S2: NMR data of **3**–**4**; Tables S3–S5 and Figures S1–S4: X-ray crystallographic analysis of **1**, **3,** and **4**; Figures S5 and S6: Chiral HPLC chromatograms for **3** and **4**; Figures S7–S9 and Table S6: Stable conformers of **2**–**4**; Tables S7–S10: The gradient of media, HPLC and TLC chromatograms, and mass of residue; Tables S11–S13: The cytotoxicity of **1**–**5**. Figures S10–S45: The HRESIMS, UV, and NMR spectra of **1**–**5**.
