# Supplementary material for: Heterocycles and a Sorbicillinoid from the Coral-Derived Fungus Penicillium chrysogenum
Source: Mar Drugs. 2024 Nov 15;22(11):517. doi: 10.3390/md22110517 (PMC11595424; doi:10.3390/md22110517)
Supplement: Supplementary file 1 [file marinedrugs-22-00517-s001.zip › marinedrugs-3301566-supplementary.pdf]

Supporting Information

Heterocycles and a Sorbicillinoid from the Coral-Derived Fungus *Penicillium chrysogenum*

Junjie Yang [1,2], Yuan Zong [3], Cili Wang[1,2,4], Kai Li[1,2], Yue Zhang[1,2] and Pinglin Li [1,2,*]

[1] Key Laboratory of Marine Drugs, Chinese Ministry of Education, School of Medicine and Pharmacy, Ocean University of China, Qingdao 266003, China

[2] Laboratory of Marine Drugs and Biological Products, National Laboratory for Marine Science and Technology, Qingdao 266235, China

[3] Shandong Academy of Chinese Medicine, Jinan 250014, China

[4] Key Laboratory of Marine Food Processing & Safety Control, College of Food Science and Engineering, Ocean University of China, Qingdao 266003, China

*Correspondence: lipinglin@ouc.edu.cn; Tel.: +86-532-8203-3054

Table of Contents

1. 1D and 2D NMR Date for 1-5......5

Table S1.1D and 2D NMR data for chrysobenzothiazole A (**3**) in CD_3OD......5

Table S2.1D and 2D NMR data for chrysobenzothiazole B (**4**) in CD_3OD......5

2. X-ray crystallographic analyses of 1, 3 and 4......6

Table S3. X-ray diffraction analysis of chrysoquinazolinone A (**1**)......7

Table S4. X-ray diffraction analysis of chrysobenzothiazole A (±**3**)......8

Table S5. X-ray diffraction analysis of chrysobenzothiazole B (**4**)......9

Figure S1. Original ORTEP drawing of **1** (displacement ellipsoids are drawn at the 50% probability level)......10

Figure S2. ORTEP drawing of **1** (displacement ellipsoids are drawn at the 50% probability level)......10

Figure S3. ORTEP drawing of **3** (displacement ellipsoids are drawn at the 50% probability level)......11

Figure S4. ORTEP drawing of **4** (displacement ellipsoids are drawn at the 50% probability level)......11

Figure S5. Chiral HPLC chromatogram of ±**3**......12

Figure S6. Chiral HPLC chromatogram of **4**......12

3.Quantum chemical calculations......13

Figure S7. Stable conformers of compound **2**......13

Figure S8. Stable conformers of compound **3**......13

Figure S9. Stable conformers of compound **4**......14

Table S6. Optimized Z-Matrixes of compounds **2** – **4** in the Gas Phase (Å) at B3LYP/6-31G (d, p) level......14

4. Optimization of culture conditions......25

Table S7. Ingredient of media **1** – **3**......25

Table S8. HPLC chromatograms of residue......26

Table S9. TLC chromatograms of residue......27

Table S10. Mass of residue......27

5. Cytotoxicity Assay.......27

Table S11. Cytotoxic activities of **1–5**......28

Table S12. Cytotoxicity against K562 cell line of **5**......28

Table S13. Cytotoxicity against NCI-H446 cell line of **5**........28

6.The HRESIMS, UV and NMR spectra of 1 – 5........29

Figure S10. HRESIMS spectrum of compound **1.**........29

Figure S11. UV spectrum of compound **1.**........29

Figure S12. ^{1}H NMR spectrum of compound **1** in $CDCl_3$, 500MHz.........30

Figure S13. ^{13}C NMR spectrum of compound **1** in $CDCl_3$, 125MHz.........30

Figure S14. HSQC spectrum of compound **1** in $CDCl_3$, 500MHz.........31

Figure S15. HMBC spectrum of compound **1** in $CDCl_3$, 500MHz.........31

Figure S16. ^{1}H – ^{1}H COSY spectrum of compound **1** in $CDCl_3$, 500MHz.........32

Figure S17. NOESY spectrum of compound **1** in $CDCl_3$, 500MHz.........32

Figure S18. HRESIMS spectrum of compound **2.**........33

Figure S19. UV spectrum of compound **2.**........33

Figure S20. ^{1}H NMR spectrum of compound **2** in CD_3OD, 500MHz.........34

Figure S21. ^{13}C NMR spectrum of compound **2** in CD_3OD, 125MHz.........34

Figure S22. HSQC spectrum of compound **2** in CD_3OD, 500MHz.........35

Figure S23. HMBC spectrum of compound **2** in CD_3OD, 500MHz.........35

Figure S24. ^{1}H – ^{1}H COSY spectrum of compound **2** in CD_3OD, 500MHz.........36

Figure S25. HRESIMS spectrum of compound **3.**........36

Figure S26. UV spectrum of compound **3.**........37

Figure S27. ^{1}H NMR spectrum of compound **3** in CD_3OD, 500MHz.........37

Figure S28. ^{13}C NMR spectrum of compound **3** in CD_3OD, 125MHz.........38

Figure S29. HSQC spectrum of compound **3** in CD_3OD, 500MHz.........38

Figure S30. HMBC spectrum of compound **3** in CD_3OD, 500MHz.........39

Figure S31. ^{1}H – ^{1}H COSY spectrum of compound **3** in CD_3OD, 500MHz.........39

Figure S32. HRESIMS spectrum of compound **4.**........40

Figure S33. UV spectrum of compound **4.**........40

Figure S34. ^{1}H NMR spectrum of compound **4** in CD_3OD, 500MHz.........41

Figure S35. ^{13}C NMR spectrum of compound **4** in CD_3OD, 125MHz.........41

Figure S36. HSQC spectrum of compound **4** in CD_3OD, 500MHz.........42

Figure S37. HMBC spectrum of compound **4** in CD_3OD, 500MHz.........42

Figure S38. ^{1}H – ^{1}H COSY spectrum of compound **4** in CD_3OD, 500MHz.........43

Figure S39. HRESIMS spectrum of compound **5**. 43
Figure S40. UV spectrum of compound **5**. 44
Figure S41. ^{1}H NMR spectrum of compound **5** in $CDCl_3$, 500MHz. 44
Figure S42. ^{13}C NMR spectrum of compound **5** in $CDCl_3$, 125MHz.. 45
Figure S43. HSQC spectrum of compound **5** in $CDCl_3$, 500MHz. 45
Figure S44. HMBC spectrum of compound **5** in $CDCl_3$, 500MHz. 46
Figure S45. ^{1}H – ^{1}H COSY spectrum of compound **5** in $CDCl_3$, 500MHz. 46

1. 1D and 2D NMR Date for 1-5

Table S1.1D and 2D NMR data for chrysobenzothiazole A (**3**) in CD_3OD

Pos.	3			
	**δ_C[a] Type**	**δ_H[b] (*J* in Hz)**	**^{1}H–^{1}H COSY**	**HMBC (H→C)**
1	118.9, C			
2	136.8, C			
3	146.9, C			
4	132.2, C			
5	117.5, CH	6.88, s		C-1, 3, 6, 12
6	154.2, C			
7	28.9, CH_2	2.87, m	H-8a, 8b	C-1, 2, 6, 8, 9
8a	33.3, CH_2	2.02, m	H-7	C-1, 7, 9, 10, 11
8b		1.75, m	H-7	C-1, 7, 9, 10, 11
9	40.5, CH	2.48, m	H-11	C-7, 8, 10, 11
10	180.4, C			
11	17.6, CH_3	1.24, d (7.0)	H-9	C-8, 9, 10
12	18.1, CH_3	2.64, s		C-3, 4, 5
13	152.1, CH	8.95, s		C-2, 3

[a] Recorded at 125MHz. [b] Recorded at 500 MHz.

Table S2.1D and 2D NMR data for chrysobenzothiazole B (**4**) in CD_3OD

Pos.	4			
	**δ_C[a] Type**	**δ_H[b] (*J* in Hz)**	**^{1}H–^{1}H COSY**	**HMBC (H→C)**
1	135.8, C			
2	146.3, C			
3	137.6, C			
4	115.1, C			
5	154.2, C			
6	116.5, CH	6.90, s		C-2, 4, 5, 7
7	30.9, CH_2	3.08, m	H-8a, 8b	C-1, 2, 6, 8, 9
8a	36.0, CH_2	2.08, m	H-7	C-1, 7, 9, 11
8b		1.83, m	H-7	C-1, 7, 9, 10, 11
9	40.6, CH	2.48, m	H-11	C-7, 8, 11
10	181.0, C			
11	17.7, CH_3	1.22, d (7.0)	H-9	C-8, 9, 10
12	15.1, CH_3	2.39, s		C-3, 4, 5
13	151.9, CH	8.93, s		C-2, 3

[a] Recorded at 125MHz. [b] Recorded at 500 MHz.

2. X-ray crystallographic analyses of 1, 3 and 4

Chrysoquinazolinone A (**1**) was obtained as colorless crystals in an EtOH-H_2O solvent system using the vapor diffusion method. The crystallographic data for **1** in this article have been deposited at the Cambridge Crystallographic Data Centre under supplementary publication number 2342721. The data can be obtained via https://www.ccdc.cam.ac.uk/.

Chrysobenzothiazole A (**3**) was obtained as colorless crystals in an EtOH-H_2O solvent system using the vapor diffusion method. The crystallographic data for **3** in this article have been deposited at the Cambridge Crystallographic Data Centre under supplementary publication number 2342719. The data can be obtained via https://www.ccdc.cam.ac.uk/.

Chrysobenzothiazole B (**4**) was obtained as colorless crystals in an EtOH-H_2O solvent system using the vapor diffusion method. The crystallographic data for **4** in this article have been deposited at the Cambridge Crystallographic Data Centre under supplementary publication number 2342720. The data can be obtained via https://www.ccdc.cam.ac.uk/.

Table S3. X-ray diffraction analysis of chrysoquinazolinone A (**1**)

Empirical formula	$C_{21}H_{24}N_2O_4$
Formula weight	368.42
Temperature/K	293(2)
Crystal system	monoclinic
Space group	$P2_1$
a/Å	13.1209(5)
b/Å	21.3061(7)
c/Å	13.3607(5)
α/°	90
β/°	90.100(10)
γ/°	90
Volume/Å^3	3735.0(2)
Z	8
ρ_{calc}g/cm^3	1.310
μ/mm^{-1}	0.742
F(000)	1568
Crystal size/mm^3	0.200 × 0.200 × 0.100
Radiation	CuKα (λ = 1.54178)
2Θ range for data collection/°	
Index ranges	-16 ≤ h ≤ 16, -19 ≤ k ≤ 26, -16 ≤ l ≤ 16
Reflections collected	42099
Independent reflections	10888 [R_{int} = 0.0559, R_{sigma} = 0.0456]
Data/restraints/parameters	10888/1/991
Goodness-of-fit on F^2	1.028
Final R indexes [I>=2σ (I)]	R_1 = 0.0627, wR_2 = 0.1669
Final R indexes [all data]	R_1 = 0.0694, wR_2 = 0.1755
Largest diff. peak/hole / e Å^{-3}	0.34/-0.30
Flack parameter	-0.23(16)

Table S4. X-ray diffraction analysis of chrysobenzothiazole A (±**3**)

Empirical formula	$C_{13}H_{15}NO_3S$
Formula weight	265.32
Temperature/K	150
Crystal system	monoclinic
Space group	$P2_1/n$
a/Å	8.5937(3)
b/Å	9.1721(4)
c/Å	16.9210(7)
α/°	90
β/°	93.851(2)
γ/°	90
Volume/Å^3	1330.74(9)
Z	4
ρ_{calc}g/cm^3	1.324
μ/mm^{-1}	2.174
F(000)	560
Crystal size/mm^3	0.2 × 0.15 × 0.1
Radiation	CuKα (λ = 1.54178)
2Θ range for data collection/°	10.48 to 149.578
Index ranges	-10 ≤ h ≤ 10, -11 ≤ k ≤ 9, -21 ≤ l ≤ 21
Reflections collected	9467
Independent reflections	2724 [R_{int} = 0.0506, R_{sigma} = 0.0512]
Data/restraints/parameters	2724/0/167
Goodness-of-fit on F^2	1.109
Final R indexes [I>=2σ (I)]	R_1 = 0.0791, wR_2 = 0.1964
Final R indexes [all data]	R_1 = 0.0818, wR_2 = 0.2000
Largest diff. peak/hole / e Å^{-3}	0.65/-0.31

Table S5. X-ray diffraction analysis of chrysobenzothiazole B (**4**)

Empirical formula	$C_{13}H_{15}NO_3S$
Formula weight	265.32
Temperature/K	100.00(10)
Crystal system	orthorhombic
Space group	$P2_12_12_1$
a/Å	4.69830(10)
b/Å	15.3583(2)
c/Å	17.0647(3)
α/°	90
β/°	90
γ/°	90
Volume/Å^3	1231.35(4)
Z	4
ρ_{calc}g/cm^3	1.431
μ/mm^{-1}	2.35
F(000)	560
Crystal size/mm^3	0.2 × 0.15 × 0.1
Radiation	Cu Kα (λ = 1.54184)
2Θ range for data collection/°	7.744 to 151.38
Index ranges	$-5 \le h \le 5$, $-19 \le k \le 19$, $-20 \le l \le 21$
Reflections collected	5897
Independent reflections	2484 [R_{int} = 0.0295, R_{sigma} = 0.0372]
Data/restraints/parameters	2484/0/167
Goodness-of-fit on F^2	1.071
Final R indexes [I>=2σ (I)]	R_1 = 0.0318, wR_2 = 0.0844
Final R indexes [all data]	R_1 = 0.0335, wR_2 = 0.0855
Largest diff. peak/hole / e Å^{-3}	0.28/-0.22
Flack parameter	0.020(11)

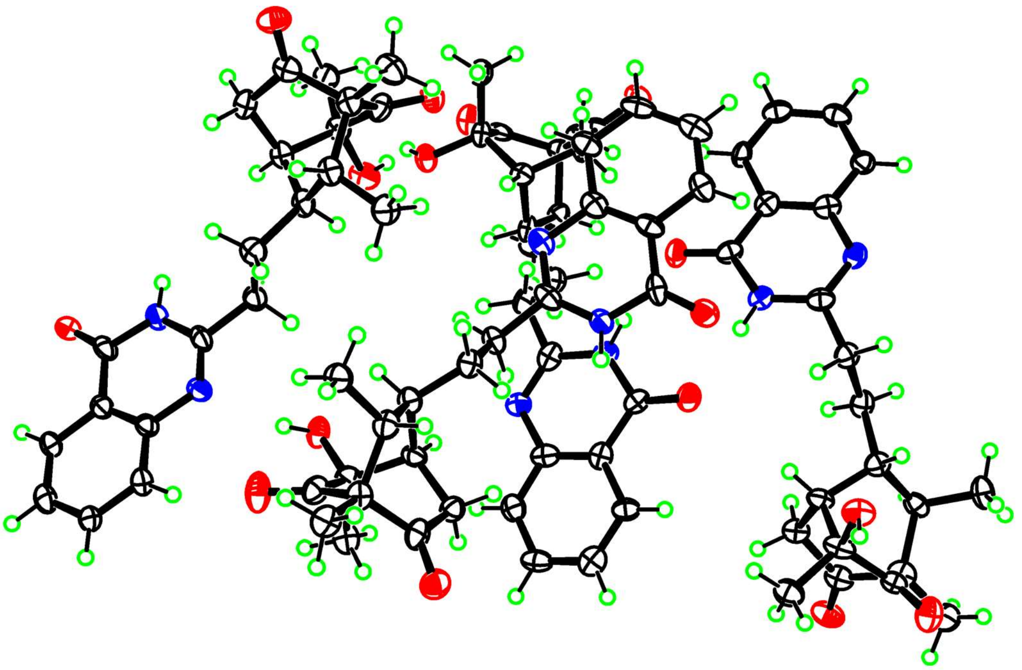

Figure S1. Original ORTEP drawing of **1** (displacement ellipsoids are drawn at the 50% probability level).

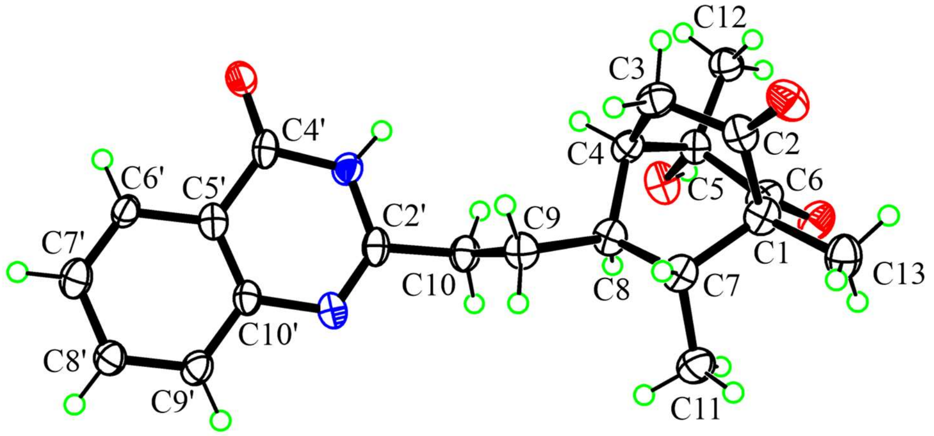

Figure S2. ORTEP drawing of **1** (displacement ellipsoids are drawn at the 50% probability level).

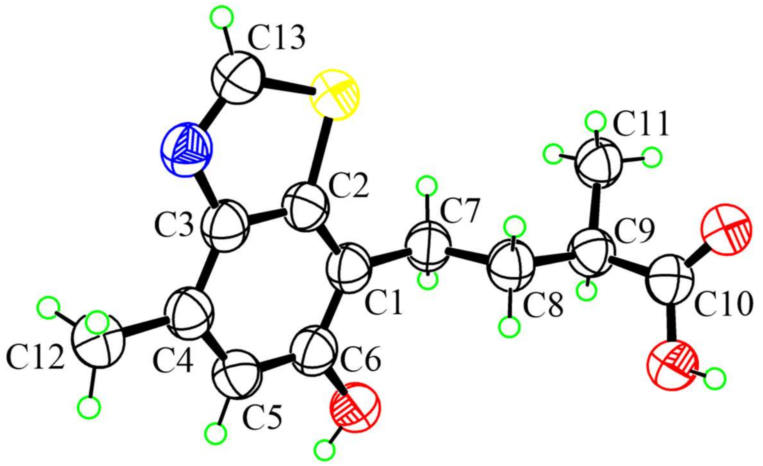

Figure S3. ORTEP drawing of **3** (displacement ellipsoids are drawn at the 50% probability level)

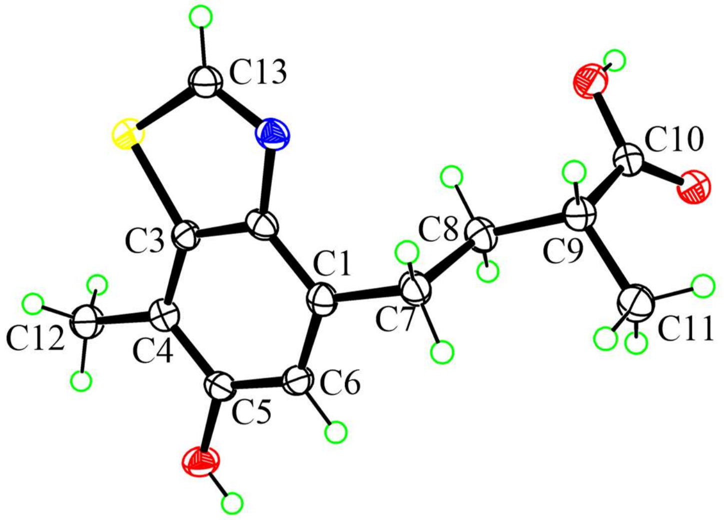

Figure S4. ORTEP drawing of **4** (displacement ellipsoids are drawn at the 50% probability level).

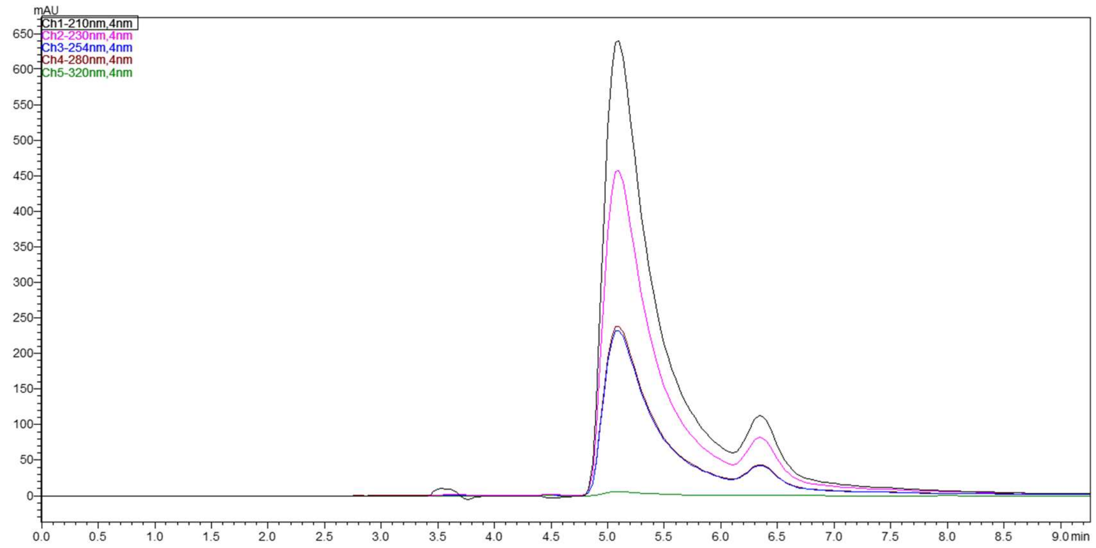

Figure S5. Chiral HPLC chromatogram of ±**3**.

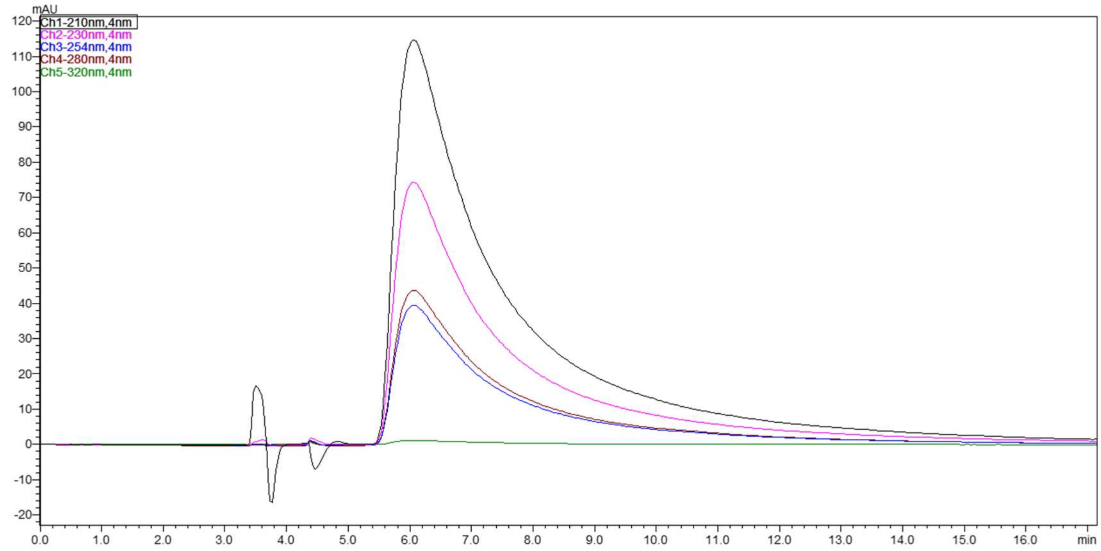

Figure S6. Chiral HPLC chromatogram of **4**.

3.Quantum chemical calculations

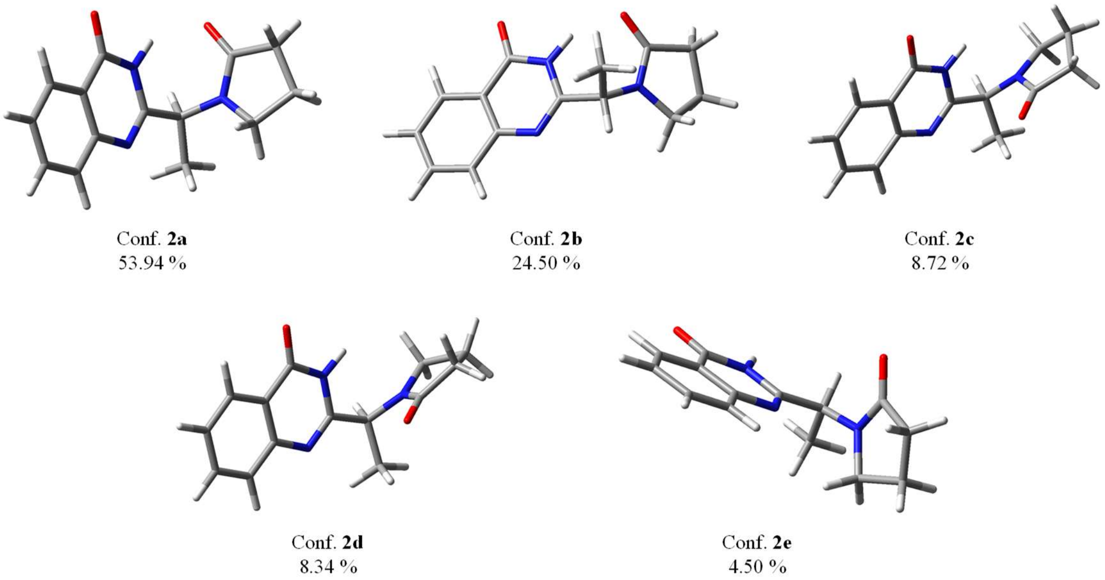

Figure S7. Stable conformers of compound **2**.

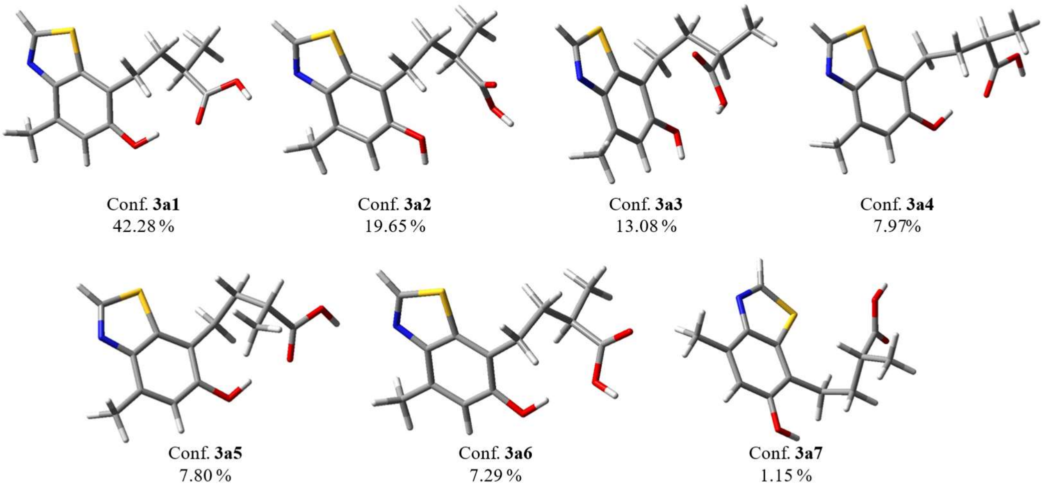

Figure S8. Stable conformers of compound **3**.

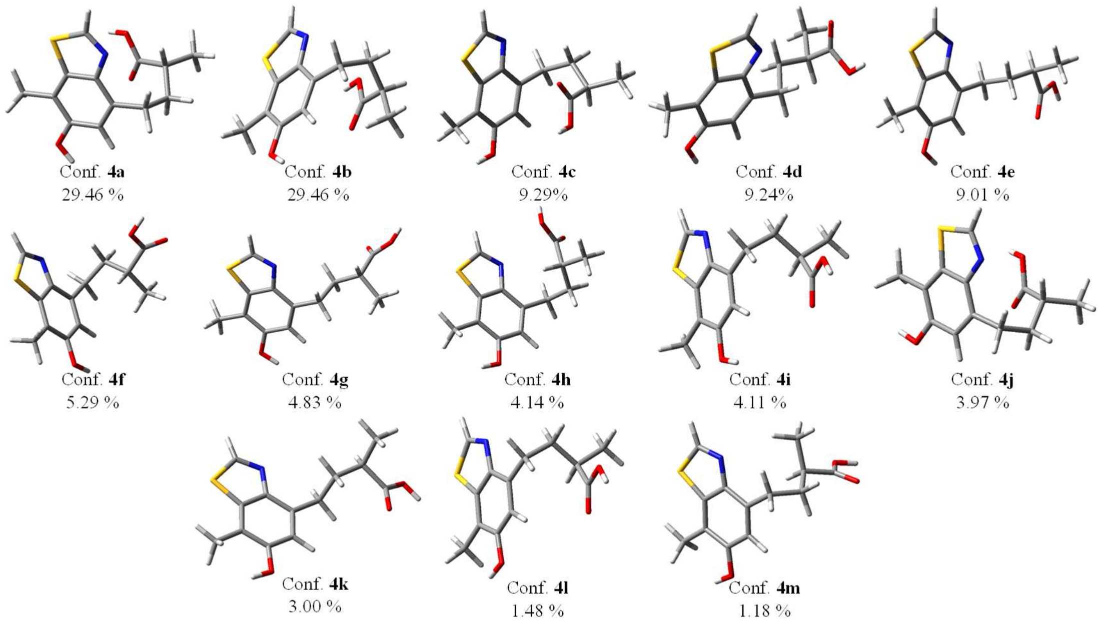

Figure S9. Stable conformers of compound **4**.

Table S6. Optimized Z-Matrixes of compounds **2**–**4** in the Gas Phase (Å) at B3LYP/6-31G (d, p) level.

Conf. **2a**				Conf. **2b**			
C	0.8269	7.3413	0.19228	C	-0.06576	7.92651	-0.65346
C	-0.14038	7.17621	1.20039	C	-1.10147	7.82654	0.29397
C	-1.08382	6.16367	1.11811	C	-1.64698	6.59479	0.62012
C	-1.08467	5.28466	0.01871	C	-1.16779	5.42235	0.0047
C	-0.10908	5.45644	-0.99338	C	-0.12505	5.52954	-0.94761
C	0.84107	6.48571	-0.89753	C	0.41802	6.78393	-1.26966
N	-2.04219	4.27829	-0.03015	N	-1.73902	4.20462	0.35237
C	-2.02365	3.48779	-1.05485	C	-1.2698	3.14273	-0.2277
N	-1.1136	3.59582	-2.08133	N	-0.25737	3.1625	-1.15175
C	-0.10825	4.54587	-2.13544	C	0.37312	4.31516	-1.58708
O	0.68325	4.57718	-3.08425	O	1.26895	4.26289	-2.43802
C	-3.00413	2.33249	-1.19765	C	-1.82428	1.78373	0.15683
N	-2.27539	1.06307	-1.09047	N	-2.32594	1.03527	-1.00648
C	-4.17264	2.41176	-0.22554	C	-0.83017	0.97105	0.99839
C	-1.63516	0.51781	-2.15423	C	-1.56273	0.40263	-1.92779
C	-0.84819	-0.68785	-1.68044	C	-2.45589	-0.49085	-2.76638
C	-1.36106	-0.93071	-0.25369	C	-3.86967	0.03531	-2.48082
C	-1.89557	0.44414	0.1863	C	-3.74642	0.66055	-1.08276
O	-1.65978	0.97951	-3.30713	O	-0.33157	0.52942	-2.05901
H	1.56006	8.13841	0.26985	H	0.35197	8.89813	-0.89926
H	-0.14805	7.84962	2.05276	H	-1.47789	8.72484	0.7752
H	-1.83411	6.02715	1.89076	H	-2.44661	6.50698	1.34924
H	1.57753	6.59478	-1.68725	H	1.21647	6.83659	-2.00299

H	-1.15886	2.91221	-2.84136	H	0.02214	2.26596	-1.5746
H	-3.38744	2.35881	-2.22313	H	-2.69998	1.99871	0.76975
H	-4.82427	1.544	-0.36537	H	-0.55668	1.54269	1.89088
H	-4.7518	3.31762	-0.42525	H	0.07765	0.73462	0.43975
H	-3.84021	2.44618	0.81434	H	-1.3029	0.03573	1.31434
H	-0.97895	-1.53212	-2.3624	H	-2.16103	-0.45762	-3.81794
H	0.2134	-0.40807	-1.69716	H	-2.31966	-1.52086	-2.4106
H	-0.59058	-1.3029	0.42491	H	-4.63672	-0.74127	-2.51556
H	-2.18331	-1.65288	-0.2699	H	-4.13104	0.8107	-3.2079
H	-2.75745	0.36819	0.85134	H	-4.37366	1.54611	-0.95017
H	-1.12672	1.04982	0.68333	H	-3.98246	-0.05406	-0.28359
Conf. **2c**				Conf. **2d**			
C	0.77617	7.5062	-0.18407	C	0.8012	7.46354	-0.15912
C	-0.0449	7.35948	0.94916	C	-0.00389	7.30724	0.98422
C	-0.93248	6.29946	1.04913	C	-0.90283	6.25638	1.08006
C	-1.02358	5.35133	0.01225	C	-1.02139	5.32754	0.02875
C	-0.19554	5.50567	-1.12613	C	-0.20944	5.4915	-1.11969
C	0.70008	6.58364	-1.21478	C	0.69774	6.56004	-1.20426
N	-1.91791	4.29806	0.14898	N	-1.92467	4.28147	0.16229
C	-1.97871	3.43642	-0.81456	C	-2.01158	3.4363	-0.81323
N	-1.22083	3.52861	-1.95621	N	-1.27138	3.54042	-1.96664
C	-0.2889	4.5237	-2.2016	C	-0.33323	4.5306	-2.21081
O	0.36847	4.52842	-3.24796	O	0.30389	4.54785	-3.26936
C	-2.97626	2.28954	-0.77627	C	-3.01026	2.28913	-0.77352
N	-2.38525	1.08585	-1.35905	N	-2.41596	1.07218	-1.32408
C	-3.55163	2.03782	0.61679	C	-3.60227	2.05632	0.61519
C	-1.23932	0.53006	-0.87726	C	-1.22359	0.57436	-0.90726
C	-1.07311	-0.83965	-1.51184	C	-0.90885	-0.65578	-1.74138
C	-2.0898	-0.84156	-2.66348	C	-2.25914	-1.02762	-2.37298
C	-3.15472	0.17211	-2.21038	C	-3.03069	0.30427	-2.41218
O	-0.49082	1.06806	-0.05469	O	-0.51655	1.08242	-0.02904
H	1.46695	8.34139	-0.24883	H	1.50133	8.29117	-0.22034
H	0.01796	8.08495	1.75536	H	0.08054	8.0178	1.80163
H	-1.56903	6.17636	1.91989	H	-1.52683	6.1256	1.95873
H	1.32368	6.67699	-2.09811	H	1.30862	6.66124	-2.09558
H	-1.30024	2.79596	-2.65703	H	-1.37518	2.83365	-2.69017
H	-3.79187	2.57376	-1.45252	H	-3.81779	2.56288	-1.463
H	-4.06461	2.9283	0.98735	H	-4.10855	2.95565	0.97373
H	-2.75916	1.77217	1.32109	H	-2.81982	1.78678	1.32897
H	-4.27193	1.21574	0.5636	H	-4.33002	1.2411	0.55976
H	-0.03716	-1.00524	-1.81831	H	-0.16536	-0.36458	-2.49514
H	-1.31912	-1.58719	-0.74605	H	-0.46726	-1.44144	-1.12338
H	-2.51969	-1.82604	-2.86036	H	-2.78759	-1.73511	-1.72607
H	-1.61316	-0.48401	-3.58206	H	-2.16519	-1.47475	-3.36497
H	-3.60892	0.72076	-3.03987	H	-2.90056	0.83341	-3.36549
H	-3.95576	-0.30342	-1.62887	H	-4.10245	0.18135	-2.23233

Conf. **2e**							
C	0.99358	6.911	0.49249				
C	1.02716	5.77561	1.32303				
C	0.32842	4.62773	0.98306				
C	-0.42751	4.58483	-0.20371				
C	-0.45669	5.73007	-1.03559				
C	0.25591	6.88676	-0.67995				
N	-1.11626	3.41994	-0.51283				
C	-1.8055	3.40119	-1.60827				
N	-1.87882	4.4701	-2.46771				
C	-1.23558	5.6842	-2.26837				
O	-1.34749	6.59754	-3.09195				
C	-2.59546	2.16407	-2.02281				
N	-2.27146	1.04006	-1.16471				
C	-4.10304	2.44138	-2.06451				
C	-1.07537	0.40256	-1.29789				
C	-0.86414	-0.47979	-0.08139				
C	-2.23966	-0.50808	0.59839				
C	-2.89758	0.80598	0.14234				
O	-0.29749	0.55514	-2.24613				
H	1.54574	7.80321	0.77148				
H	1.60738	5.79881	2.24117				
H	0.34779	3.74594	1.61604				
H	0.21595	7.75029	-1.33619				
H	-2.42298	4.38993	-3.32339				
H	-2.26032	1.89628	-3.03093				
H	-4.4729	2.79519	-1.09765				
H	-4.33	3.20208	-2.81703				
H	-4.63728	1.52667	-2.33755				
H	-0.47316	-1.45983	-0.36662				
H	-0.10904	0.01382	0.54493				
H	-2.18464	-0.57786	1.68708				
H	-2.82228	-1.35827	0.22891				
H	-3.98189	0.72026	0.04513				
H	-2.67018	1.63739	0.81971				
Conf. **3a1**				Conf. **3a2**			
C	0.64301	-0.07906	-0.03971	C	0.61531	-0.00528	0.00695
C	0.2092	-0.36605	-1.34325	C	0.13381	-0.16042	-1.29741
C	0.74701	-1.53454	-1.90808	C	0.59952	-1.29422	-1.97583
C	1.68051	-2.33052	-1.20899	C	1.49144	-2.20963	-1.38506
C	2.1135	-2.02528	0.07263	C	1.96615	-2.04107	-0.0894
C	1.57234	-0.86945	0.67186	C	1.51518	-0.90948	0.61794
N	1.8945	-0.41106	1.94517	N	1.89275	-0.58391	1.91719
C	1.25606	0.67995	2.21959	C	1.32175	0.50965	2.30484
S	0.17636	1.28963	0.96322	S	0.24603	1.28804	1.13991
C	3.11004	-2.88051	0.80614	C	2.92242	-3.01547	0.54188

O	0.44448	-1.97039	-3.16654	O	0.14403	-1.48147	-3.25419
C	-0.76146	0.55094	-2.05217	C	-0.85655	0.79156	-1.9202
C	-2.23044	0.35841	-1.62743	C	-2.31395	0.52568	-1.49034
C	-2.86029	-1.00369	-2.01274	C	-2.83033	-0.88534	-1.79254
C	-2.94489	-1.0732	-3.51842	C	-2.8612	-1.16993	-3.27873
O	-2.00493	-1.37435	-4.2508	O	-2.99192	-0.33424	-4.15832
C	-4.20612	-1.21155	-1.31522	C	-4.25495	-1.08998	-1.23895
O	-4.13131	-0.71786	-4.02003	O	-2.79156	-2.4893	-3.5464
H	2.05693	-3.21616	-1.71278	H	1.81438	-3.06941	-1.96757
H	1.34648	1.22204	3.15505	H	1.46502	0.95985	3.28129
H	4.01091	-2.30901	1.06071	H	3.86	-2.52233	0.82553
H	2.69394	-3.25334	1.74983	H	2.50129	-3.44675	1.45799
H	3.40901	-3.73984	0.19882	H	3.15884	-3.83264	-0.14575
H	-0.42461	-1.63453	-3.49024	H	0.5122	-2.30604	-3.61504
H	-0.48437	1.58932	-1.83062	H	-0.59843	1.82062	-1.64021
H	-0.66334	0.44519	-3.13667	H	-0.78174	0.73579	-3.00888
H	-2.30824	0.44972	-0.53813	H	-2.40959	0.68819	-0.41034
H	-2.83713	1.1651	-2.05681	H	-2.96296	1.26021	-1.98123
H	-2.1672	-1.79855	-1.7158	H	-2.17424	-1.62953	-1.3292
H	-4.6764	-2.15056	-1.62555	H	-4.61198	-2.10765	-1.42726
H	-4.05082	-1.24883	-0.23192	H	-4.25852	-0.91831	-0.15748
H	-4.89964	-0.39252	-1.53162	H	-4.95623	-0.38617	-1.70172
H	-4.0743	-0.74133	-4.99712	H	-2.86898	-2.60573	-4.5148
Conf. **3a3**				Conf. **3a4**			
C	0.0938	-0.58509	0.14219	C	0.5496	-0.72367	0.50372
C	-0.77411	-1.59636	-0.28683	C	-0.03312	-1.15458	-0.69316
C	-0.20732	-2.54559	-1.14666	C	0.86597	-1.56748	-1.6895
C	1.13888	-2.47773	-1.55578	C	2.25881	-1.56967	-1.47495
C	1.98684	-1.46325	-1.1301	C	2.8252	-1.15364	-0.27724
C	1.44607	-0.49744	-0.25755	C	1.94486	-0.71621	0.73274
N	2.15136	0.58219	0.26482	N	2.33908	-0.25981	1.98688
C	1.39471	1.30279	1.02716	C	1.31417	0.07446	2.70135
S	-0.27669	0.76227	1.20669	S	-0.26811	-0.12331	1.94178
C	3.42177	-1.38514	-1.57326	C	4.31291	-1.15858	-0.0536
O	-1.0238	-3.54761	-1.60025	O	0.45831	-2.00212	-2.91912
C	-2.23441	-1.62252	0.09121	C	-1.52079	-1.15104	-0.93395
C	-3.10639	-0.63578	-0.71281	C	-1.97236	0.02819	-1.82348
C	-3.08284	-0.85369	-2.23413	C	-3.3205	-0.20641	-2.50684
C	-1.78798	-0.3638	-2.85212	C	-3.22421	-1.34485	-3.50228
O	-1.24795	0.70056	-2.59621	O	-2.18976	-1.88125	-3.8829
C	-4.23015	-0.08778	-2.92187	C	-3.82347	1.05762	-3.23018
O	-1.31686	-1.19833	-3.79981	O	-4.4214	-1.71865	-3.97339
H	1.51461	-3.24428	-2.22966	H	2.894	-1.90933	-2.28807
H	1.72497	2.19689	1.5452	H	1.37481	0.45951	3.71393
H	3.62348	-0.4418	-2.09488	H	4.68355	-0.15129	0.17198
H	4.10388	-1.42557	-0.71552	H	4.58371	-1.79401	0.79829

H	3.66787	-2.21051	-2.24761	H	4.83969	-1.52803	-0.93832
H	-0.52564	-4.11738	-2.21093	H	-0.50364	-1.87605	-3.07158
H	-2.62609	-2.63476	-0.04222	H	-1.80925	-2.10318	-1.39459
H	-2.33832	-1.38351	1.15749	H	-2.05762	-1.11052	0.01983
H	-4.1414	-0.74015	-0.36751	H	-2.03635	0.93427	-1.21167
H	-2.80582	0.39717	-0.50227	H	-1.22202	0.22871	-2.59648
H	-3.18328	-1.91971	-2.45893	H	-4.07286	-0.50254	-1.76416
H	-5.19407	-0.45581	-2.55576	H	-4.79838	0.89086	-3.6971
H	-4.16208	0.98388	-2.70399	H	-3.92067	1.87631	-2.51015
H	-4.20169	-0.22105	-4.00897	H	-3.11459	1.36664	-4.00702
H	-0.50907	-0.79671	-4.17943	H	-4.28797	-2.43746	-4.62418
Conf. **3a5**				Conf. **3a6**			
C	0.26287	-0.73001	0.43441	C	0.65602	-0.1366	-0.03804
C	-0.34305	-1.40385	-0.63632	C	0.1933	-0.47066	-1.32103
C	0.54337	-1.91903	-1.59462	C	0.72992	-1.65202	-1.85641
C	1.94113	-1.78865	-1.45864	C	1.68227	-2.42056	-1.15346
C	2.52847	-1.1357	-0.38493	C	2.14016	-2.07096	0.10777
C	1.66082	-0.58594	0.58069	C	1.60616	-0.89735	0.6782
N	2.07372	0.09637	1.72048	N	1.95618	-0.3933	1.92661
C	1.05889	0.46547	2.43242	C	1.31928	0.70419	2.17749
S	-0.5339	0.03269	1.80551	S	0.20706	1.26469	0.92689
C	4.02068	-1.00772	-0.24575	C	3.15611	-2.89668	0.84817
O	0.12668	-2.58814	-2.71164	O	0.41027	-2.13244	-3.09713
C	-1.84212	-1.58717	-0.69504	C	-0.80343	0.41585	-2.03405
C	-2.67115	-0.35486	-1.11336	C	-2.25472	0.24941	-1.54035
C	-2.62031	0.09273	-2.59862	C	-2.92183	-1.10355	-1.88465
C	-3.04692	-1.0575	-3.47876	C	-3.18892	-1.14624	-3.37211
O	-2.3353	-2.00586	-3.79838	O	-4.15121	-0.65221	-3.93
C	-1.27359	0.66579	-3.05735	C	-4.23039	-1.28446	-1.11006
O	-4.32387	-0.97986	-3.86917	O	-2.20944	-1.77534	-4.06486
H	2.56194	-2.21823	-2.23958	H	2.05308	-3.31992	-1.63653
H	1.13509	1.01335	3.36555	H	1.4295	1.27959	3.09066
H	4.32601	0.04417	-0.19234	H	2.7624	-3.23739	1.81331
H	4.37504	-1.48783	0.6746	H	3.4447	-3.77583	0.26468
H	4.5326	-1.47226	-1.09355	H	4.06014	-2.31375	1.06222
H	-0.79542	-2.35128	-2.9673	H	-0.44862	-1.79252	-3.4162
H	-2.0912	-2.43948	-1.33368	H	-0.51234	1.4616	-1.87318
H	-2.183	-1.86803	0.31004	H	-0.74962	0.26196	-3.11677
H	-3.71843	-0.56491	-0.86594	H	-2.28031	0.35684	-0.45019
H	-2.3758	0.51187	-0.51106	H	-2.86936	1.059	-1.95214
H	-3.3804	0.87266	-2.70447	H	-2.22763	-1.9136	-1.63885
H	-0.49768	-0.09651	-3.12807	H	-4.7177	-2.23088	-1.36845
H	-1.37718	1.13047	-4.04437	H	-4.0264	-1.28531	-0.0345
H	-0.93875	1.43347	-2.35232	H	-4.92869	-0.46974	-1.32822
H	-4.53835	-1.77891	-4.39355	H	-2.40452	-1.71152	-5.02283
Conf. **3a7**							

C	0.30306	-1.68529	-0.65499				
C	0.51787	-1.3388	-1.99599				
C	1.60151	-0.47798	-2.222				
C	2.40373	0.01116	-1.174				
C	2.17345	-0.33138	0.15147				
C	1.09879	-1.20545	0.41108				
N	0.73678	-1.65956	1.67519				
C	-0.2831	-2.45221	1.60709				
S	-0.93749	-2.74997	-0.00389				
C	3.02856	0.19388	1.27181				
O	1.94921	-0.04831	-3.47776				
C	-0.38445	-1.81068	-3.11236				
C	-1.35581	-0.72557	-3.62378				
C	-2.29516	-0.1431	-2.5631				
C	-3.27065	-1.17784	-2.04245				
O	-3.55882	-2.22753	-2.59428				
C	-3.09857	1.04843	-3.12235				
O	-3.84292	-0.79542	-0.88396				
H	3.22468	0.67384	-1.43188				
H	-0.74276	-2.92995	2.46588				
H	2.42941	0.76225	1.99345				
H	3.50123	-0.62506	1.82726				
H	3.81619	0.84866	0.88786				
H	1.43544	-0.52137	-4.15322				
H	0.2164	-2.16623	-3.96074				
H	-0.95571	-2.68221	-2.77835				
H	-0.77776	0.10549	-4.04321				
H	-1.9513	-1.1461	-4.44196				
H	-1.71329	0.21322	-1.70528				
H	-3.75268	1.4809	-2.35928				
H	-2.40961	1.82632	-3.46729				
H	-3.71628	0.73547	-3.97205				
H	-4.48555	-1.4846	-0.62033				
Conf. **4a**				Conf. **4b**			
C	1.41117	-0.39707	0.30405	C	1.0462	0.1898	-1.03162
C	1.20288	0.97388	0.53558	C	-0.2565	-0.21745	-1.37545
C	1.22133	1.8193	-0.5704	C	-0.6336	-1.50853	-1.01981
C	1.40595	1.32902	-1.87172	C	0.22341	-2.36038	-0.3042
C	1.57831	-0.03829	-2.13996	C	1.50733	-1.9653	0.09678
C	1.57934	-0.87294	-1.02162	C	1.88696	-0.6783	-0.29278
S	1.74921	-2.62397	-0.9841	S	3.40898	0.14162	0.03631
C	1.58859	-2.55401	0.77323	C	2.78828	1.54288	-0.84104
N	1.43057	-1.38082	1.29009	N	1.5964	1.43392	-1.32836
C	1.7471	-0.52567	-3.55244	C	2.38218	-2.89573	0.89055
C	0.85298	1.49406	1.90638	C	-1.23152	0.7348	-2.02195
C	-0.669	1.63898	2.11925	C	-1.61229	1.93162	-1.12499

C	-1.47105	0.33582	1.97688	C	-2.17958	1.58412	0.27353
C	-1.67041	-0.0682	0.52849	C	-1.12065	1.06297	1.22974
O	-1.89274	0.69796	-0.39502	O	-1.16697	0.01046	1.8451
C	-2.87762	0.48123	2.59464	C	-3.38886	0.65061	0.22826
O	-1.67217	-1.40684	0.36447	O	-0.10414	1.93661	1.39097
O	1.41613	2.16457	-2.95683	O	-0.16253	-3.62464	0.05464
H	1.0603	2.88616	-0.43164	H	-1.62973	-1.86389	-1.27154
H	1.63536	-3.47241	1.34907	H	3.41299	2.42327	-0.94883
H	2.64889	-0.10297	-4.01234	H	2.58928	-3.81702	0.33241
H	1.82747	-1.61561	-3.59707	H	3.34088	-2.43352	1.14272
H	0.89772	-0.22452	-4.17719	H	1.89395	-3.19141	1.82701
H	1.25852	0.82366	2.67022	H	-0.8032	1.14116	-2.94644
H	1.31393	2.47761	2.05294	H	-2.13196	0.18043	-2.30537
H	-0.83297	2.02848	3.13077	H	-0.74161	2.581	-1.00356
H	-1.07263	2.38243	1.42185	H	-2.37656	2.51647	-1.65015
H	-0.94768	-0.48121	2.48255	H	-2.49086	2.54251	0.71239
H	-2.79177	0.70099	3.66377	H	-4.14625	1.0556	-0.45148
H	-3.42611	1.30023	2.1157	H	-3.11501	-0.34886	-0.1202
H	-3.46044	-0.43887	2.4779	H	-3.84081	0.54657	1.21996
H	-1.86014	-1.59452	-0.57721	H	0.53747	1.53746	2.01157
H	1.27712	3.08076	-2.66026	H	-1.06774	-3.78914	-0.2617
Conf. **4c**				Conf. **4d**			
C	0.14554	-0.5235	-1.35455	C	0.91321	-0.70581	-0.6308
C	0.17825	0.86924	-1.14387	C	0.96924	0.5522	-0.00247
C	1.38505	1.42241	-0.7368	C	1.73303	1.53822	-0.61846
C	2.52582	0.63143	-0.51127	C	2.42808	1.29503	-1.81581
C	2.52184	-0.75701	-0.69752	C	2.39565	0.05186	-2.46311
C	1.3038	-1.30103	-1.12339	C	1.62006	-0.92832	-1.83795
S	0.92938	-2.99131	-1.43477	S	1.34603	-2.58607	-2.36297
C	-0.71958	-2.50354	-1.83444	C	0.33087	-2.81961	-0.93632
N	-0.97857	-1.23925	-1.75358	N	0.19774	-1.80033	-0.15261
C	3.75657	-1.58006	-0.44166	C	3.15471	-0.16732	-3.74291
C	-1.07171	1.70336	-1.26274	C	0.19831	0.81937	1.26469
C	-2.0395	1.51987	-0.07606	C	-1.2905	1.09304	0.98751
C	-1.45311	1.89702	1.29368	C	-2.11242	1.37808	2.26123
C	-0.47944	0.85356	1.80506	C	-2.07514	0.15422	3.15123
O	-0.66079	-0.35272	1.76648	O	-2.64723	-0.89603	2.90831
C	-2.56504	2.03222	2.35334	C	-3.56657	1.72512	1.9185
O	0.60038	1.39812	2.40004	O	-1.31514	0.32378	4.25013
O	3.64456	1.30089	-0.08977	O	3.17957	2.27307	-2.41217
H	1.46181	2.48965	-0.55229	H	1.80264	2.52549	-0.16678
H	-1.44221	-3.25694	-2.12976	H	-0.13576	-3.78413	-0.76646
H	3.59631	-2.63394	-0.68399	H	2.73727	0.43448	-4.56019
H	4.60369	-1.23241	-1.04788	H	4.20482	0.1268	-3.63249
H	4.06211	-1.52945	0.61201	H	3.13012	-1.21606	-4.05282
H	-0.79108	2.76027	-1.33689	H	0.29355	-0.04418	1.93274

H	-1.60693	1.44268	-2.18269	H	0.63466	1.68061	1.78337
H	-2.91808	2.15119	-0.25318	H	-1.38286	1.95897	0.3216
H	-2.39126	0.48313	-0.04071	H	-1.73134	0.23463	0.46738
H	-0.91858	2.84986	1.21963	H	-1.65063	2.20818	2.80546
H	-2.14992	2.28055	3.33636	H	-4.14805	1.93904	2.82228
H	-3.26022	2.82659	2.06294	H	-3.59788	2.60999	1.27429
H	-3.12612	1.09532	2.44244	H	-4.04791	0.8956	1.3899
H	1.1585	0.66802	2.73663	H	-1.31877	-0.51607	4.75383
H	4.37238	0.67461	0.06272	H	3.1493	3.07803	-1.86663
Conf. **4e**				Conf. **4f**			
C	0.70299	0.2805	-1.29729	C	0.37789	0.04114	-1.51658
C	0.77269	0.09937	0.09642	C	0.53823	1.14394	-0.65463
C	1.89808	-0.5463	0.59895	C	1.80634	1.35441	-0.12469
C	2.92691	-1.00257	-0.24246	C	2.89399	0.51074	-0.42431
C	2.8869	-0.83674	-1.63416	C	2.76389	-0.59248	-1.27799
C	1.75307	-0.18529	-2.12663	C	1.48297	-0.79294	-1.80369
S	1.36869	0.19327	-3.80225	S	0.97224	-2.07132	-2.89596
C	-0.13835	0.92416	-3.2415	C	-0.65216	-1.37619	-2.87031
N	-0.34856	0.90156	-1.96624	N	-0.81254	-0.32037	-2.14095
C	4.01069	-1.33858	-2.49862	C	3.91073	-1.50853	-1.60659
C	-0.35116	0.54068	0.99834	C	-0.6288	2.02828	-0.29408
C	-1.50446	-0.47814	1.01987	C	-1.63497	1.34434	0.65234
C	-2.66368	-0.06409	1.93142	C	-1.0189	0.87982	1.99457
C	-2.23837	-0.02601	3.3845	C	-2.07737	0.10457	2.74596
O	-1.36237	-0.71313	3.88454	O	-2.74738	0.53131	3.67217
C	-3.85494	-1.03559	1.81054	C	-0.48711	2.03495	2.8428
O	-2.98055	0.83319	4.11062	O	-2.24025	-1.14203	2.25634
O	4.0313	-1.63616	0.26349	O	4.1303	0.74352	0.11856
H	1.99155	-0.70764	1.6708	H	1.97132	2.19507	0.54517
H	-0.82055	1.35925	-3.96422	H	-1.44145	-1.84139	-3.45133
H	4.16544	-2.41525	-2.35899	H	3.75493	-2.50674	-1.17588
H	3.81448	-1.16245	-3.56016	H	4.01297	-1.63589	-2.69172
H	4.95643	-0.84383	-2.24517	H	4.85566	-1.12058	-1.22161
H	0.03703	0.67623	2.01338	H	-0.2559	2.95267	0.15732
H	-0.73874	1.50882	0.66143	H	-1.17053	2.31154	-1.20387
H	-1.89401	-0.60163	0.00327	H	-2.45432	2.04341	0.8623
H	-1.1239	-1.45508	1.34029	H	-2.06789	0.48011	0.13886
H	-3.00677	0.94159	1.66253	H	-0.20776	0.17776	1.76971
H	-4.68599	-0.72882	2.45395	H	0.34134	2.53539	2.33405
H	-4.2096	-1.05644	0.77493	H	-1.27191	2.77554	3.03191
H	-3.55472	-2.05113	2.09291	H	-0.11977	1.67472	3.80972
H	-2.69631	0.76745	5.04498	H	-2.96536	-1.57109	2.75378
H	3.94907	-1.70138	1.23074	H	4.09463	1.54628	0.66642
Conf. **4g**				Conf. **4h**			
C	0.85516	-1.27561	-0.21077	C	0.31845	-0.52503	-0.82356
C	1.21024	-0.071	0.42614	C	0.27187	0.86958	-1.01055

C	2.39589	0.53357	0.02438	C	1.46943	1.51034	-1.30675
C	3.21575	-0.02059	-0.9769	C	2.68098	0.80615	-1.41169
C	2.88399	-1.21323	-1.63403	C	2.7618	-0.5811	-1.21637
C	1.69312	-1.81498	-1.21438	C	1.55087	-1.21553	-0.92136
S	0.98842	-3.31222	-1.80881	S	1.27495	-2.92471	-0.60096
C	-0.34891	-3.07333	-0.67868	C	-0.43772	-2.55725	-0.37222
N	-0.29181	-2.01557	0.0628	N	-0.78139	-1.3206	-0.51809
C	3.75642	-1.81581	-2.70074	C	4.07729	-1.3058	-1.32334
C	0.31561	0.55429	1.46662	C	-1.0131	1.63948	-0.83223
C	-0.86237	1.30858	0.82567	C	-1.17511	2.21755	0.58933
C	-1.8519	1.87018	1.85475	C	-1.16458	1.18866	1.72569
C	-3.05983	2.45824	1.15633	C	-2.33043	0.22721	1.63479
O	-3.11006	2.79819	-0.01415	O	-3.38455	0.43985	1.05813
C	-1.23674	2.95066	2.76677	C	-1.22306	1.88095	3.10291
O	-4.10982	2.60282	1.99082	O	-2.11433	-0.90131	2.34004
O	4.3846	0.59296	-1.34602	O	3.78762	1.55649	-1.71224
H	2.7065	1.46411	0.49459	H	1.48925	2.58527	-1.46329
H	-1.15222	-3.80154	-0.64033	H	-1.12832	-3.3577	-0.12889
H	4.36603	-2.63716	-2.30025	H	4.81229	-0.91145	-0.60893
H	3.15098	-2.22729	-3.51709	H	3.9674	-2.37423	-1.11968
H	4.43631	-1.07392	-3.12557	H	4.50952	-1.20789	-2.32823
H	-0.07814	-0.22476	2.13021	H	-1.03457	2.47455	-1.54144
H	0.90988	1.23757	2.08121	H	-1.86514	0.99439	-1.06208
H	-0.47852	2.12622	0.20384	H	-0.36214	2.92881	0.77776
H	-1.39605	0.62543	0.15552	H	-2.11298	2.78424	0.631
H	-2.21935	1.05509	2.49107	H	-0.24715	0.59251	1.68467
H	-1.98081	3.34583	3.46513	H	-1.18172	1.15058	3.91707
H	-0.41368	2.53169	3.35235	H	-0.37353	2.56365	3.20951
H	-0.84636	3.78263	2.16895	H	-2.14611	2.46302	3.20685
H	-4.83922	3.02109	1.49019	H	-2.91874	-1.45423	2.27787
H	4.47982	1.42524	-0.85161	H	4.57375	0.98731	-1.76569
Conf. **4i**					Conf. **4j**		
C	0.86218	0.96997	-1.02599	C	0.77305	-1.04706	0.45105
C	-0.24571	0.11047	-0.89381	C	1.41308	0.04363	1.06879
C	-0.02823	-1.24421	-1.12068	C	2.36578	0.72987	0.3245
C	1.24077	-1.74914	-1.45931	C	2.66241	0.37971	-1.00175
C	2.36379	-0.92086	-1.58655	C	2.01736	-0.68044	-1.65888
C	2.12889	0.44044	-1.3685	C	1.07628	-1.37484	-0.89392
S	3.29516	1.75346	-1.46091	S	0.07135	-2.72974	-1.39333
C	1.99711	2.87147	-1.02967	C	-0.64308	-2.73401	0.22128
N	0.82906	2.3496	-0.84165	N	-0.19932	-1.84579	1.04771
C	3.71727	-1.46414	-1.9535	C	2.33921	-1.02038	-3.09012
C	-1.59882	0.63927	-0.48541	C	0.99782	0.52401	2.43593
C	-1.67713	1.03833	1.0011	C	-0.02694	1.67686	2.37791
C	-1.37177	-0.10542	1.98487	C	-1.346	1.33838	1.66565
C	-2.27587	-1.29684	1.75255	C	-1.20592	1.33192	0.15534

O	-1.89735	-2.43443	1.5232	O	-0.53394	2.11524	-0.49582
C	-1.49043	0.36786	3.44321	C	-2.43932	2.37314	2.00523
O	-3.58418	-0.98247	1.83557	O	-1.99355	0.4063	-0.42945
O	1.42581	-3.08844	-1.68213	O	3.6121	1.14319	-1.62764
H	-0.85542	-1.94209	-1.02242	H	2.88429	1.58169	0.7554
H	2.21284	3.93097	-0.94154	H	-1.40474	-3.46734	0.46477
H	3.82476	-2.50506	-1.63762	H	3.39271	-1.3068	-3.20834
H	3.88135	-1.42922	-3.03909	H	1.73322	-1.85508	-3.45244
H	4.51731	-0.88262	-1.48196	H	2.15229	-0.16815	-3.75674
H	-1.84564	1.52142	-1.08694	H	0.5697	-0.30668	3.00521
H	-2.36101	-0.11836	-0.70214	H	1.87964	0.87785	2.98216
H	-0.96491	1.84706	1.19687	H	-0.26408	1.96991	3.40732
H	-2.67864	1.4337	1.20664	H	0.42423	2.55072	1.8934
H	-0.35606	-0.47174	1.81074	H	-1.69241	0.34715	1.97325
H	-1.27241	-0.44548	4.14433	H	-3.37591	2.14826	1.48355
H	-0.77452	1.17647	3.62541	H	-2.63487	2.36538	3.08245
H	-2.4963	0.74545	3.65508	H	-2.1182	3.38102	1.71882
H	-4.10168	-1.79922	1.68533	H	-1.89517	0.49664	-1.39871
H	0.59059	-3.55802	-1.51406	H	3.75028	0.82936	-2.53717
Conf. **4k**				Conf. **4l**			
C	1.09005	-1.05033	-0.7597	C	0.87719	0.88566	-0.89103
C	0.45919	0.20692	-0.68323	C	-0.20855	-0.00304	-0.75858
C	1.18431	1.30981	-1.11725	C	0.04777	-1.35303	-0.96362
C	2.494	1.18764	-1.61379	C	1.33216	-1.82493	-1.28858
C	3.15012	-0.04855	-1.70044	C	2.43347	-0.96769	-1.42192
C	2.40904	-1.1518	-1.26286	C	2.16229	0.38989	-1.21617
S	2.90461	-2.8403	-1.23097	S	3.28902	1.73859	-1.30818
C	1.32349	-3.24249	-0.55517	C	1.95332	2.82118	-0.90759
N	0.51061	-2.2545	-0.36953	N	0.79996	2.26582	-0.72573
C	4.55467	-0.14761	-2.23388	C	3.79901	-1.49766	-1.77179
C	-0.92741	0.3479	-0.10973	C	-1.58111	0.49553	-0.37729
C	-0.91891	0.33816	1.42917	C	-1.687	0.93898	1.09463
C	-2.3155	0.47024	2.04426	C	-1.35382	-0.16193	2.11735
C	-2.93245	1.81773	1.73237	C	-2.21607	-1.38932	1.91484
O	-2.31444	2.85291	1.54305	O	-1.79879	-2.5199	1.72229
C	-2.27776	0.30832	3.57719	C	-1.50303	0.35378	3.55831
O	-4.27954	1.77473	1.73411	O	-3.53485	-1.1153	1.97943
O	3.09662	2.35214	-2.01189	O	1.44245	-3.17874	-1.46858
H	0.74268	2.30162	-1.07976	H	-0.75168	-2.0805	-0.8656
H	1.08933	-4.27657	-0.32549	H	2.1359	3.88781	-0.83072
H	5.25308	0.45888	-1.64214	H	4.12049	-2.27533	-1.06622
H	4.9224	-1.17699	-2.21407	H	3.81398	-1.93853	-2.77748
H	4.61601	0.20016	-3.27384	H	4.55586	-0.709	-1.7499
H	-1.36902	1.2837	-0.46814	H	-1.8499	1.3499	-1.00898
H	-1.56039	-0.47304	-0.46583	H	-2.31671	-0.29236	-0.57693
H	-0.47689	-0.6008	1.78035	H	-1.00394	1.77675	1.27051

H	-0.28314	1.15195	1.79714	H	-2.70297	1.30711	1.27865
H	-2.97773	-0.29869	1.63001	H	-0.32491	-0.49976	1.96406
H	-3.27958	0.39057	4.01119	H	-1.26253	-0.42785	4.28756
H	-1.87011	-0.67491	3.83369	H	-0.8187	1.19354	3.71988
H	-1.63966	1.07537	4.03066	H	-2.52388	0.70113	3.7492
H	-4.61329	2.68272	1.58497	H	-4.02503	-1.95213	1.84978
H	3.98361	2.16691	-2.36398	H	2.35741	-3.41537	-1.6954
Conf. **4m**							
C	0.64448	-1.2254	-0.00468				
C	0.97251	-0.06733	0.72572				
C	1.89888	0.80075	0.15714				
C	2.48768	0.54614	-1.09175				
C	2.18366	-0.59944	-1.84349				
C	1.25126	-1.46433	-1.26337				
S	0.63276	-2.97529	-1.92142				
C	-0.35979	-3.14533	-0.47146				
N	-0.25794	-2.20303	0.40693				
C	2.82956	-0.84908	-3.18076				
C	0.32197	0.24966	2.05167				
C	-0.65505	1.43781	1.97336				
C	-1.81744	1.23261	0.99268				
C	-2.62337	2.50593	0.86075				
O	-2.64676	3.42119	1.66747				
C	-2.7679	0.09177	1.40893				
O	-3.36877	2.5238	-0.26362				
O	3.38192	1.48404	-1.53693				
H	2.1878	1.70846	0.67944				
H	-1.00482	-4.01114	-0.36462				
H	3.92504	-0.83642	-3.10699				
H	2.54576	-1.82229	-3.5901				
H	2.53988	-0.08667	-3.91625				
H	-0.18986	-0.63861	2.42968				
H	1.10012	0.50472	2.78114				
H	-1.0592	1.63038	2.97424				
H	-0.09835	2.33604	1.68089				
H	-1.42427	1.00187	-0.00451				
H	-3.6062	0.00602	0.7102				
H	-2.23283	-0.86118	1.41287				
H	-3.17103	0.27362	2.41221				
H	-3.89442	3.34916	-0.26435				
H	3.72759	1.2301	-2.40911				

4. Optimization of culture conditions

The optimal culture condition was determined by the orthogonal experiment, in which three media, two culture methods and nine culture periods were used.

The fungal strain 19-7-ZM-4 was initially cultured on the panels of PDA medium at 28 °C for 3 days. The fresh mycelia were inoculated into 500 mL Erlenmeyer flasks which containing 150 mL of the seed medium (medium 1) and then were grown on a rotary shaker (180 rpm) at 28 °C for 24 hours. Subsequently, 1.0 mL seed culture was inoculated into the 500 mL Erlenmeyer flask containing 150 mL of different liquid culture media. The flasks were subjected to the rotary shakers for 180 rpm shake culture or static culture at 28 °C. The fermented whole broth was filtered under reduced pressure to separate the supernatant from mycelia. The supernatant was extracted three times with EtOAc and all the extracts were evaporated under reduced pressure to yield residue. Each residue was weighed and subjected to HPLC and TLC analysis. Integration of all factors, the fungus was grown on a rotary shaker (180 rpm) in medium 3 at 28 °C for 13 days.

Table S7. Ingredient of media **1** – **3**

Media	Ingredient
Medium 1	dextrose 20 g/L and potato 200 g/L and seawater
Medium 2	mannitol (20.0 g/L), maltose (20 g/L), dextrose (10 g/L), sodium glutamate (10 g/L), KH_2PO4 (0.5 g/L), $MgSO4{\cdot}7H_2O$ (0.3 g/L), yeast extract powder (3 g/L), corn steep liquor (1 g/L) and seawater
Medium 3	dextrose (20 g/L), maltose (20 g/L), yeast extract powder (5 g/L), peptone (10 g/L), corn steep liquor (10 g/L), KH2PO4 (0.5 g/L), MgSO4 (0.5 g/L) and seawater.

Table S8. HPLC chromatograms of residue

Culture method	Period	Medium 1	Medium 2	Medium 3
Shake culture	7 d	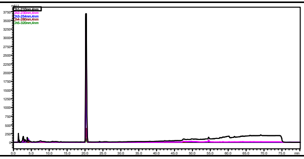	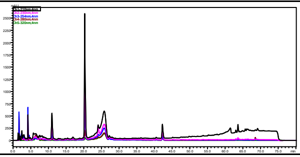	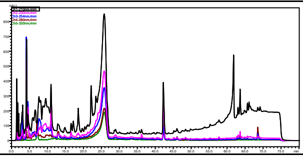
	9 d	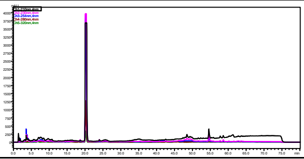	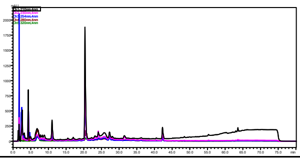	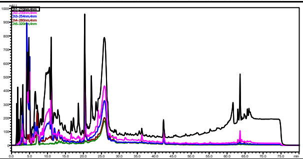
	11 d	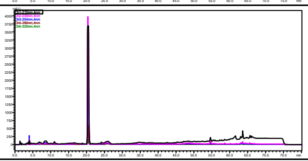	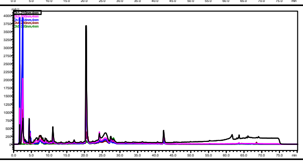	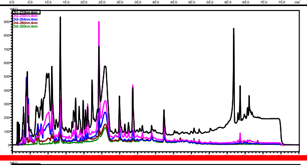
	13 d	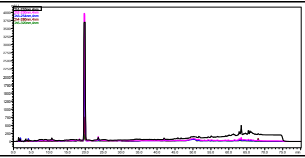	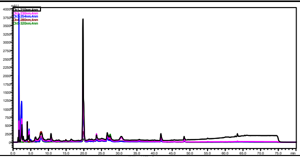	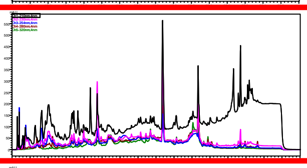
Static culture	15 d	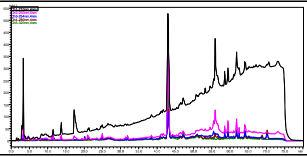	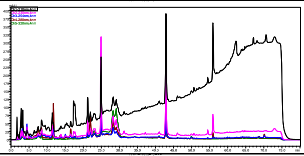	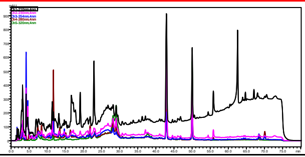
	20 d	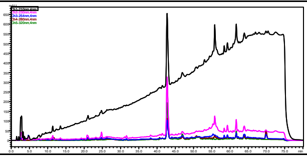	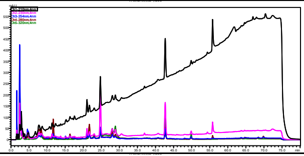	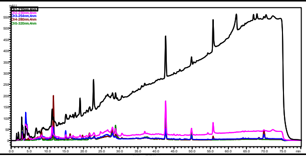
	25 d	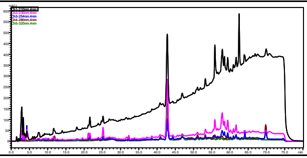	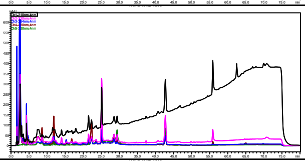	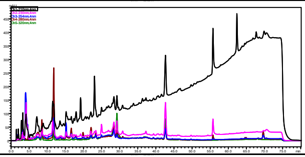
	30 d	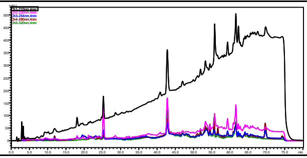	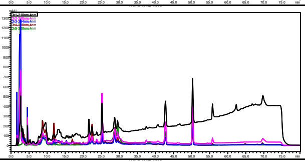	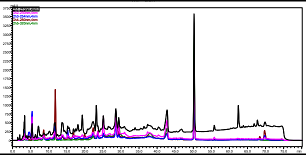
	35 d	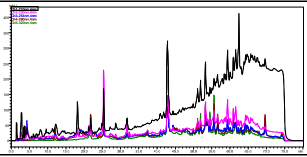	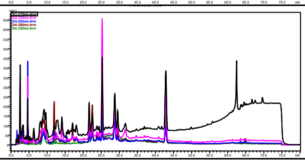	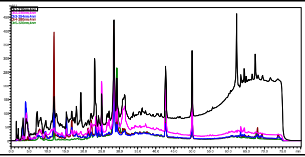

Table S9. TLC chromatograms of residue

Culture method	Shake culture				Static culture				
Period (d)	7	9	11	13	15	20	25	30	35
Medium 1	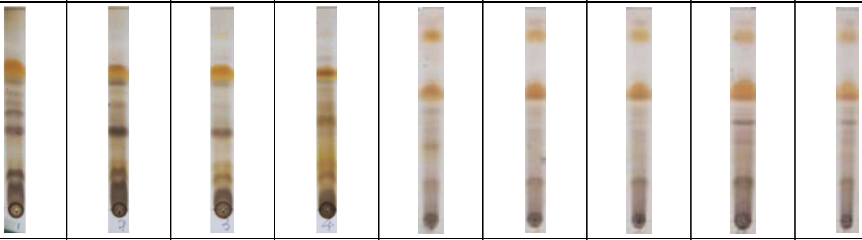								
Medium 2	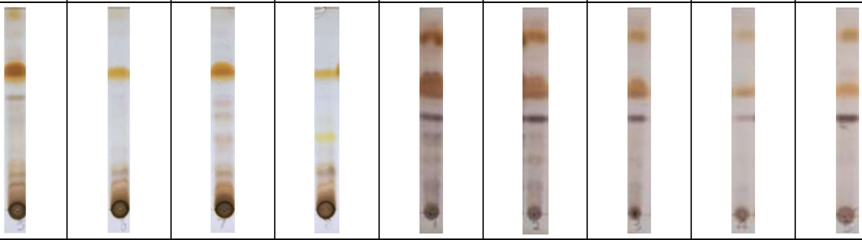								
Medium 3	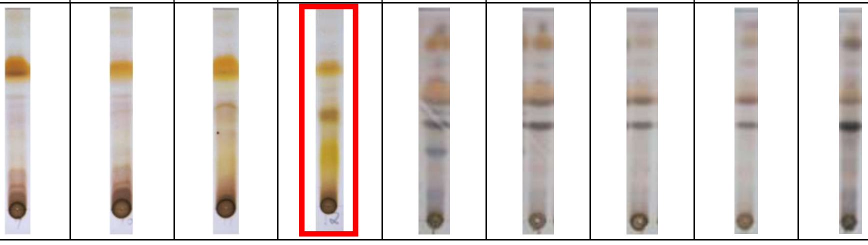								

Table S10. Mass of residue

Culture method	Periods	Medium 1	Medium 2	Medium 3
Shake culture	7 d	0.46	0.52	0.89
	9 d	0.47	0.51	0.9
	11 d	0.48	0.66	0.6
	13 d	0.6	0.66	0.79
Static culture	15 d	0.36	0.33	0.78
	20 d	0.4	0.33	0.69
	25 d	0.42	0.31	0.55
	30 d	0.36	0.32	0.66
	35 d	0.45	0.44	0.64

5. Cytotoxicity Assay.

The cytotoxic activity against the K562 (human leukemia) cell line was evaluated by MTT method, and the cytotoxic activities against L-02 (normal human hepato-cytes), ASPC-1 (human pancreatic cancer), MDA-MB-231 (human breast cancer), NCI-H446 and NCI-H446/EP (human small cell lung cancer) cell lines were determined by SRB method. Doxorubicin was used as a positive control.

In the cytotoxic assay, preliminary screening for cytotoxicity of compounds was performed at the concentration of 30 μM, then the compounds with inhibition rate greater than 60 % were rescreened for the IC_{50} values. The blank control, vehicle control, positive control (doxorubicin) and compounds at five different concentrations (30, 15, 3.75, 1.875 and 0.9375 μM) were tested three times and the average OD values were taken to calculated IC50 values.

Table S11. Cytotoxic activities of **1**–**5**

Compound	*c* (μM)	Inhibition rate (%)					
		K562	ASPC-1	L-02	MDA-MB-231	NCI-H446	NCI-H446/EP
**1**	30	35.08	0.00	-0.42	-4.59	2.22	9.68
**2**	30	33.57	7.93	0.28	5.80	4.70	12.47
**3a**	30	20.39	18.16	10.94	0.40	1.04	11.04
**3b**	30	34.54	2.09	-5.91	-4.16	9.14	14.64
**4**	30	37.63	8.56	-1.81	6.32	6.55	7.13
**5**	30	97.15	25.47	13.89	-14.63	86.08	54.99
Doxorubicin[a]	1	100.00	29.65	87.67	65.37	52.20	27.60

[a] Doxorubicin was used as positive control.

Table S12. Cytotoxicity against K562 cell line of **5**

	c (μM)	OD value	Inhibition rate (%)	IC_{50} (μM)
Blank control		1.10±0.03		
Vehicle control		1.09±0.02		
doxorubicin	1	0.36±0.01	100.00	
**5**	30	0.54±0.01	77.22	15.00
	15	0.76±0.03	47.36	
	7.5	0.95±0.04	20.85	
	3.75	1.00±0.03	13.35	
	1.875	1.04±0.03	7.71	
	0.9375	1.03±0.02	8.60	

Table S13. Cytotoxicity against NCI-H446 cell line of **5**

	c (μM)	OD value	Inhibition rate (%)	IC_{50} (μM)
Blank control		1.39±0.04		
Vehicle control		1.44±0.01		
doxorubicin	1	0.65±0.02	82.33	
**5**	30	0.36±0.02	74.28	16.87
	15	0.68±0.04	51.20	
	7.5	1.20±0.02	14.10	
	3.75	1.36±0.01	2.66	
	1.875	1.39±0.01	0.43	
	0.9375	1.50±0.06	-8.00	

6.The HRESIMS, UV and NMR spectra of 1–5

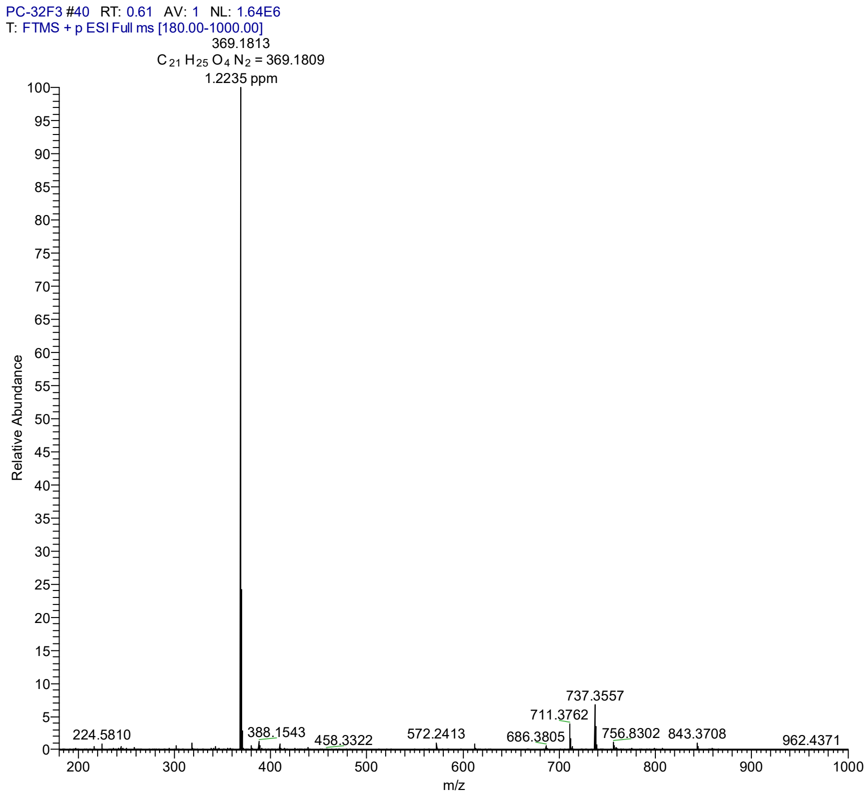

Figure S10. HRESIMS spectrum of compound **1.**

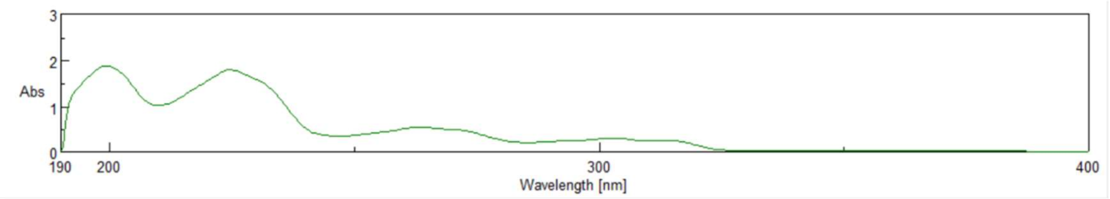

Figure S11. UV spectrum of compound **1.**

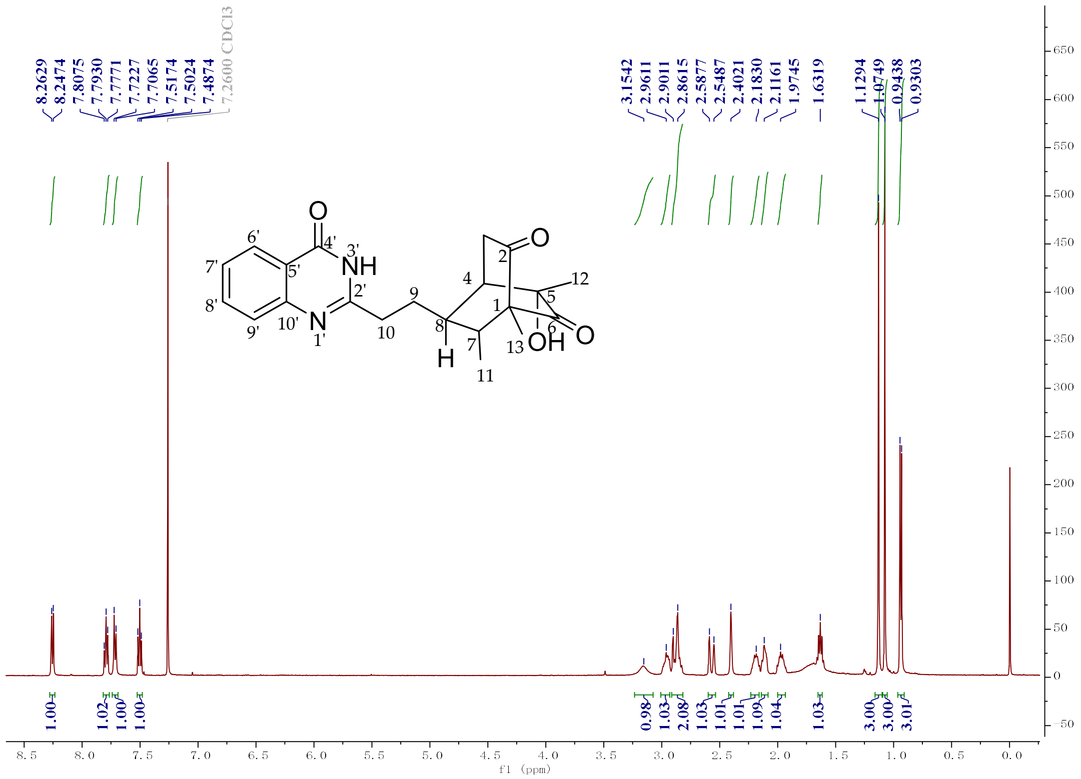

Figure S12. ^{1}H NMR spectrum of compound **1** in $CDCl_3$, 500MHz.

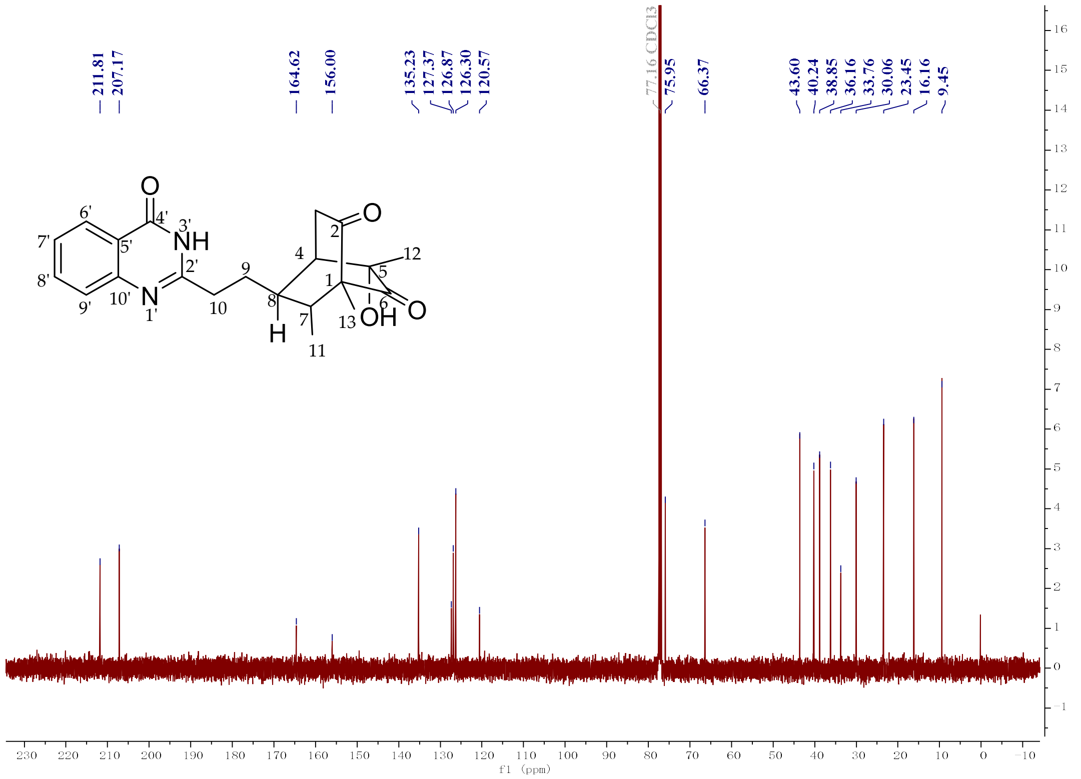

Figure S13. ^{13}C NMR spectrum of compound **1** in $CDCl_3$, 125MHz.

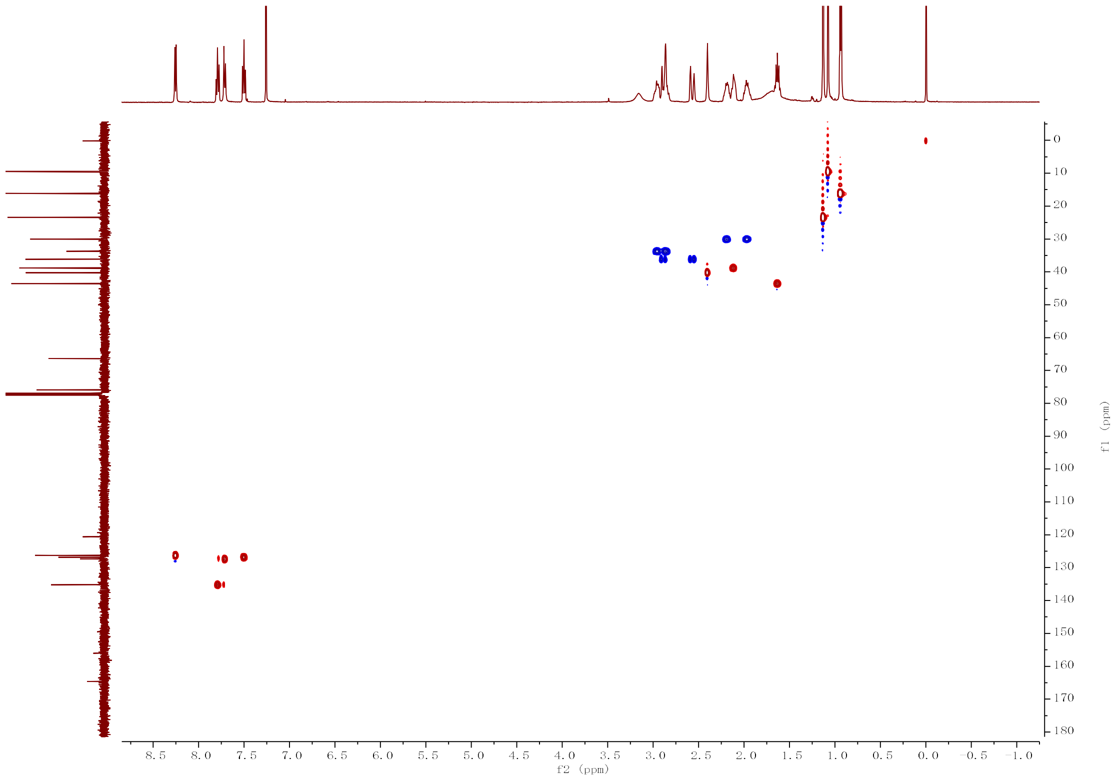

Figure S14. HSQC spectrum of compound **1** in $CDCl_3$, 500MHz.

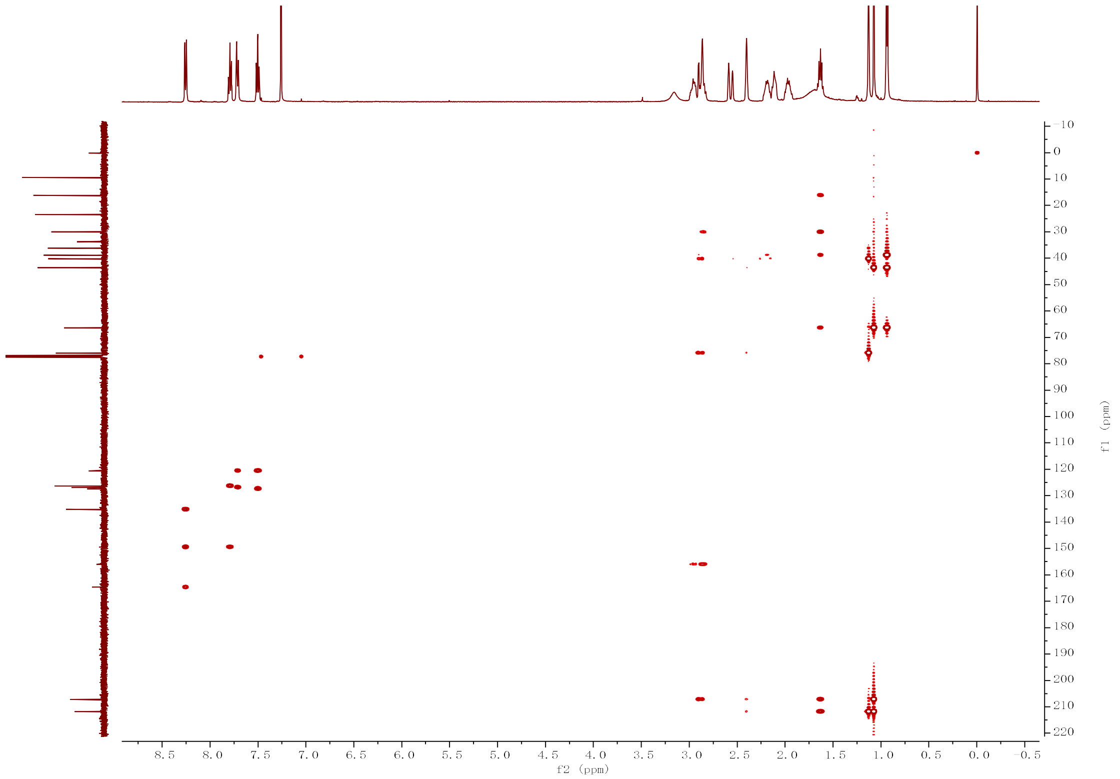

Figure S15. HMBC spectrum of compound **1** in $CDCl_3$, 500MHz.

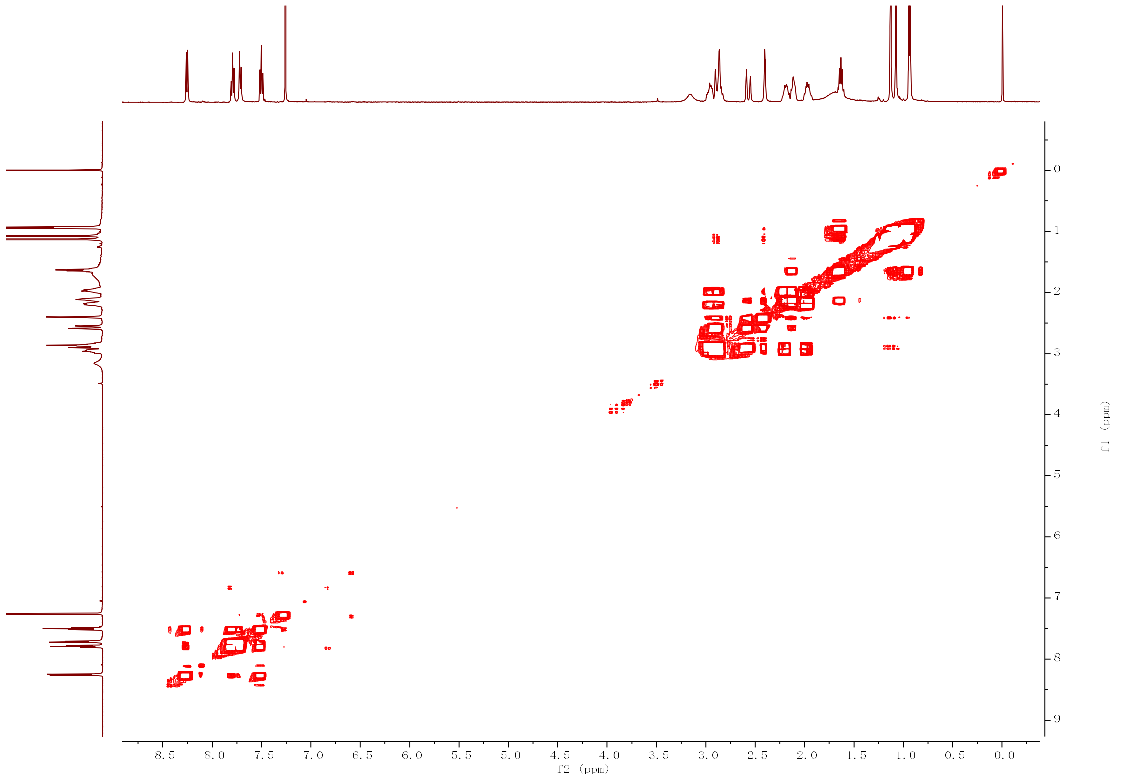

Figure S16. ^1H–^1H COSY spectrum of compound **1** in $CDCl_3$, 500MHz.

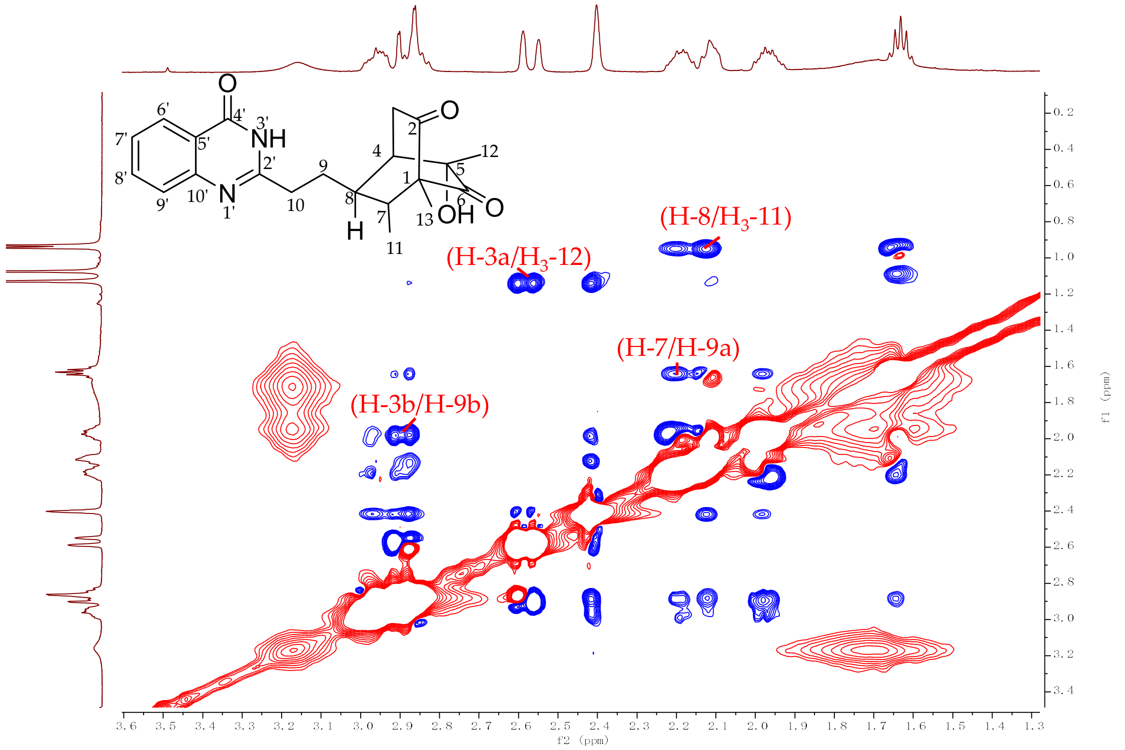

Figure S17. NOESY spectrum of compound **1** in $CDCl_3$, 500MHz.

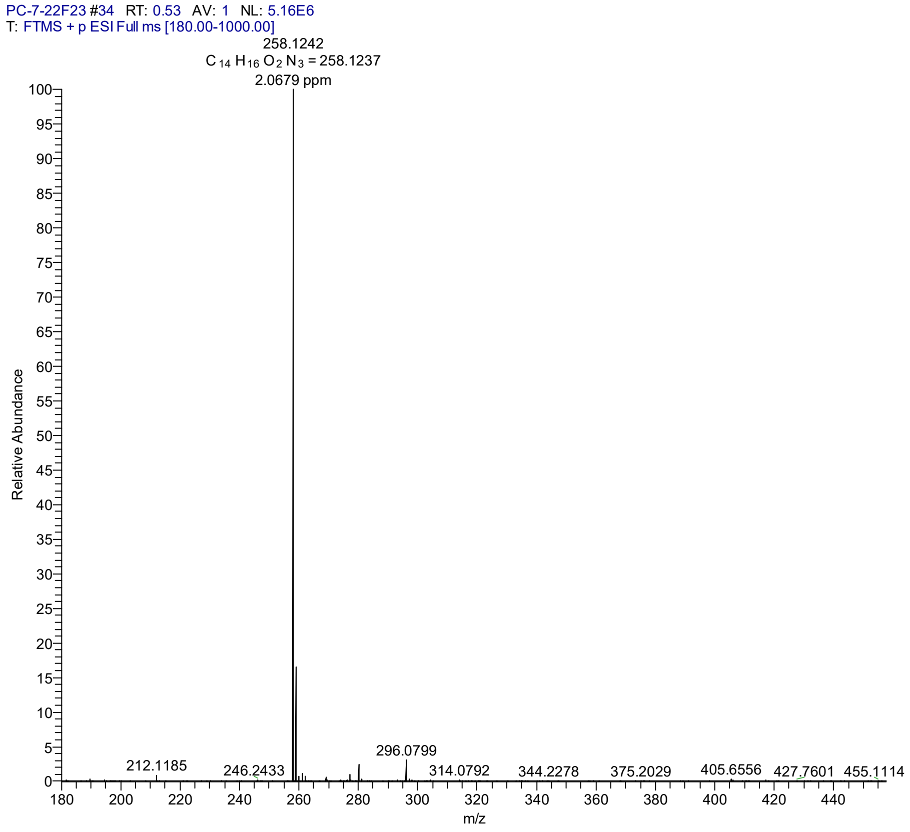

Figure S18. HRESIMS spectrum of compound **2.**

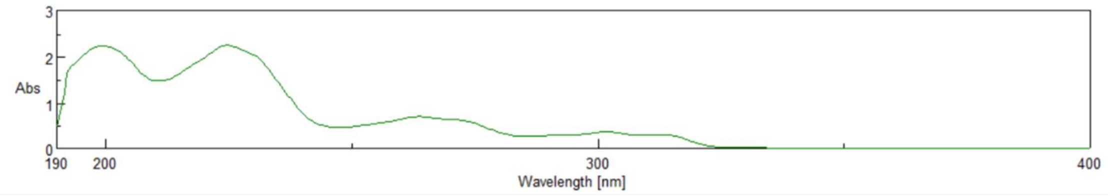

Figure S19. UV spectrum of compound **2.**

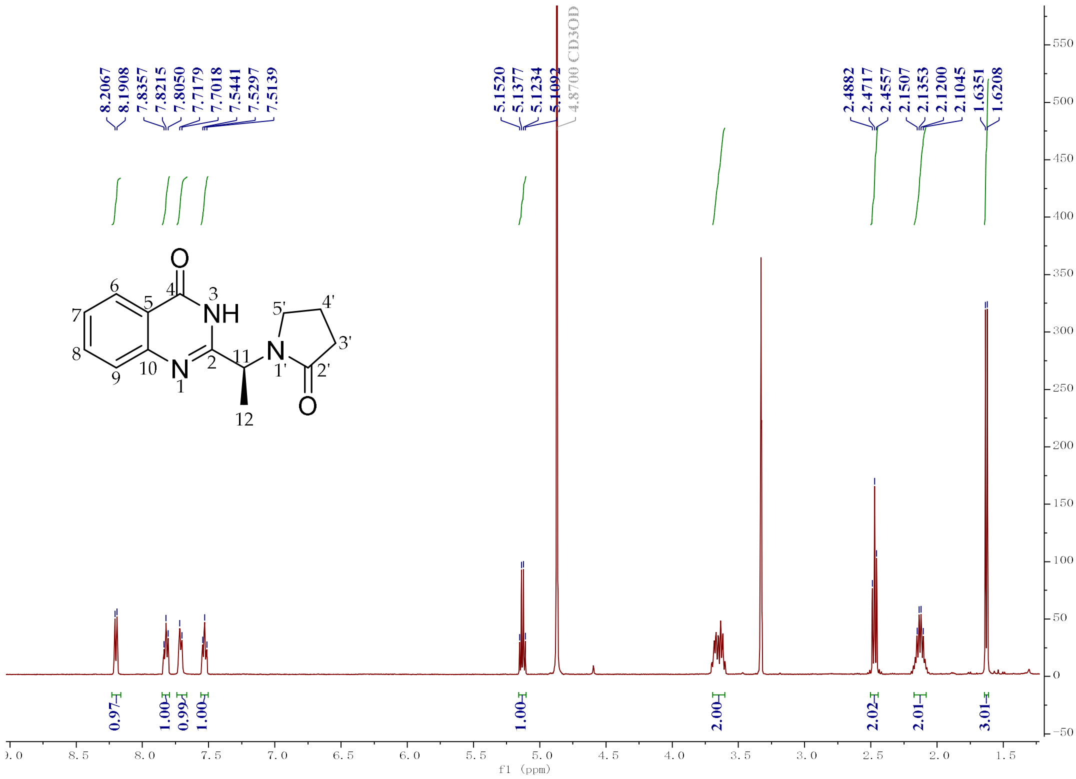

Figure S20. ^{1}H NMR spectrum of compound **2** in CD_3OD, 500MHz.

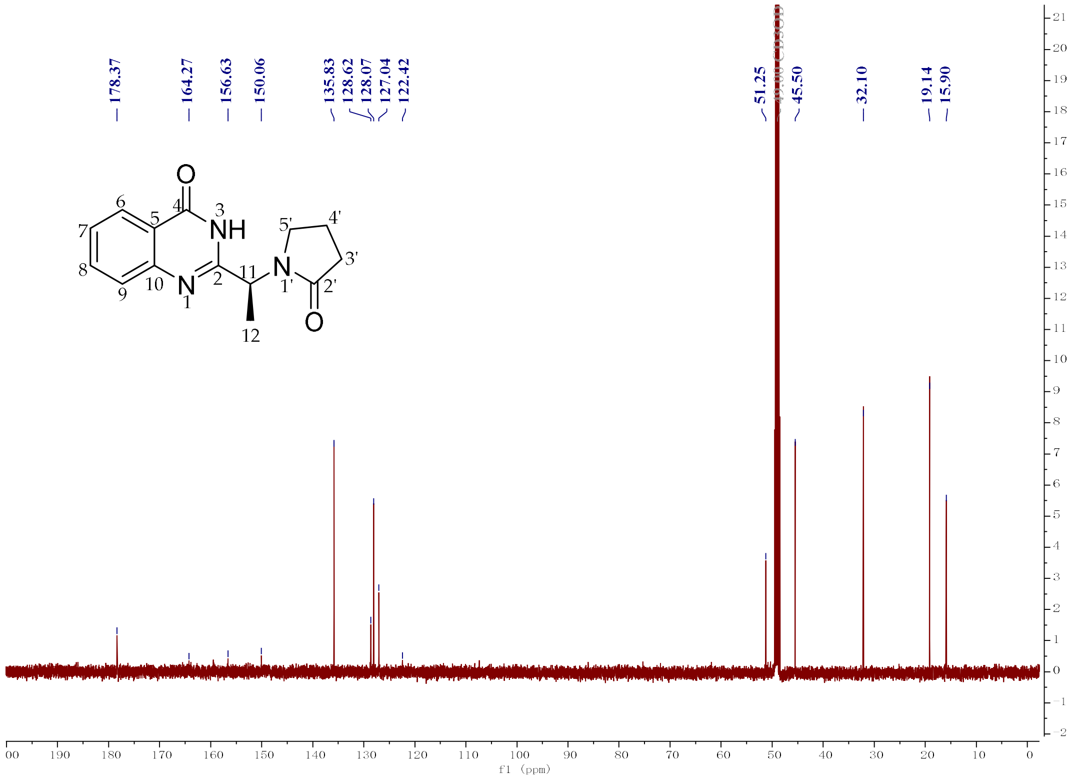

Figure S21. ^{13}C NMR spectrum of compound **2** in CD_3OD, 125MHz.

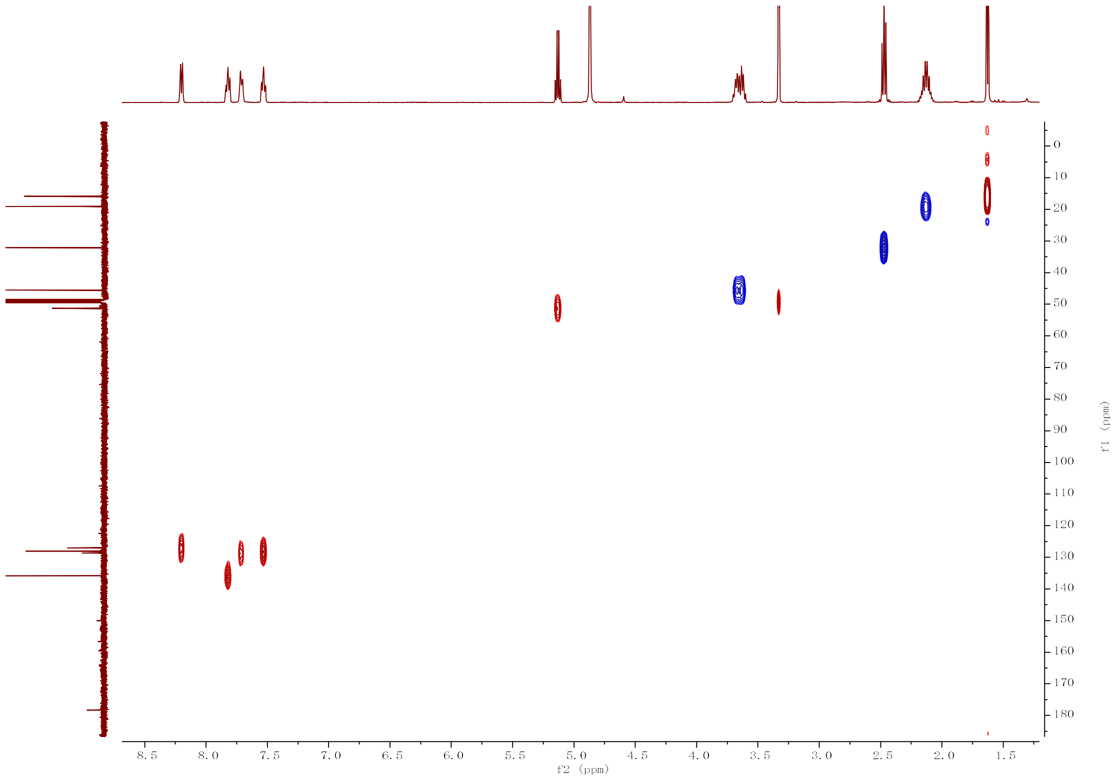

Figure S22. HSQC spectrum of compound **2** in CD3OD, 500MHz.

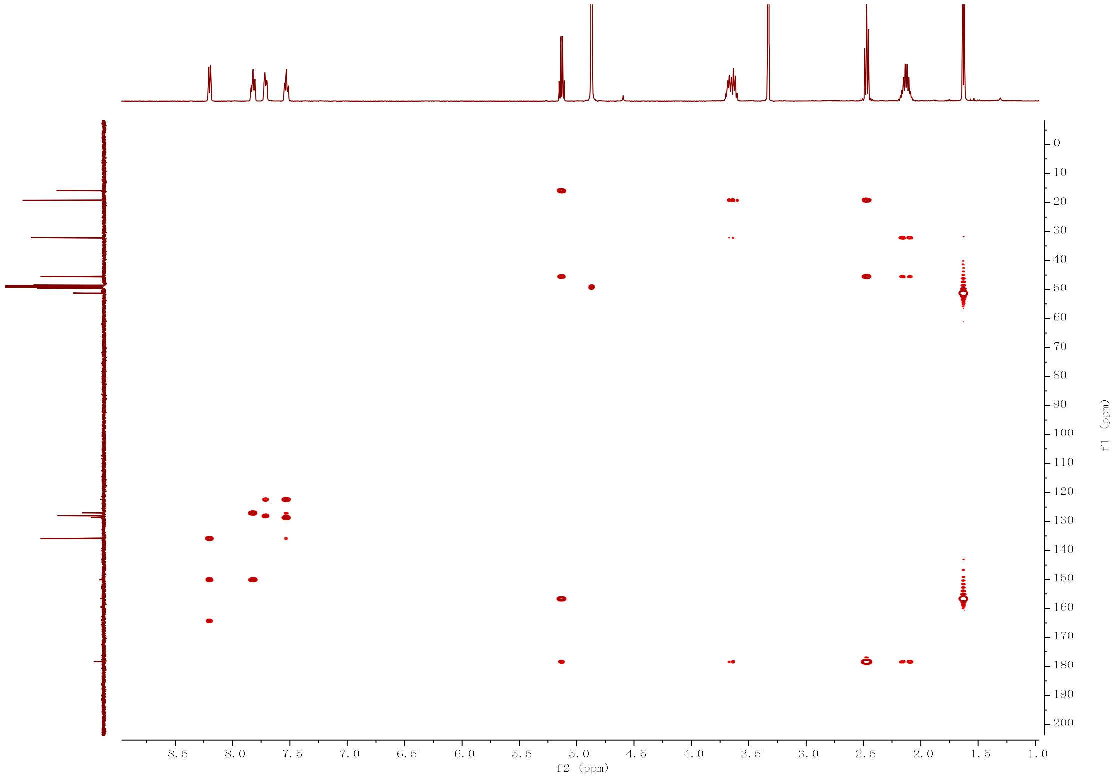

Figure S23. HMBC spectrum of compound **2** in CD3OD, 500MHz.

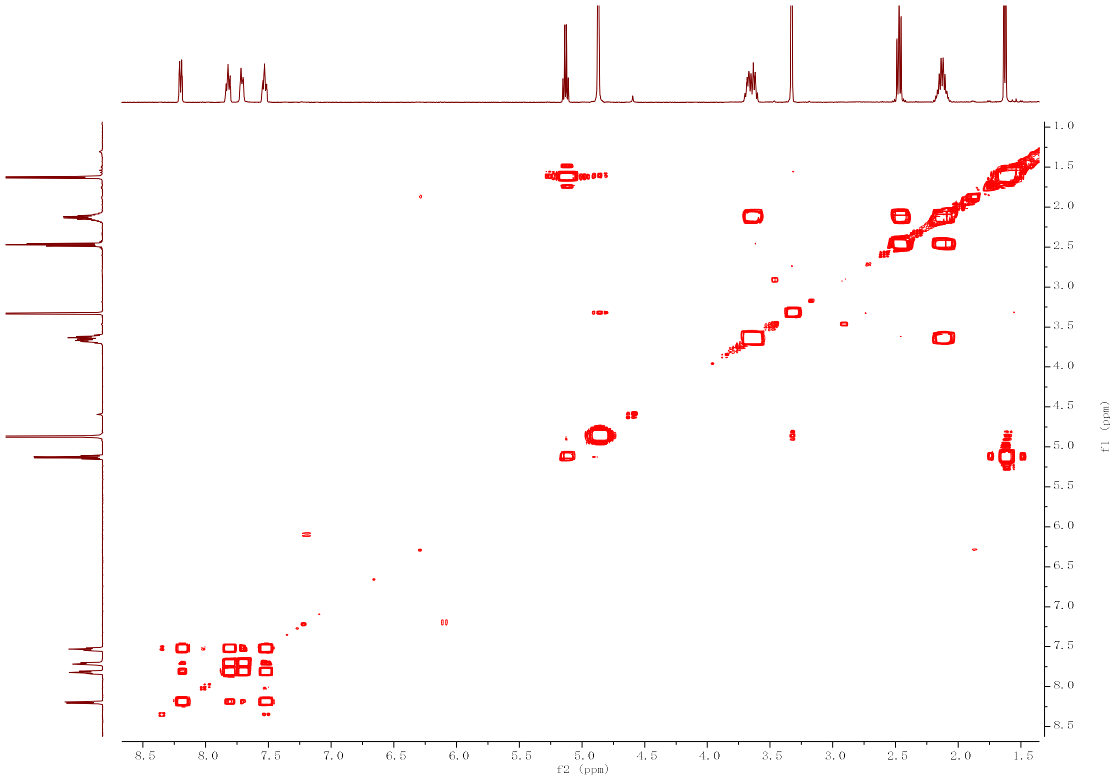

Figure S24. ^1H–^1H COSY spectrum of compound **2** in CD_3OD, 500MHz.

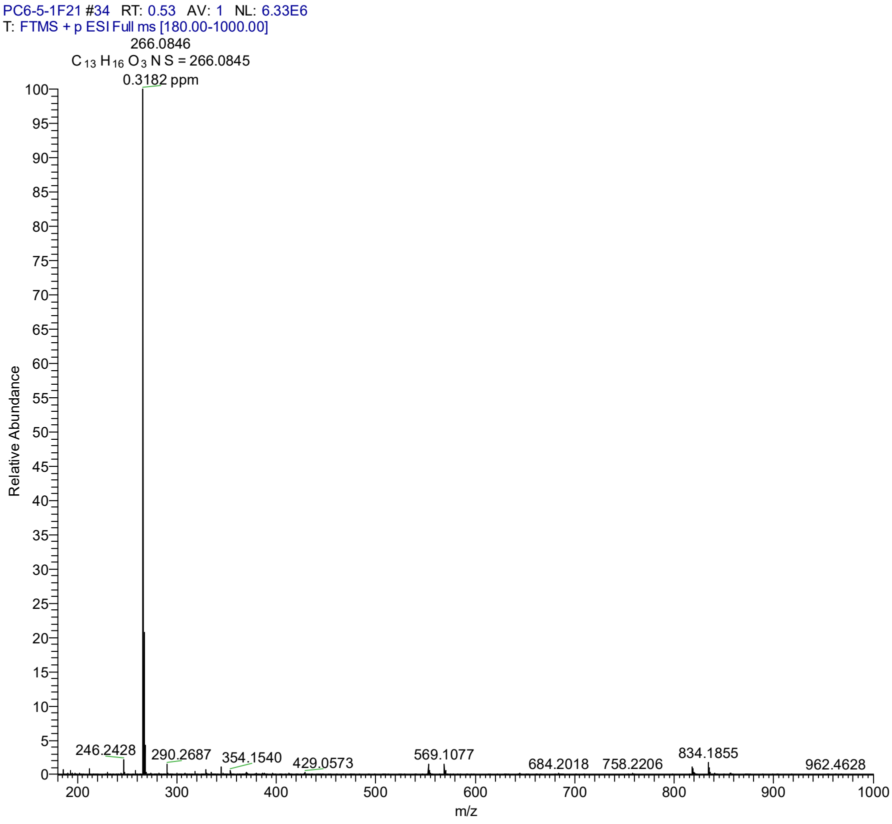

Figure S25. HRESIMS spectrum of compound **3.**

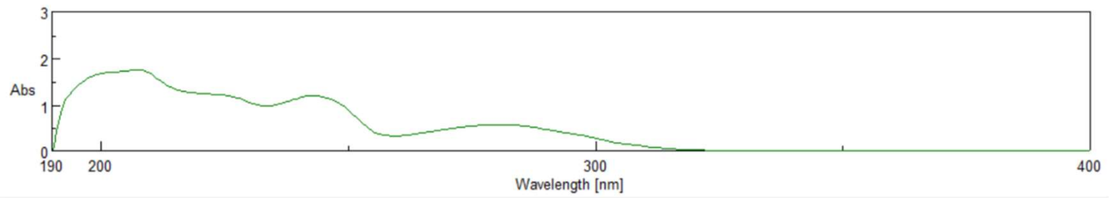

Figure S26. UV spectrum of compound **3**.

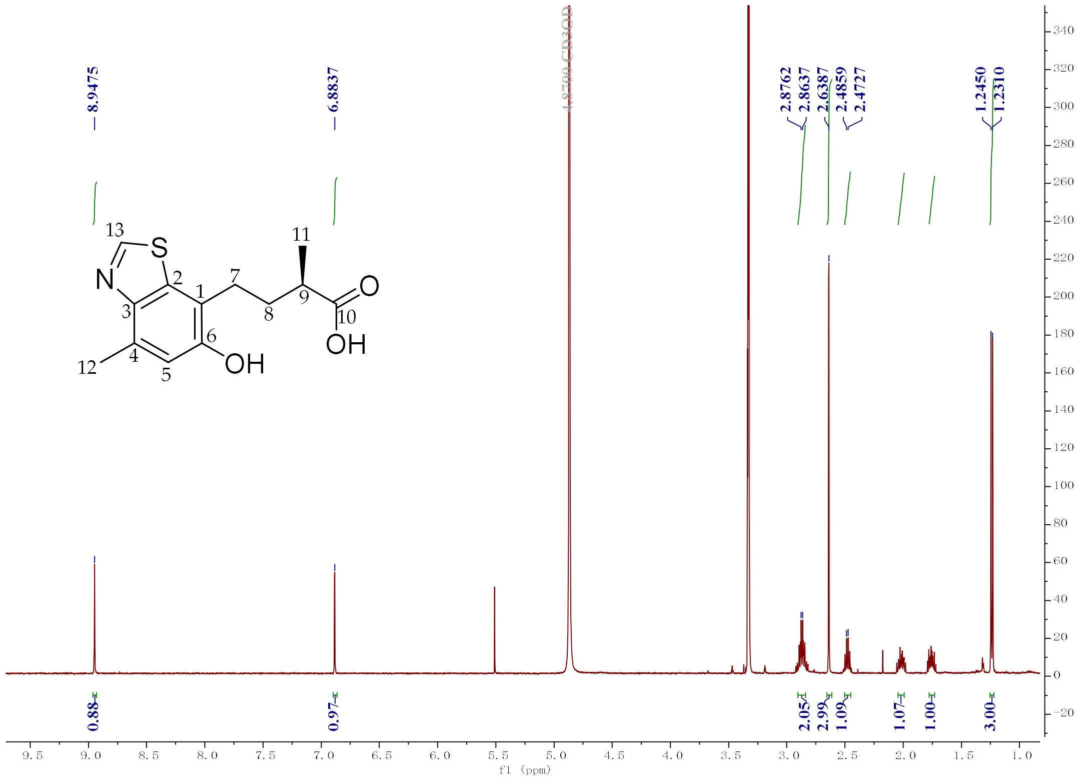

Figure S27. ¹H NMR spectrum of compound **3** in CD3OD, 500MHz.

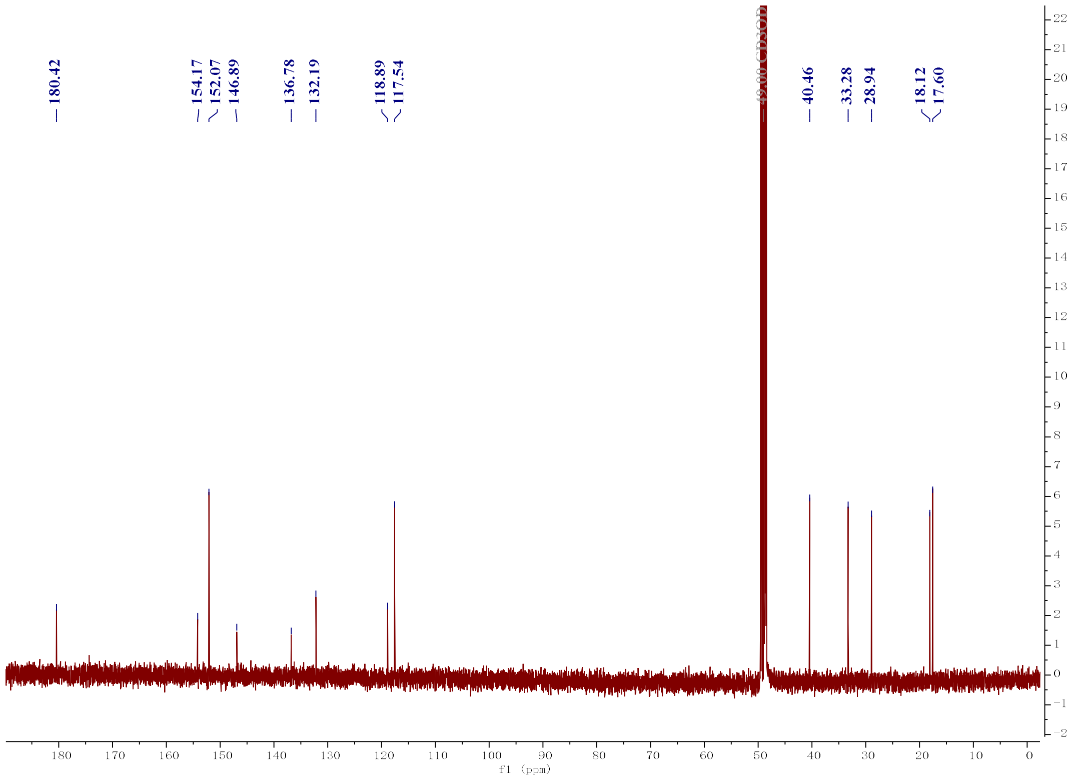

Figure S28. ^{13}C NMR spectrum of compound **3** in CD_3OD, 125MHz.

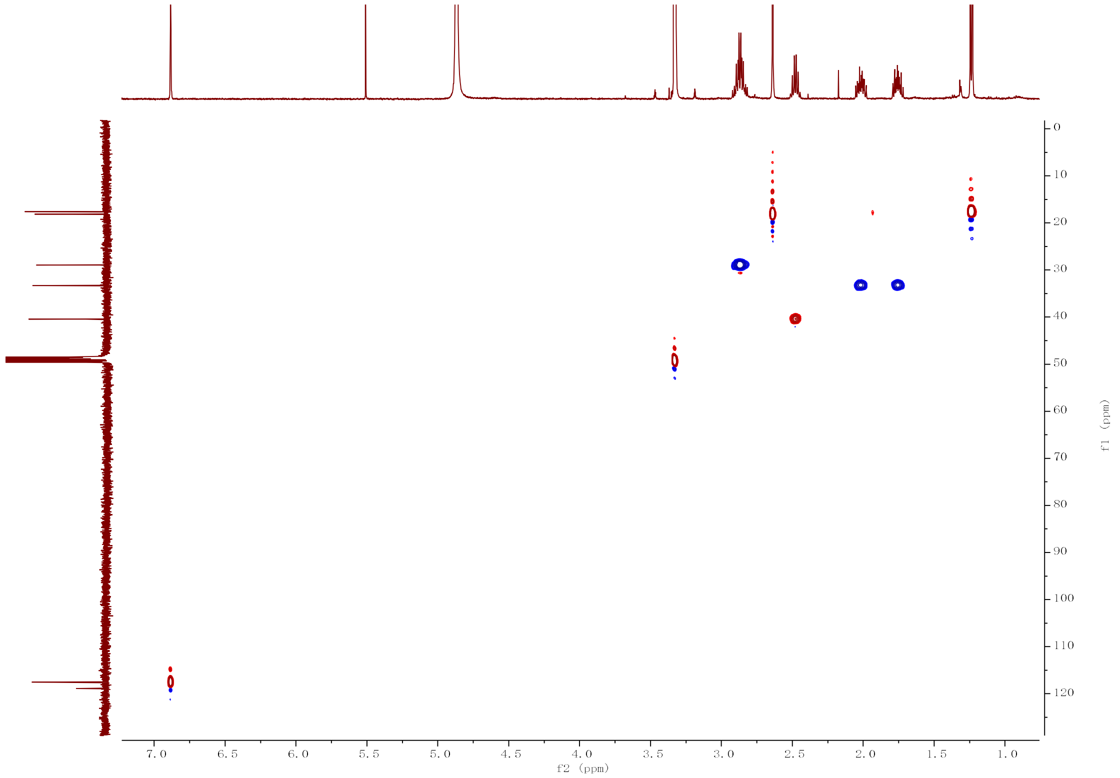

Figure S29. HSQC spectrum of compound **3** in CD_3OD, 500MHz.

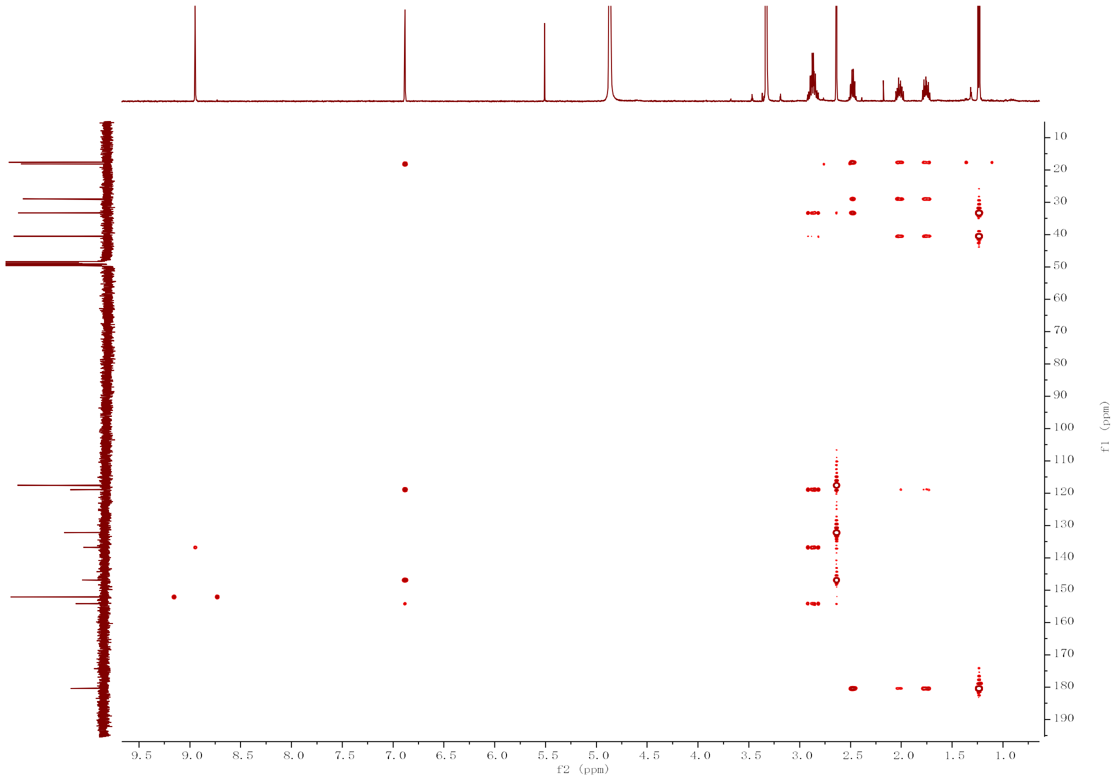

Figure S30. HMBC spectrum of compound **3** in CD_3OD, 500MHz.

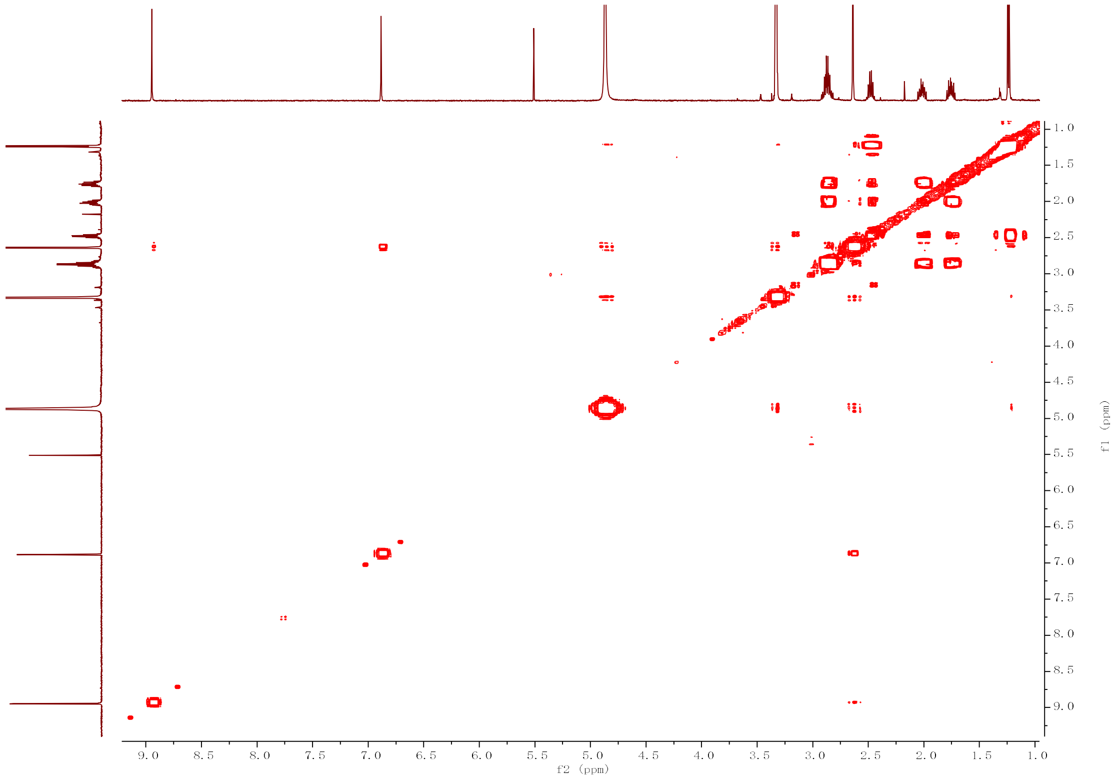

Figure S31. ^1H–^1H COSY spectrum of compound **3** in CD_3OD, 500MHz.

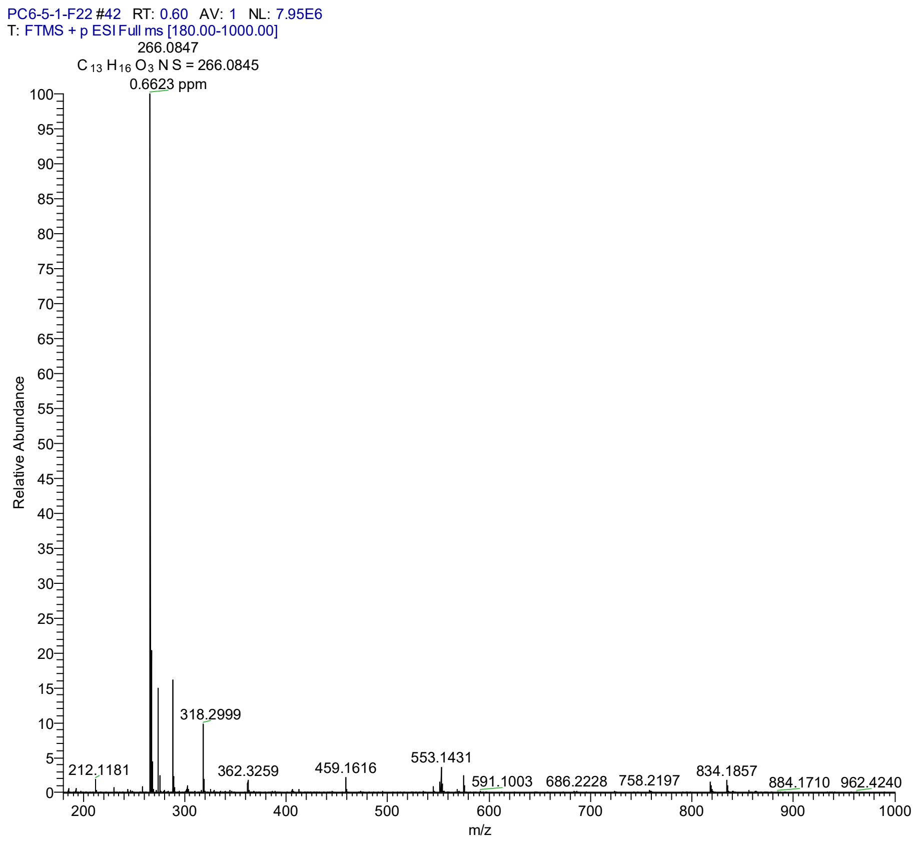

Figure S32. HRESIMS spectrum of compound **4**.

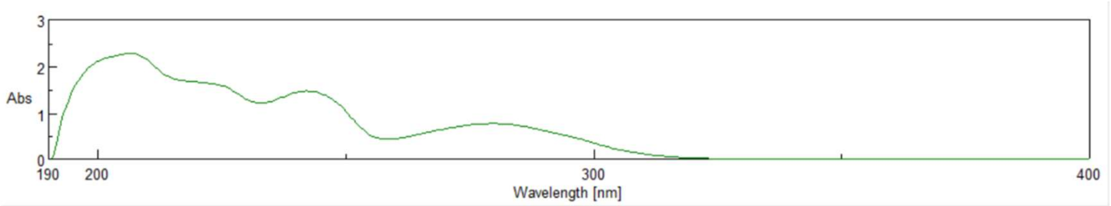

Figure S33. UV spectrum of compound **4**.

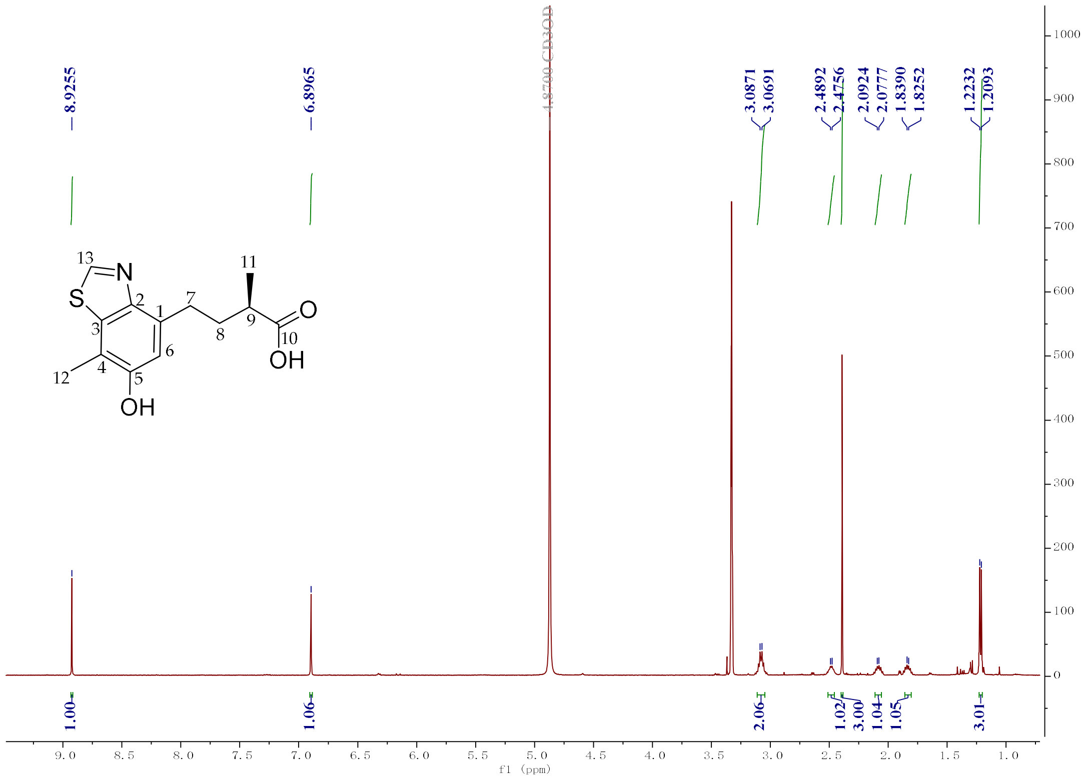

Figure S34. ^{1}H NMR spectrum of compound **4** in CD_3OD, 500MHz.

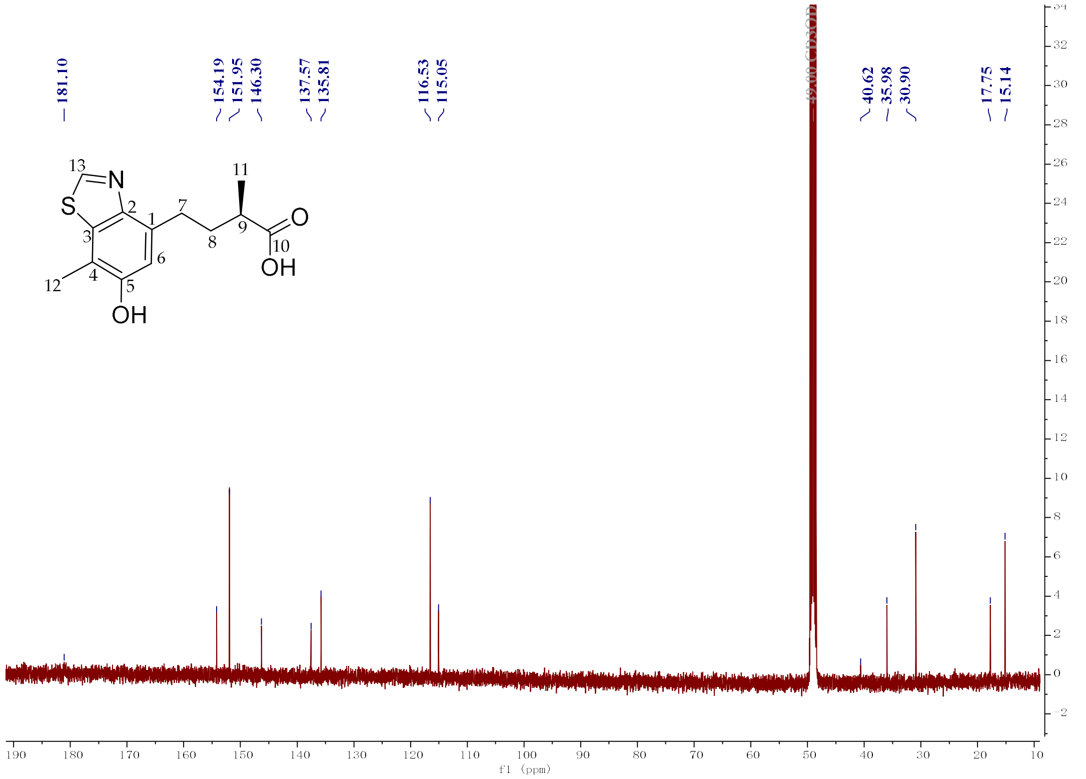

Figure S35. ^{13}C NMR spectrum of compound **4** in CD_3OD, 125MHz.

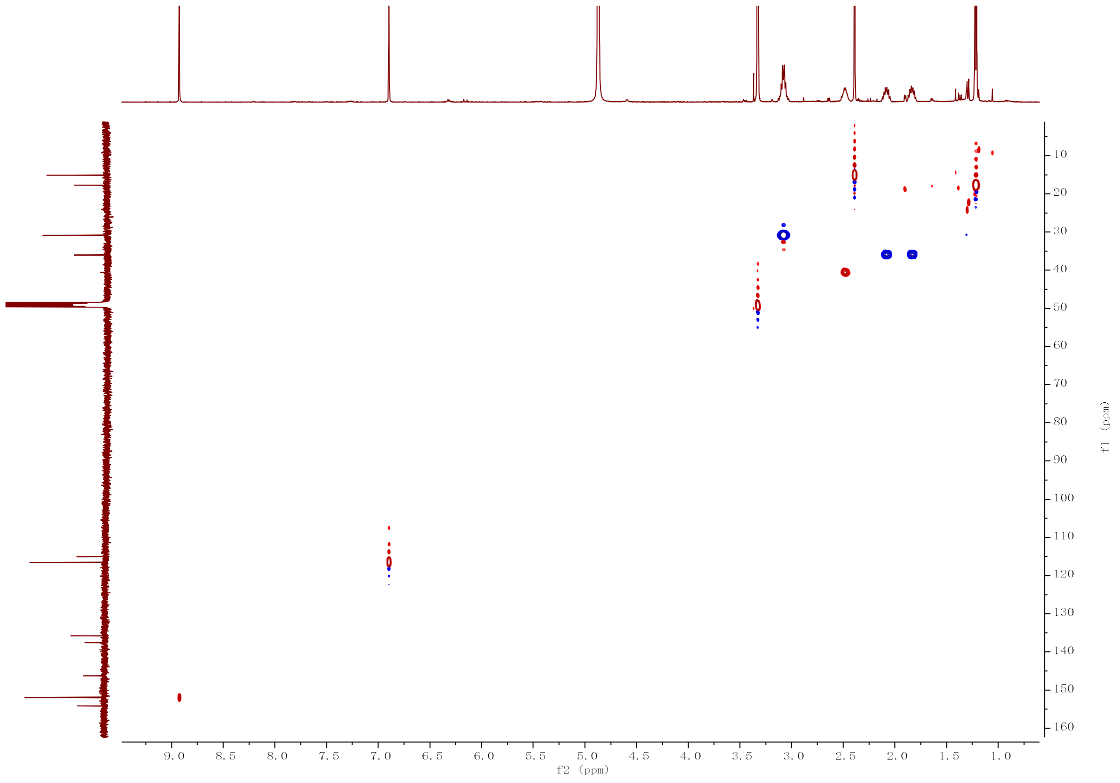

Figure S36. HSQC spectrum of compound **4** in CD3OD, 500MHz.

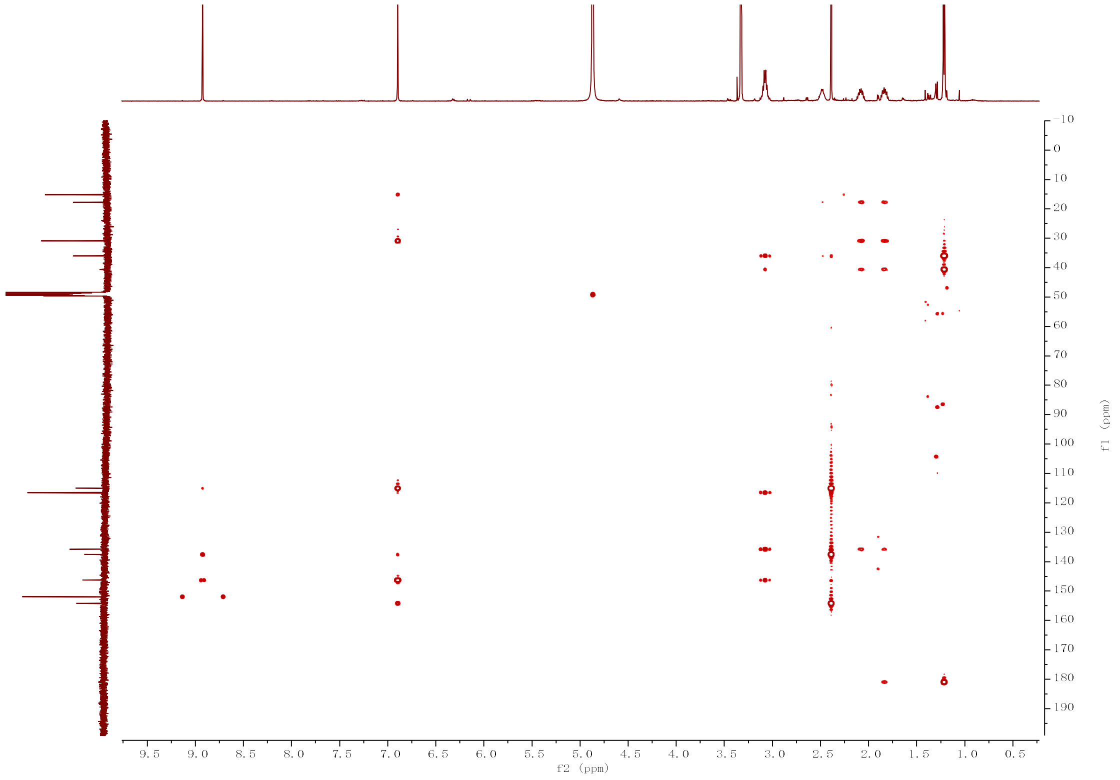

Figure S37. HMBC spectrum of compound **4** in CD3OD, 500MHz.

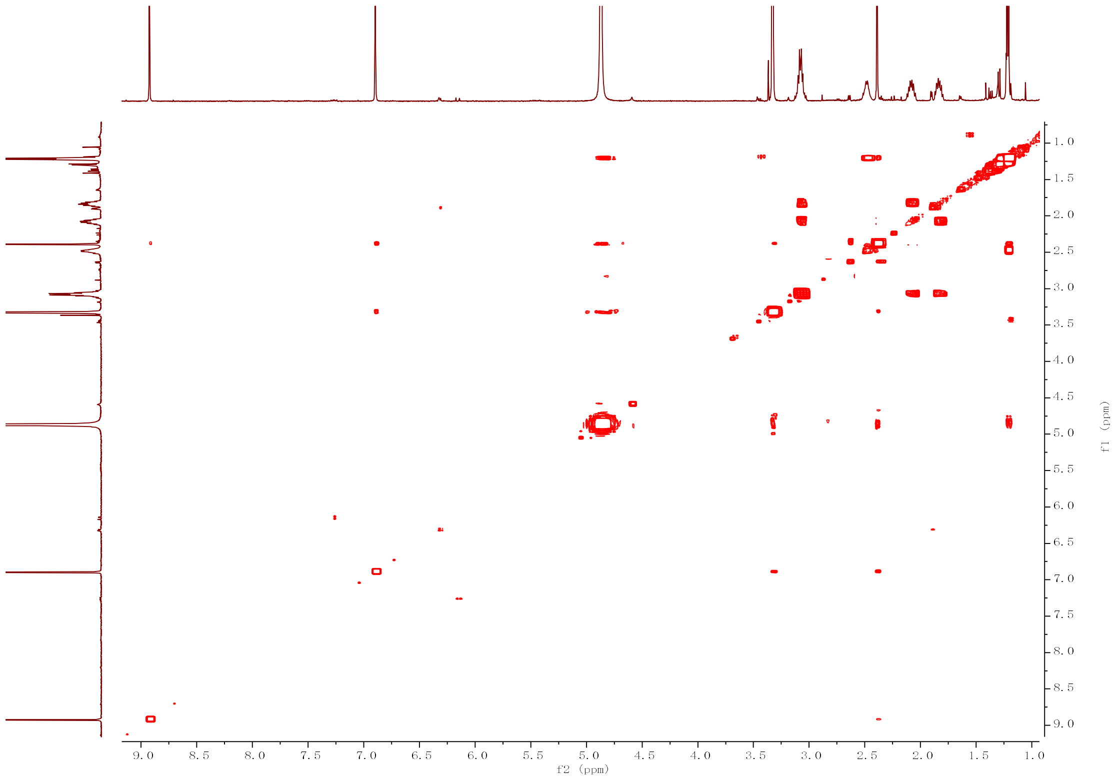

Figure S38. ^1H–^1H COSY spectrum of compound **4** in CD_3OD, 500MHz.

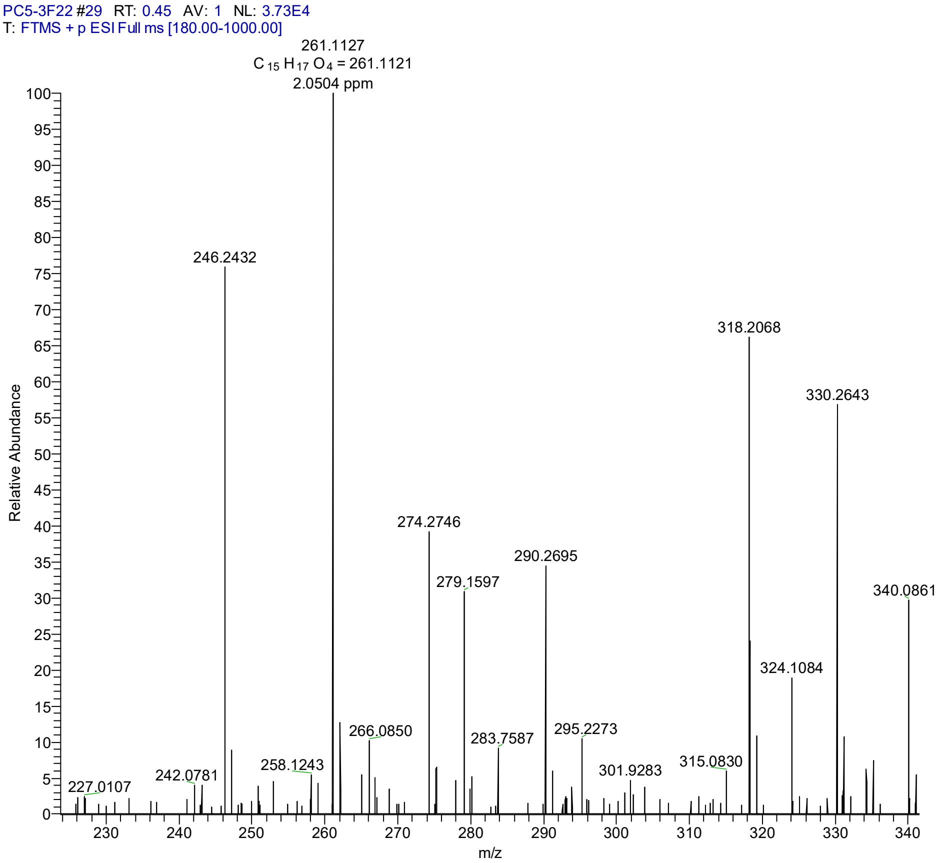

Figure S39. HRESIMS spectrum of compound **5**.

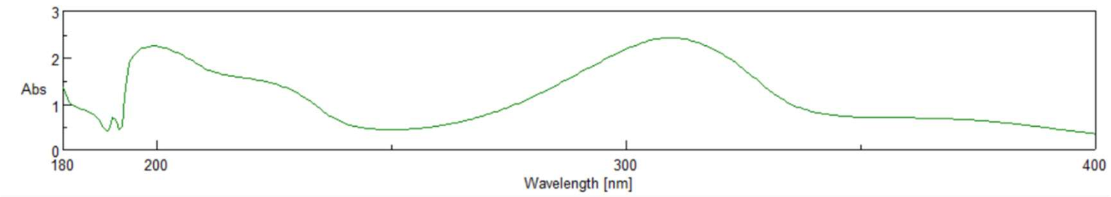

Figure S40. UV spectrum of compound **5**.

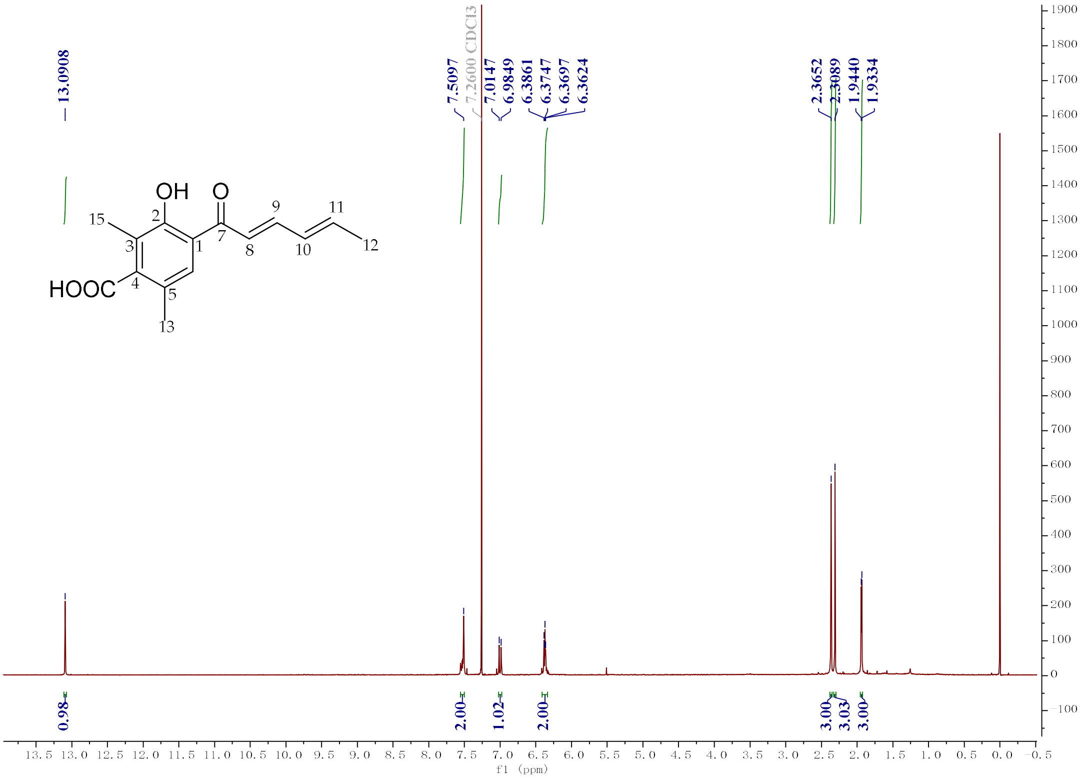

Figure S41. ¹H NMR spectrum of compound **5** in $CDCl_3$, 500MHz.

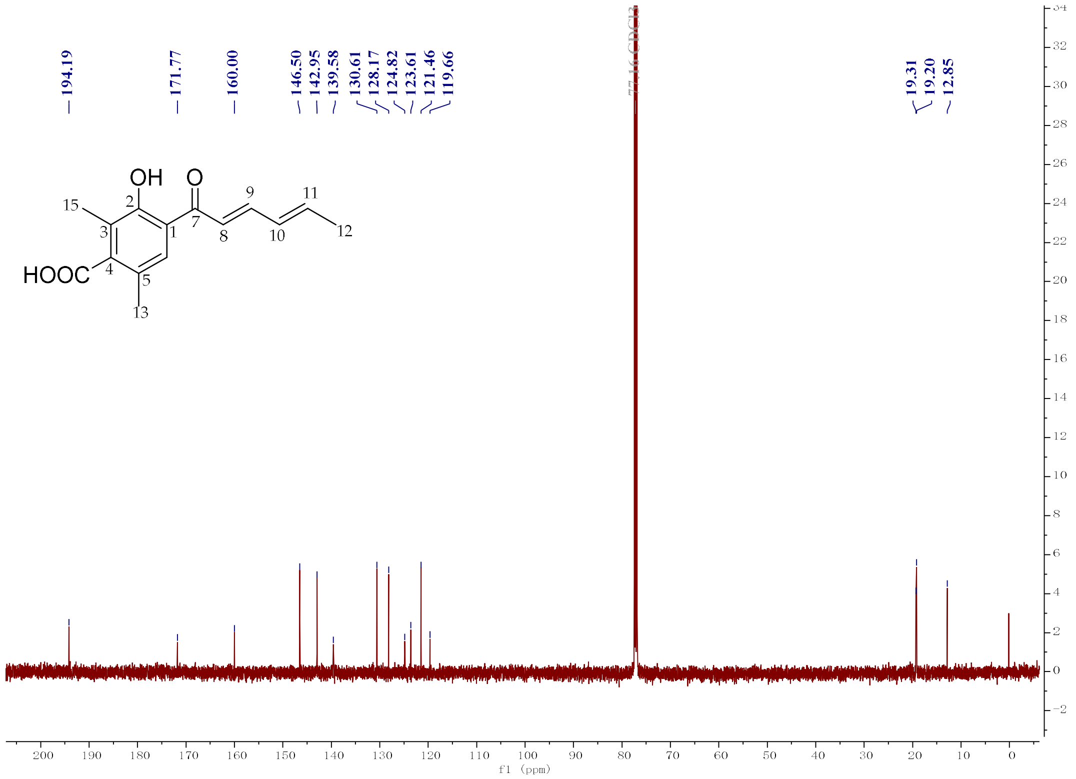

Figure S42. ^{13}C NMR spectrum of compound **5** in $CDCl_3$, 125MHz.

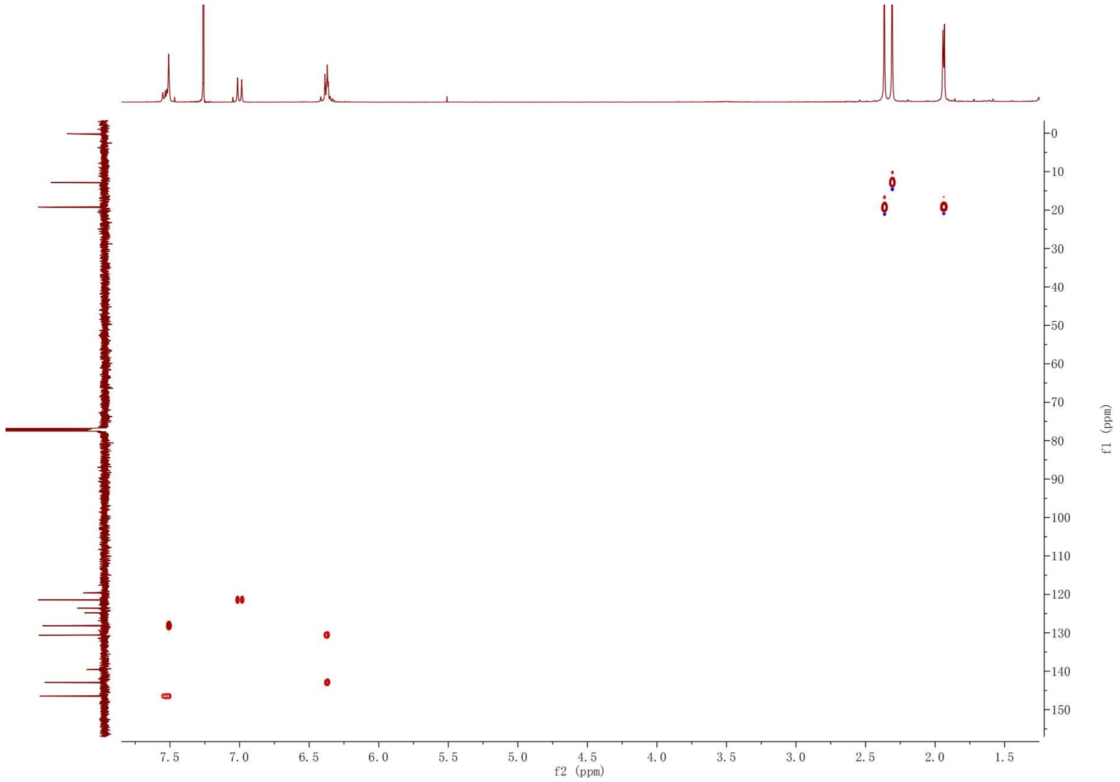

Figure S43. HSQC spectrum of compound **5** in $CDCl_3$, 500MHz.

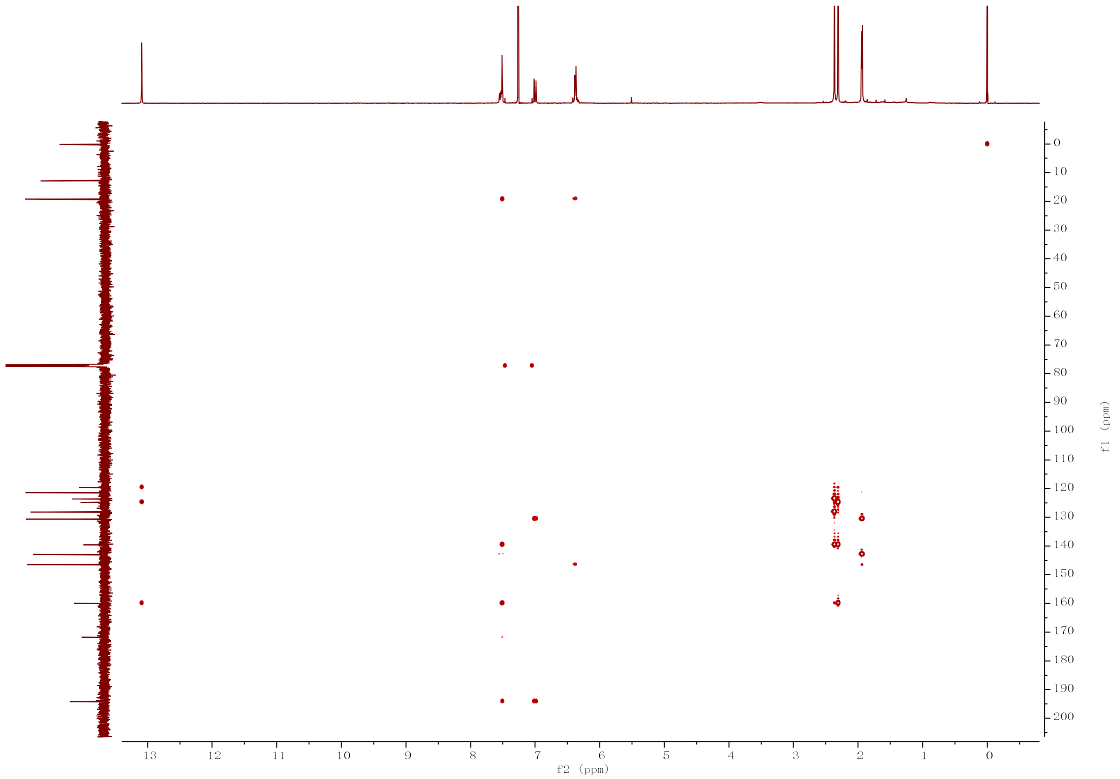

Figure S44. HMBC spectrum of compound **5** in $CDCl_3$, 500MHz.

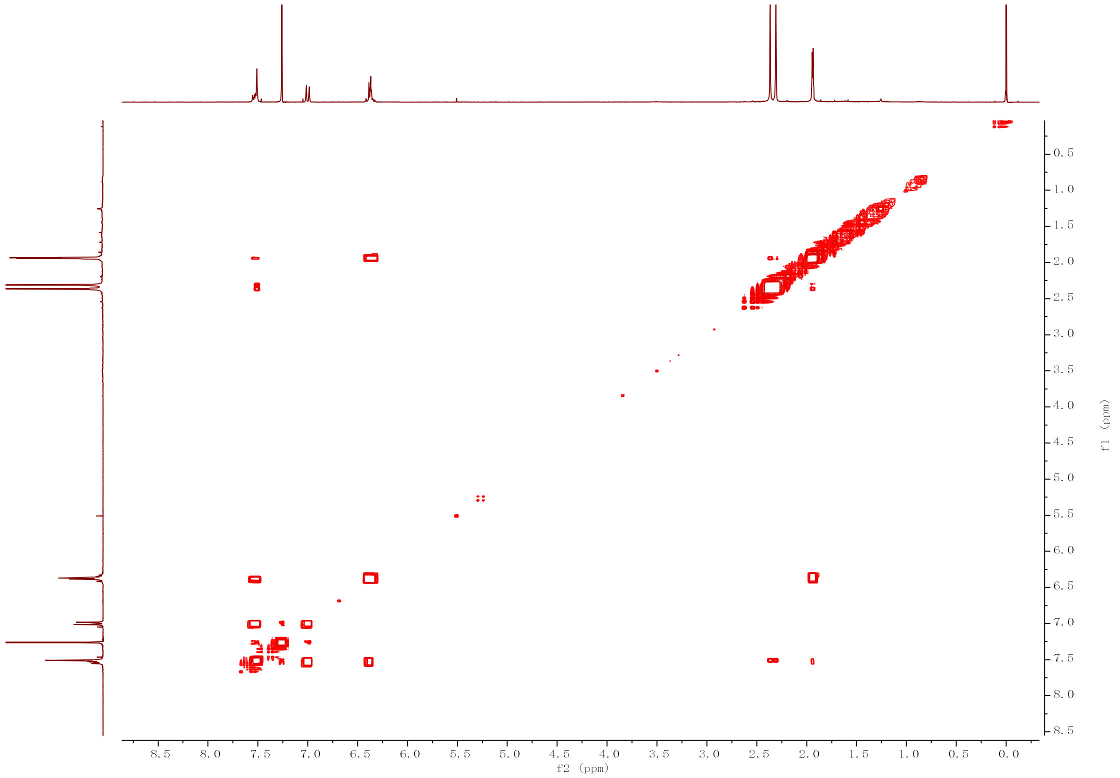

Figure S45. ^1H–^1H COSY spectrum of compound **5** in $CDCl_3$, 500MHz.
